# Global mortality from dementia: Application of a new method and results from the Global Burden of Disease Study 2019

**DOI:** 10.1002/trc2.12200

**Published:** 2021-07-27

**Authors:** GBD 2019 Collaborators, Emma Nichols, Foad Abd‐Allah, Amir Abdoli, Akine Eshete Abosetugn, Woldu Aberhe Abrha, Ahmed Abualhasan, Eman Abu‐Gharbieh, Rufus Olusola Akinyemi, Fares Alahdab, Fahad Mashhour Alanezi, Vahid Alipour, Iman Ansari, Jalal Arabloo, Amir Ashraf‐Ganjouei, Abolfazl Avan, Getinet Ayano, Zaheer‐Ud‐Din Babar, Atif Amin Baig, Maciej Banach, Miguel A. Barboza, Suzanne Lyn Barker‐Collo, Bernhard T. Baune, Akshaya Srikanth Bhagavathula, Krittika Bhattacharyya, Ali Bijani, Antonio Biondi, Tsegaye Adane Birhan, Atanu Biswas, Srinivasa Rao Bolla, Archith Boloor, Carol Brayne, Hermann Brenner, Katrin Burkart, Richard A. Burns, Sharath Burugina Nagaraja, Felix Carvalho, Luis F. S. Castro‐de‐Araujo, Ferrán Catalá‐López, Ester Cerin, Achille Cernigliaro, Nicolas Cherbuin, Jee‐Young Jasmine Choi, Dinh‐Toi Chu, Baye Dagnew, Xiaochen Dai, Lalit Dandona, Rakhi Dandona, Daniel Diaz, Zahra Sadat Dibaji Forooshani, Abdel Douiri, Bruce B. Duncan, David Edvardsson, Shaimaa I. El‐Jaafary, Khalil Eskandari, Sharareh Eskandarieh, Valery L. Feigin, Seyed‐Mohammad Fereshtehnejad, Eduarda Fernandes, Pietro Ferrara, Irina Filip, Florian Fischer, Shilpa Gaidhane, Kidane Zereabruk Gebregzabiher, Ahmad Ghashghaee, Asadollah Gholamian, Elena V. Gnedovskaya, Mahaveer Golechha, Rajeev Gupta, Vladimir Hachinski, Samer Hamidi, Graeme J. Hankey, Josep Maria Haro, Amr Hassan, Simon I. Hay, Golnaz Heidari, Reza Heidari‐Soureshjani, Mowafa Househ, Rabia Hussain, Bing‐Fang Hwang, Irena M. Ilic, Milena D. Ilic, Seyed Sina Naghibi Irvani, Hiroyasu Iso, Masao Iwagami, Ravi Prakash Jha, Rizwan Kalani, Himal Kandel, André Karch, Ayele Semachew Kasa, Andre Pascal Kengne, Young‐Eun Kim, Yun Jin Kim, Sezer Kisa, Adnan Kisa, Mika Kivimäki, Hamidreza Komaki, Ai Koyanagi, Walter A Kukull, G. Anil Kumar, Manasi Kumar, Iván Landires, Matilde Leonardi, Stephen S. Lim, Xuefeng Liu, Giancarlo Logroscino, Alan D. Lopez, Stefan Lorkowski, Clement T. Loy, Hawraz Ibrahim M. Amin, Navid Manafi, Narayana Manjunatha, Man Mohan Mehndiratta, Ritesh G Menezes, Atte Meretoja, Alexander Merkin, Workua Mekonnen Metekiya, Awoke Temesgen Misganaw, Bahram Mohajer, Norlinah Mohamed Ibrahim, Yousef Mohammad, Archisman Mohapatra, Farnam Mohebi, Ali H. Mokdad, Stefania Mondello, Tilahun Belete Mossie, Anwar Mulugeta, Gabriele Nagel, Muhammad Naveed, Vinod C. Nayak, Sandhya Neupane Kandel, Son Hoang Nguyen, Huong Lan Thi Nguyen, Virginia Nuñez‐Samudio, Felix Akpojene Ogbo, Andrew T. Olagunju, Hans Orru, Sergej M. Ostojic, Samuel M. Ostroff, Nikita Otstavnov, Stanislav S Otstavnov, Mayowa O. Owolabi, Mona Pathak, Hamidreza Pazoki Toroudi, Carrie B. Peterson, Hai Quang Pham, Michael R. Phillips, Michael A. Piradov, Faheem Hyder Pottoo, Sergio I. Prada, Dimas Ria Angga Pribadi, Amir Radfar, Alberto Raggi, Fakher Rahim, Pradhum Ram, Juwel Rana, Vahid Rashedi, Salman Rawaf, David Laith Rawaf, Nickolas Reinig, Nima Rezaei, Stephen R Robinson, Michele Romoli, Perminder S. Sachdev, Ramesh Sahathevan, Amirhossein Sahebkar, Mohammad Ali Sahraian, Davide Sattin, Mete Saylan, Mehdi Sayyah, Silvia Schiavolin, Maria Inês Schmidt, Izza Shahid, Masood Ali Shaikh, Mika Shigematsu, Jae Il Shin, Rahman Shiri, Tariq Jamal Siddiqi, João Pedro Silva, Jasvinder A. Singh, Amin Soheili, Emma Elizabeth Spurlock, Cassandra E I Szoeke, Rafael Tabarés‐Seisdedos, Biruk Wogayehu Taddele, Bhaskar Thakur, Akhil Soman ThekkePurakkal, Marcos Roberto Tovani‐Palone, Bach Xuan Tran, Ravensara S. Travillian, Manjari Tripathi, Gebiyaw Wudie Tsegaye, Muhammad Shariq Usman, Marco Vacante, Diana Zuleika Velazquez, Narayanaswamy Venketasubramanian, Simone Vidale, Vasily Vlassov, Yuan‐Pang Wang, Jingkai Wei, Jordan Weiss, Abrha Hailay Weldemariam, Anders Wimo, Chenkai Wu, Ali Yadollahpour, Kazumasa Yamagishi, Yordanos Gizachew Yeshitila, Naohiro Yonemoto, Siddhesh Zadey, Zhi‐Jiang Zhang, Christopher J. L. Murray, Theo Vos

**Affiliations:** ^1^ Institute for Health Metrics and Evaluation Seattle Washington USA

**Keywords:** burden of disease, dementia, global health, mortality

## Abstract

**Introduction:**

Dementia is currently one of the leading causes of mortality globally, and mortality due to dementia will likely increase in the future along with corresponding increases in population growth and population aging. However, large inconsistencies in coding practices in vital registration systems over time and between countries complicate the estimation of global dementia mortality.

**Methods:**

We meta‐analyzed the excess risk of death in those with dementia and multiplied these estimates by the proportion of dementia deaths occurring in those with severe, end‐stage disease to calculate the total number of deaths that could be attributed to dementia.

**Results:**

We estimated that there were 1.62 million (95% uncertainty interval [UI]: 0.41–4.21) deaths globally due to dementia in 2019. More dementia deaths occurred in women (1.06 million [0.27–2.71]) than men (0.56 million [0.14–1.51]), largely but not entirely due to the higher life expectancy in women (age‐standardized female‐to‐male ratio 1.19 [1.10–1.26]). Due to population aging, there was a large increase in all‐age mortality rates from dementia between 1990 and 2019 (100.1% [89.1–117.5]). In 2019, deaths due to dementia ranked seventh globally in all ages and fourth among individuals 70 and older compared to deaths from other diseases estimated in the Global Burden of Disease (GBD) study.

**Discussion:**

Mortality due to dementia represents a substantial global burden, and is expected to continue to grow into the future as an older, aging population expands globally.

## 1 INTRODUCTION

While there is a wealth of evidence indicating that individuals with dementia have a higher risk for mortality, the mechanisms by which dementia leads to death are less clear.^1^ The International Classification of Disease (ICD) guidelines on the certification of causes of death define the underlying cause of death as the condition which gave rise to all other conditions leading to the death of the individual.^2^ Prior evidence indicates that conditions such as bronchopneumonia and pulmonary embolisms are often the immediate causes of death among those with dementia and that these conditions are more frequent in those with dementia than in those without dementia, suggesting that dementia may actually be the underlying cause of death in many of these cases.^3^, ^4^, ^5^ However, oftentimes, these immediate causes of death are listed on the death certificate without mention of dementia.^6^

Due to these challenges in the certification of deaths due to dementia, the use of vital registration data for the estimation of mortality due to dementia is highly susceptible to changes over time in coding practices that exist within and between vital registration systems and the societies in which they operate. Previous studies have shown increases over time in the certification of dementia as a cause of death on death certificates, while the estimated prevalence of the disease has remained stable or even decreased.^7^, ^8^, ^9^ Comparisons of the certification of dementia deaths on death certificates with information on dementia patients from population‐based epidemiological studies have indicated that dementia has been previously underreported on death certificates.^6^, ^10^, ^11^, ^12^, ^13^

Published estimates of dementia mortality are largely from the United States and Western Europe and have either focused on the estimation of mortality in those with dementia rather than mortality that can be ascribed to dementia as the underlying cause, or have relied on the generalization of prevalence and mortality risk estimates from one population‐based cohort to the entire country.^14^, ^15^, ^16^ The only available global estimates of dementia mortality are from previous iterations of the Global Burden of Disease (GBD) study. However, these estimates are subject to a number of known limitations. For GBD 2010, estimates were based on vital registration data.^17^ The recognition of the biases that exist in reporting dementia as a cause of death in vital registration data led to a new methodology for the estimation of dementia mortality, introduced in GBD 2015. For GBD 2015, 2016, and 2017, we used estimates of excess mortality derived from countries most likely to code to dementia as a cause of death per prevalent dementia case, and prevalence estimates to calculate mortality due to dementia.^18^, ^19^ While this method allowed for the global estimation of dementia deaths and accounted for the under‐reporting present in vital registration data, it was sensitive to the choice of how many countries to include in the estimation of excess mortality, and it assumed that the coding practices in these countries do not under‐ or over‐assign dementia as a cause of death.

This study aims to improve on prior methods of estimation for dementia mortality and describe the results over time and across geographies. By removing all reliance on vital registration data and instead calculating mortality due to dementia as the product of total excess deaths and the proportion of these deaths in individuals with severe, end‐stage disease, this study addresses some of the primary weaknesses in previous estimation methods.

**RESEARCH IN CONTEXT**

1. Systematic review: The authors reviewed the literature using traditional sources (PubMed). Prior estimates of dementia mortality are from previous iterations of the Global Burden of Disease study or are limited in geographic scope.
2. Interpretation: Our study improves on prior methods for the estimation of mortality by removing any reliance on inconsistent vital registration data from the modeling process. We estimated there were 1.62 million (0.41–4.21) deaths due to dementia globally in 2019.
3. Future directions: Future work should seek to strengthen the analyses by incorporating additional data sources on dementia prevalence, the excess risk of death, and the proportion of individuals with end‐stage disease, both to limit the effect of data sparsity on the precision of estimates and to increase the geographic coverage of available data. More precise and accurate estimates will increase the utility of the results for the purposes of public health planning and resource allocation.

## 2 METHODS

The category of dementia as used in this article is equivalent to the GBD disease designation of “Alzheimer's disease and other dementias.” Information on general GBD methods can be found in the GBD 2019 summary papers.^20^, ^21^ This GBD study used de‐identified data, and the waiver of informed consent was reviewed and approved by the University of Washington Institutional Review Board (Study 9060).

### 2.1 Overview of analytic strategy

The goal of this study was to estimate the number of individuals globally who died of dementia as an underlying cause of death, in line with ICD‐10 principles. To conduct this analysis, we first estimated the attributable risk of all‐cause mortality via systematic review and meta‐analysis, and multiplied this estimate by the number of individuals with dementia to calculate excess deaths due to dementia. However, not all excess deaths among individuals with dementia are likely attributable to dementia as an underlying cause of death due to the presence of common comorbid conditions such as cardiovascular diseases. Therefore, to calculate deaths attributable to dementia as an underlying cause of death, we multiplied excess dementia deaths by the proportion of individuals who died with end‐stage dementia out of the total number of excess dementia deaths, using Formula 1:

(1) $$\begin{array}{ccc} & & Dementia\;Deaths\;=\;Excess\;Dementia\;Deaths*\\ & & \frac{Dementia\;Deaths\;with\;End-Stage\;Conditions}{Excess\;Dementia\;Deaths} \end{array}$$

**FIGURE 1:**
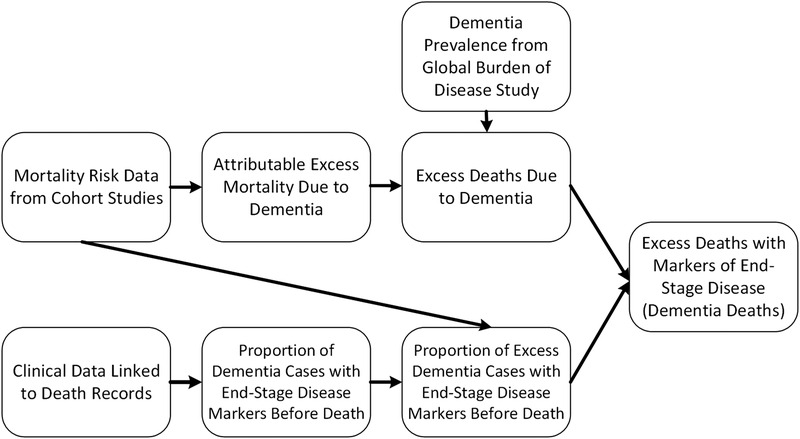
Analytical overview for the calculation of deaths attributable to dementia as an underlying cause of death. We multiplied prevalence estimates and attributable excess risk due to dementia from systematic review and meta‐analysis to calculate excess deaths due to dementia. Then, we estimated the proportion of excess dementia cases with end‐stage markers of disease markers by combining information from clinical data linked to death records and our meta‐analysis of mortality risk. We assumed deaths attributable to dementia as the underlying cause of death was the product of excess deaths due to dementia and the proportion of these deaths with markers of end‐stage disease

The overall analytic framework can be visualized in Figure 1. This strategy assumes that individuals who die with conditions signaling end‐stage dementia die from dementia as an underlying cause of death. This assumption is supported by prior research indicating that end‐stage conditions such as pneumonia, febrile illness, and eating problems are associated with extremely high short‐term mortality rates among those with advanced disease.^22^

### 2.2 Systematic review of the literature on excess mortality

Through a PubMed literature review focused on dementia excess mortality and relative risk spanning 1980 to 2018, we identified 4470 total hits, of which 34 studies were marked for extraction (additional details in supporting information).^23^, ^24^, ^25^, ^26^, ^27^, ^28^, ^29^, ^30^, ^31^, ^32^, ^33^, ^34^, ^35^, ^36^, ^37^, ^38^, ^39^, ^40^, ^41^, ^42^, ^43^, ^44^, ^45^, ^46^, ^47^, ^48^, ^49^, ^50^, ^51^, ^52^, ^53^, ^54^, ^55^, ^56^ The data sources included clinical or community‐based cohorts and excluded studies conducted solely in nursing homes. Studies reporting odds ratios, hazard ratios, or relative risks of death given dementia or similar exposure were extracted. Although our case definition was either the Diagnostic and Statistical Manual of Mental Disorders (DSM) or ICD definitions of dementia based on clinical ascertainment by a physician in a population‐representative study, we accepted a wider range of exposures including Alzheimer's disease (AD) and cognitive impairment and adjusted for differences in our modeling strategy to increase data volume (Table 1).

**TABLE 1:** Results of systematic review of 32 studies on all‐cause excess mortality with dementia

Characteristics of Data N (%)		60
Region name	East Asia	4 (6.7)
	Eastern sub‐Saharan Africa	2 (3.3)
	High‐income Asia Pacific	4 (6.7)
	High‐income North America	22 (36.7)
	North Africa and Middle East	1 (1.7)
	Tropical Latin America	1 (1.7)
	Western Europe	26 (43.3)
Exposure	Alzheimer's disease	11 (18.3)
	Cognitive impairment	10 (16.7)
	Dementia (total)	35 (58.3)
	Vascular dementia	4 (6.7)
Conducted in clinical setting	Clinical setting	10 (16.7)
	Community setting	50 (83.3)
Adjusted for education		32 (53.3)
Adjusted for cardiovascular disease^†^		33 (55.0)
Extensive cardiovascular disease adjustment^†^		15 (25.0)
Adjusted for smoking and alcohol		11 (18.3)
Adjusted for factors in causal pathway		13 (21.7)

^†^Base cardiovascular disease covariates included broad categories such as history of cardiovascular disease or stroke; extensive cardiovascular disease adjustment included more detailed information on specific conditions such as high blood pressure or hyperlipidemia.

Notes: All values represent the number of data points (% in parentheses).

### 2.3 Calculation and meta‐analysis of attributable risk

Using the data on relative risks extracted from each study and the GBD estimate of all‐cause mortality rate for the given study location and time period,^21^ we calculated attributable risk of all‐cause mortality. By multiplying the excess risk in the exposed (relative risk — 1) by the event rate in the unexposed (approximated using all‐cause mortality rates) we estimated the all‐cause mortality rate difference, or attributable risk, using Formula 2:

(2) $$\begin{array}{c} Attributable\;Risk\;=\left(Relative\;Risk-1\right)\;*All-Cause\;Mortality\;Rate \end{array}$$

We then conducted a Bayesian meta‐regression on the attributable risk data. The model includes covariates to predict between‐study heterogeneity and adds uncertainty from unexplained heterogeneity to coefficient estimates and predictions. Additionally, the model incorporates automatic outlier identification as part of the likelihood function, which identifies data with implausible combinations of means and variances, such that even if a data point is far from the mean, it may not be identified as an outlier if it has a high variance (additional details in supporting information).

The meta‐regression included covariates for the exposure used in each study (all dementia, AD, cognitive impairment), whether the study was conducted in a clinical sample, and five indicators for categories of variables commonly adjusted for in the component studies. These indicator variables described whether each component study of the meta‐regression adjusted for educational attainment, basic cardiovascular disease comorbidities (e.g., stroke and heart disease), more extensive cardiovascular disease factors (e.g., blood pressure and cholesterol), and smoking and alcohol consumption. Additionally, some studies adjusted for factors including activities of daily living, or residence in nursing homes, which may be on the causal pathway between dementia exposure and death, and these were grouped together into a dummy variable for the category of “over‐controlling.” Attributable risk was estimated from this model by generating predictions by age group for the mean population‐based study on dementia, which did not control for education, cardiovascular disease, or other factors.

### 2.4 Prevalence estimation and the calculation of excess deaths

We used all available data on the prevalence of dementia from cross‐sectional studies, cohort studies, and administrative claims databases for the estimation of dementia prevalence (additional details in supporting information; data sources are available at the Global Data Health Exchange [http://ghdx.healthdata.org/]). Using DisMod‐MR 2.1, the Bayesian meta‐regression tool primarily used in nonfatal modeling for GBD with settings of no remission and no incidence before age 40, we generated estimates for the prevalence of dementia by age, sex, year, and location (additional details in supporting information).^57^ We then calculated the total number of excess deaths due to dementia as the product of prevalence and attributable risk.

### 2.5 Calculation of the proportion of excess deaths attributable to dementia

Our estimate of the total number of excess deaths in those with dementia likely includes deaths that should be attributed to other conditions, such as cardiovascular diseases, that are more common in those with dementia due to shared risk factors such as high blood pressure or lower educational attainment.^58^ To calculate the number of deaths caused by dementia from the number of excess dementia deaths, we used mortality records linked to inpatient records, covering deaths from 2003 to 2017 in the Emilia Romagna region of Italy, and looked for markers of severe, end‐stage disease up to 1 year before death. A similar strategy of identifying cases of end‐stage disease using ICD codes has been implemented using Medicare data in the United States.^59^ To select these markers, for each ICD code in the data we calculated the difference in the proportion of individuals who died with dementia and had a record of such a code in the year before death and the proportion of individuals who died without dementia and had a record of the same code in the year before death. We defined dementia as any ICD‐9 code for dementia (290–290.4, 294.1–294.2, 294.8–294.9, 331.0–331.2), in either inpatient records or death records. We reviewed the 150 codes with the highest difference and selected codes that could indicate end‐stage disease, excluding codes for conditions such as cardiovascular disease or cancers that cannot plausibly be caused by dementia. Codes for decubitus ulcer, malnutrition, sepsis, pneumonia, urinary tract infections, falling from bed, senility, dehydration, sodium imbalance, muscular wasting, bronchitis, dysphagia, hip fracture, and bedridden status were used as indicators of severe disease (ICD code lists in supporting information).

To determine the proportion of excess deaths that could be ascribed to dementia as the underlying cause of death, we calculated the proportion of dementia deaths with markers of end‐stage disease in the year prior to death, above and beyond the occurrence of end‐stage disease markers in those who died without dementia. The subtraction of the proportions with end‐stage disease markers in those without dementia from the proportions in those with dementia we deemed to represent individuals who died with severe, end‐stage dementia.

To apply the estimates of the proportion who died with severe, end‐stage dementia out of those who died with dementia (died with severe disease/total deaths with dementia) to the total excess deaths we adjusted these proportions using the adjustment factor (relative risk/[relative risk – 1]) to calculate the proportion of individuals who died with end‐stage dementia out of excess dementia deaths with Formula 3:

(3) $$\begin{array}{ccc} & & \frac{Died\;with\;Severe\;Disease}{Excess\;Dementia\;Deaths}=\;\frac{Died\;with\;Severe\;Disease}{Total\;Deaths\;with\;Dementia}*\\ & & \frac{Relative\;Risk}{Relative\;Risk\;-\;1} \end{array}$$

Relative risks of mortality with dementia were estimated using a second meta‐regression model and the same studies identified through the literature review described in section 2.2.

### 2.6 Calculation of final results

We calculated the number of deaths due to dementia as the product of total excess dementia deaths and the proportion of those who died with severe disease out of excess dementia deaths. These results were combined with mortality estimates from all other causes of death and scaled to sum to the total estimated all‐cause mortality in a particular age, sex, year, and location combination. Age‐standardized rates were calculated using the world population standard for GBD.^60^ Regional and global estimates were calculated by aggregating the estimates from the most‐detailed locations. The Socio‐demographic Index (SDI), a composite measure of income, education, and fertility, was calculated as the geometric mean of normalized estimates of per capita income, the fertility rate in women under 25, and the average years of education in individuals 15 and older.^21^ Countries were divided into quintiles of SDI for analyses. Throughout all steps included in the analytic process, uncertainty in the estimates was propagated by sampling 1000 draws at each step. For example, the calculation of the product of two estimates involved conducting a draw‐wise multiplication of 1000 draws from the distribution of each estimate. We defined uncertainty intervals as the 25th and 975th values of the ordered draws.

## 3 RESULTS

We identified 34 studies containing 60 unique estimates (on a given, age, sex, and/or exposure category) on the excess mortality associated with dementia. These data covered 7 of the 21 world regions, most from high‐income North America and Western Europe (Table 1).

**FIGURE 2:**
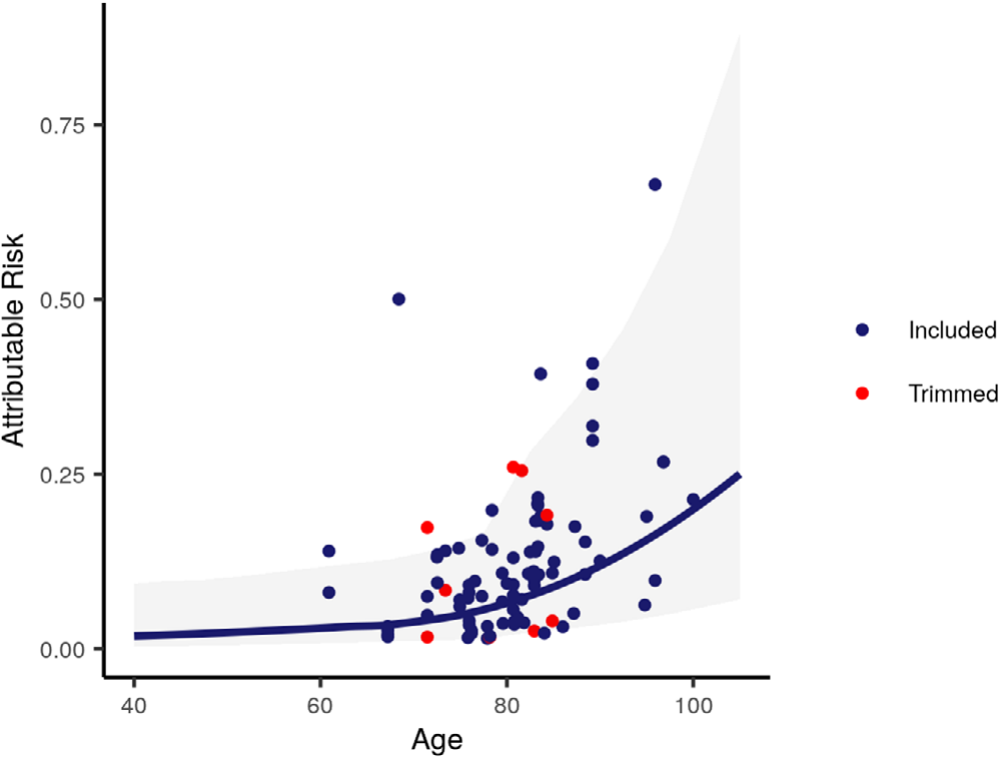
Attributable risk for all‐cause mortality due to dementia by age. Dots represent individual studies, and the line shows the results of the meta‐regression. Trimmed data points are those identified as outliers during the model‐fitting process; all other data points are included in the model. These estimates are applied across all years and geographies estimated in this study

Attributable risk increased with age, and this relationship became more pronounced after 70 years of age. In 40‐ to 44‐year‐olds, the attributable risk was 0.019 (0.003–0.087), and in the 95+ age group these estimates reached 0.169 (0.0472–0.574; Figure 2). This indicates that individuals over 95 with dementia experience a mortality rate that is 16,900 per 100,000 person‐years higher than those over 95 who do not have dementia.

**FIGURE 3:**
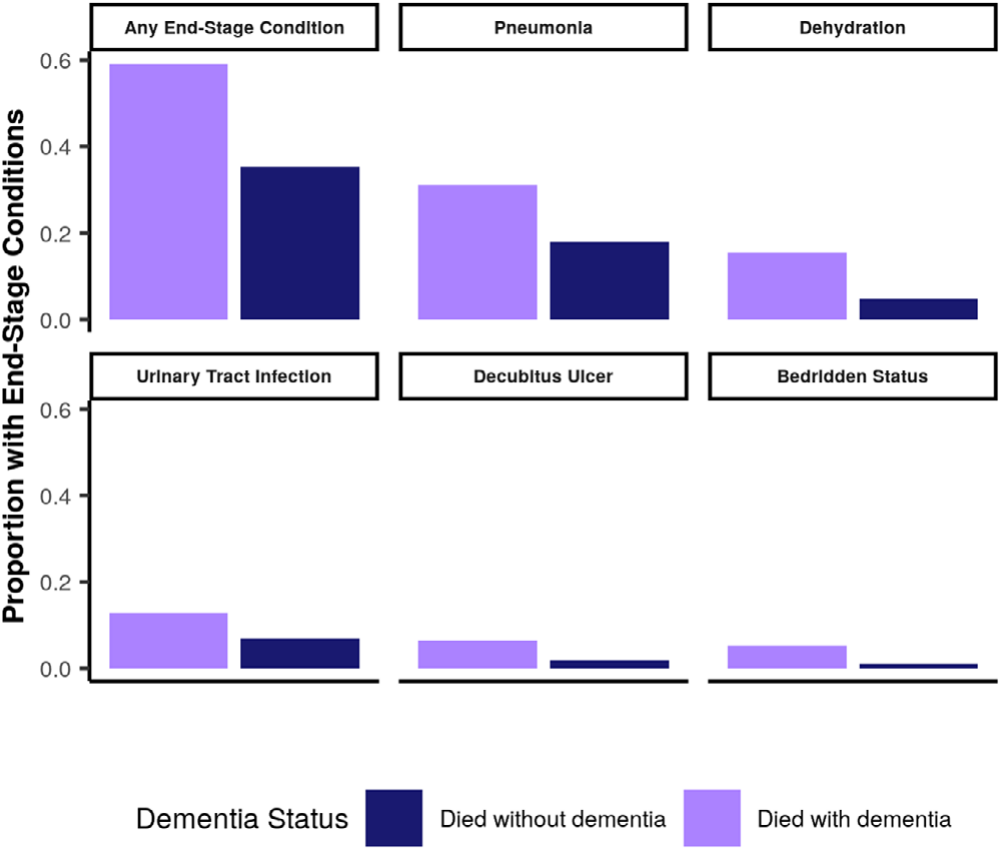
Proportion of individuals who died with end‐stage conditions in the last year before death for all conditions combined and the conditions with the top five excess proportions, shown separately for individuals who died with and without dementia. These estimates are derived from linked clinical and death records from 2003 to 2017 in the Emilia Romagna region of Italy. They are assumed to apply globally and over the time period considered in this study

Of the 14 markers of severe, end‐stage disease considered, the fraction of people who had pneumonia in the last year before death out of those who died with dementia was the highest (0.31). This proportion remained the highest (0.13) after subtracting the fraction of people without dementia who had the same end‐stage condition in the last year before death to calculate the excess proportion with pneumonia in those who died with dementia. The next end‐stage conditions with the largest absolute and excess proportions were dehydration (absolute: 0.15; excess: 0.11), urinary tract infections (0.12; 0.06), and decubitus ulcer (0.06; 0.05). The proportion of individuals who died with dementia and had any end‐stage disease marker in the year before death was 0.59, and the proportion of individuals with an end‐stage disease marker in the year before death who died without dementia was 0.35. Therefore, we calculated the proportion of people with dementia who died with severe, end‐stage dementia (the excess proportion) as 0.24 (Figure 3).

Overall, we estimated there were 1.62 million (0.41–4.21) deaths due to dementia globally in 2019. Far more deaths occurred in women (1.06 million [0.27–2.71]) than men (0.56 million [0.14–1.51]), giving a female‐to‐male ratio of 1.94 (1.76–2.11; Table 2). The female‐to‐male ratio for age‐standardized rates was 1.19 (1.10–1.26), suggesting that a portion of the sex difference was independent of the higher life expectancy in women. Between 1990 and 2019, there was a significant increase globally in age‐standardized mortality rates among males (5.1% [0.4–12.0]), but a non‐significant increase in age‐standardized mortality rates among females (3.0% [‐2.6–11.0]). Among males, the largest increase was observed for low SDI countries (9.6% [0.3–26.3]). Between 1990 and 2019, there were no significant changes globally in age‐standardized mortality rates for both sexes combined (3.0% [‐1.2–9.8]), but a large increase in all‐age mortality rates (100.1% [89.1–117.5]) due to population aging.

**TABLE 2:** Deaths in 2019 and percentage change of all‐age and age‐standardized mortality rates by location for dementia

	Male			Female		
Location	2019 counts	Percentage change in all‐age rates between 1990 and 2019 (95% UI)	Percentage change in age‐standardized rates between 1990 and 2019 (95% UI)	2019 counts	Percentage change in all‐age rates between 1990 and 2019 (95% UI)	Percentage change in age‐standardized rates between 1990 and 2019 (95% UI)
Global	559,228.5 (135,257.1 to 1505 721.2)	114.4 (102.1 to 132.5)	5.1 (0.4 to 12.0)	1,064,047.4 (274,500.8 to 2,705,358.7)	92.9 (80.4 to 112.3)	3.0 (−2.6 to 11.0)
Low SDI	25,788.3 (6018.1 to 73,781.0)	33.9 (21.5 to 53.8)	9.6 (0.3 to 26.3)	36,307.3 (9062.9 to 97,442.0)	47.3 (29.1 to 71.9)	10.6 (−3.0 to 28.8)
Low‐middle SDI	75,886.8 (17,739.8 to 212,133.3)	109.8 (90.1 to 140.2)	9.1 (0.3 to 24.0)	109,380.8 (27,179.5 to 289,174.0)	128.4 (102.0 to 163.9)	5.8 (−6.0 to 22.0)
Middle SDI	145,060.8 (34,246.2 to 397,115.0)	140.7 (117.6 to 173.1)	3.4 (−4.5 to 15.9)	242,455.4 (58,645.8 to 626,326.7)	127.4 (103.4 to 169.8)	−0.3 (−9.7 to 18.1)
High‐middle SDI	135,182.4 (31,908.3 to 368,661.4)	136.0 (117.9 to 159.0)	1.7 (−4.1 to 8.9)	289,148.3 (73,256.3 to 753,758.8)	120.4 (102.3 to 147.4)	1.7 (−4.8 to 11.8)
High SDI	176,987.3 (44,259.2 to 469,442.4)	140.8 (126.4 to 161.1)	5.8 (1.4 to 11.7)	386,228.6 (103,824.8 to 924,598.2)	102.9 (86.1 to 124.7)	3.6 (−2.3 to 11.8)
Central Asia	2879.9 (675.3 to 8160.8)	−7.4 (−15.7 to 4.8)	9.5 (2.1 to 25.6)	6067.6 (1472.1 to 16,430.4)	−11.6 (−18.2 to −4.6)	8.3 (0.9 to 16.7)
Armenia	315.3 (74.0 to 858.6)	188.0 (151.7 to 233.2)	16.8 (3.0 to 35.0)	557.7 (134.0 to 1497.2)	166.2 (132.6 to 207.5)	13.7 (−0.1 to 30.2)
Azerbaijan	352.0 (79.9 to 1,037.9)	−5.7 (−24.9 to 32.6)	12.4 (−6.8 to 82.5)	588.7 (126.7 to 1698.2)	−32.9 (−49.2 to 3.2)	18.1 (−5.3 to 91.7)
Georgia	483.6 (113.9 to 1325.8)	209.1 (164.8 to 265.2)	5.7 (−6.4 to 20.7)	1236.9 (307.6 to 3240.4)	204.6 (157.8 to 271.8)	1.5 (−10.9 to 18.5)
Kazakhstan	655.8 (151.2 to 1902.7)	25.1 (8.2 to 48.0)	12.3 (−0.7 to 37.0)	1663.6 (387.9 to 4673.7)	8.7 (−3.0 to 21.9)	9.4 (−1.9 to 21.3)
Kyrgyzstan	242.8 (58.3 to 667.0)	11.4 (−0.1 to 24.1)	10.1 (−0.6 to 21.3)	480.0 (113.7 to 1 362.3)	−20.8 (−30.8 to −11.5)	11.5 (−1.8 to 24.1)
Mongolia	77.9 (17.9 to 225.7)	8.7 (−11.1 to 31.4)	−1.5 (−15.2 to 15.2)	146.7 (34.1 to 420.4)	−2.0 (−20.3 to 19.3)	−1.6 (−18.5 to 17.6)
Tajikistan	167.9 (38.8 to 481.3)	−58.7 (−67.3 to −50.8)	12.8 (−5.6 to 31.1)	277.7 (66.5 to 754.4)	−56.3 (−64.3 to −48.3)	14.3 (−5.9 to 33.7)
Turkmenistan	179.7 (42.9 to 499.0)	46.3 (23.6 to 77.3)	0.2 (−14.2 to 20.3)	402.5 (100.2 to 1058.6)	68.1 (43.4 to 102.7)	−1.7 (−15.7 to 16.6)
Uzbekistan	405.0 (94.6 to 1151.2)	−63.5 (−71.3 to −55.7)	5.2 (−6.6 to 17.2)	713.8 (168.5 to 2057.7)	−63.7 (−71.0 to −57.1)	11.9 (−2.0 to 22.3)
Central Europe	15,986.2 (3813.1 to 42,672.8)	122.6 (100.3 to 149.0)	−1.2 (−9.1 to 7.9)	35,119.8 (8472.2 to 91,059.3)	132.1 (110.2 to 161.7)	−1.9 (−9.8 to 7.9)
Albania	344.3 (78.1 to 913.5)	303.2 (222.9 to 404.0)	−2.0 (−19.7 to 21.6)	572.5 (138.4 to 1477.1)	167.1 (114.4 to 232.1)	−3.1 (−21.6 to 20.2)
Bosnia and Herzegovina	390.2 (90.1 to 1 064.5)	200.4 (150.9 to 256.7)	−3.1 (−18.5 to 13.7)	689.5 (160.4 to 1871.6)	184.1 (136.3 to 236.6)	−4.7 (−20.7 to 12.2)
Bulgaria	1057.3 (239.8 to 3004.6)	135.0 (95.1 to 178.8)	1.6 (−12.0 to 17.8)	2088.7 (485.9 to 5832.3)	188.9 (142.0 to 252.1)	−1.6 (−15.4 to 15.3)
Croatia	660.9 (151.8 to 1832.8)	151.7 (112.5 to 206.0)	−0.2 (−14.7 to 18.2)	1504.6 (360.7 to 3891.0)	144.3 (105.2 to 194.5)	1.7 (−13.9 to 21.1)
Czech Republic	1609.2 (375.9 to 4202.4)	123.4 (91.2 to 165.5)	1.5 (−11.3 to 18.5)	3,511.6 (865.5 to 9000.4)	97.6 (69.7 to 139.3)	4.2 (−9.6 to 24.4)
Hungary	1 335.7 (306.5 to 3611.5)	98.9 (73.2 to 132.9)	−0.8 (−12.0 to 13.7)	3 514.0 (840.8 to 8909.8)	125.2 (94.6 to 167.9)	3.3 (−9.5 to 21.3)
Montenegro	67.2 (15.3 to 182.9)	63.3 (40.6 to 90.5)	−0.4 (−13.1 to 18.4)	117.4 (27.4 to 309.8)	60.3 (38.0 to 112.7)	3.9 (−9.7 to 41.7)
North Macedonia	169.4 (38.3 to 474.1)	56.9 (30.7 to 89.3)	6.8 (−9.3 to 33.0)	267.2 (59.6 to 741.7)	66.2 (36.3 to 105.9)	9.6 (−8.4 to 41.4)
Poland	5430.2 (1260.0 to 14,735.3)	117.5 (84.0 to 161.0)	−5.5 (−18.1 to 11.0)	13,038.4 (3096.3 to 35,654.9)	116.3 (83.8 to 157.8)	−6.5 (−20.0 to 10.7)
Romania	2 973.2 (718.3 to 7868.4)	158.9 (121.8 to 202.9)	−1.0 (−12.4 to 13.0)	5901.3 (1457.2 to 15,674.1)	208.9 (162.3 to 270.1)	−0.6 (−14.0 to 15.7)
Serbia	1 010.4 (224.3 to 2771.2)	65.4 (37.1 to 108.1)	3.7 (−10.9 to 28.7)	1,557.0 (349.5 to 4456.0)	57.6 (28.5 to 101.5)	−1.6 (−17.2 to 23.5)
Slovakia	562.8 (129.2 to 1490.3)	57.4 (31.6 to 87.8)	−2.4 (−17.4 to 14.5)	1396.7 (329.5 to 3535.6)	83.1 (53.9 to 122.5)	−2.1 (−17.7 to 17.2)
Slovenia	375.5 (90.3 to 991.7)	140.3 (87.9 to 205.9)	4.1 (−16.8 to 30.7)	961.1 (249.1 to 2423.8)	179.3 (112.8 to 274.5)	0.8 (−22.0 to 31.0)
Eastern Europe	18,376.8 (4327.3 to 49,475.5)	142.5 (118.0 to 177.5)	6.1 (−2.9 to 18.6)	54,705.3 (13,131.1 to 149,095.2)	94.8 (75.7 to 118.7)	6.5 (−2.6 to 17.8)
Belarus	844.2 (198.4 to 2243.7)	59.0 (34.5 to 90.8)	4.6 (−10.6 to 24.4)	2841.3 (695.2 to 7511.8)	81.3 (52.9 to 119.6)	4.4 (−11.6 to 26.1)
Estonia	182.9 (44.1 to 489.5)	194.1 (145.4 to 256.5)	14.7 (−2.3 to 36.7)	573.8 (145.1 to 1510.1)	169.3 (122.3 to 224.8)	13.5 (−5.0 to 35.2)
Latvia	243.5 (58.4 to 670.6)	124.8 (90.1 to 168.0)	8.6 (−5.9 to 27.5)	787.5 (185.6 to 2045.9)	136.6 (95.9 to 189.3)	7.3 (−11.1 to 29.9)
Lithuania	382.4 (92.1 to 1029.4)	94.1 (67.4 to 121.8)	5.4 (−7.7 to 19.1)	1159.7 (286.4 to 3061.9)	153.2 (119.0 to 195.0)	4.4 (−9.5 to 20.1)
Moldova	377.9 (89.8 to 1046.9)	167.3 (137.7 to 204.3)	−1.8 (−11.0 to 9.1)	881.3 (217.8 to 2361.8)	184.7 (151.7 to 232.3)	−0.4 (−9.9 to 13.3)
Russia	12,039.2 (2888.0 to 32,033.2)	178.4 (140.4 to 234.6)	4.7 (−7.3 to 20.0)	36,398.0 (8759.0 to 97,034.3)	93.9 (68.9 to 123.6)	4.1 (−7.8 to 18.3)
Ukraine	4306.7 (994.6 to 12,348.2)	107.6 (80.4 to 140.8)	10.4 (−1.5 to 25.1)	12,063.6 (2805.3 to 32,884.6)	91.0 (61.8 to 126.2)	14.3 (−2.3 to 33.6)
Australasia	4561.0 (1113.8 to 12,133.4)	155.7 (133.8 to 189.7)	0.3 (−5.8 to 9.6)	8745.6 (2319.4 to 21,727.2)	90.0 (75.7 to 112.2)	−0.5 (−6.2 to 8.5)
Australia	3841.2 (935.1 to 10,162.1)	157.6 (133.3 to 194.3)	0.6 (−6.2 to 11.8)	7399.1 (1971.3 to 18,237.9)	91.1 (75.6 to 115.3)	−0.6 (−7.1 to 9.8)
New Zealand	719.9 (176.3 to 1965.0)	147.7 (126.9 to 176.5)	−0.8 (−7.7 to 8.5)	1346.6 (351.7 to 3385.6)	84.7 (70.9 to 105.6)	−0.2 (−6.3 to 8.7)
High‐income Asia Pacific	56,677.3 (14,611.8 to 148,035.7)	425.2 (375.0 to 506.3)	28.5 (19.4 to 41.0)	128,148.5 (37,175.4 to 293,499.2)	436.5 (357.5 to 548.1)	21.8 (8.1 to 38.8)
Brunei	7.3 (1.7 to 19.5)	100.5 (69.0 to 137.7)	9.2 (−3.4 to 24.4)	16.3 (3.8 to 45.4)	83.5 (63.7 to 108.8)	9.5 (−2.3 to 23.2)
Japan	50,832.5 (13,165.2 to 132,325.7)	456.7 (401.3 to 545.3)	32.2 (22.6 to 45.6)	114,041.4 (33,669.4 to 259,294.0)	475.1 (387.0 to 602.7)	25.8 (10.5 to 43.8)
South Korea	5329.9 (1302.9 to 14,658.1)	412.4 (344.2 to 503.2)	−5.2 (−17.0 to 9.5)	13,121.0 (3431.0 to 32,727.1)	337.5 (280.1 to 427.8)	−5.9 (−16.5 to 10.4)
Singapore	507.7 (124.3 to 1 325.9)	197.3 (169.7 to 240.2)	−0.5 (−7.7 to 10.9)	969.8 (261.3 to 2 345.0)	175.6 (147.6 to 213.6)	−1.6 (−10.3 to 10.9)
High‐income North America	51,662.1 (12,640.0 to 134,630.7)	78.5 (64.1 to 98.8)	−2.1 (−10.2 to 8.0)	109,320.0 (28,623.1 to 263,132.9)	47.7 (35.5 to 64.8)	−0.9 (−8.5 to 9.1)
Canada	5579.0 (1372.3 to 14,551.0)	121.5 (101.1 to 147.8)	−5.4 (−14.1 to 5.3)	11,472.9 (3009.7 to 28,285.5)	86.8 (71.0 to 108.1)	−6.1 (−13.4 to 3.4)
Greenland	3.5 (0.8 to 9.5)	214.3 (167.7 to 265.7)	0.5 (−12.5 to 16.5)	5.5 (1.4 to 14.5)	150.3 (111.0 to 208.6)	0.2 (−14.8 to 21.3)
USA	46,078.8 (11,288.7 to 120,166.5)	74.5 (59.8 to 94.8)	−1.8 (−10.2 to 8.5)	97,839.9 (25,611.1 to 235,757.6)	44.3 (32.2 to 61.7)	−0.4 (−8.3 to 9.9)
Southern Latin America	5893.1 (1420.9 to 16,373.0)	86.3 (74.6 to 102.6)	1.7 (−3.7 to 8.6)	12,453.3 (3063.9 to 32,452.5)	96.4 (83.1 to 112.5)	0.5 (−4.8 to 6.4)
Argentina	3694.7 (875.9 to 10,300.6)	69.4 (56.0 to 86.1)	2.0 (−5.6 to 11.5)	8349.9 (2038.8 to 21,613.4)	80.9 (66.4 to 97.1)	1.4 (−5.5 to 8.7)
Chile	1761.1 (429.1 to 4751.0)	149.2 (128.7 to 178.3)	2.7 (−4.4 to 13.4)	3042.8 (786.9 to 7969.3)	173.4 (150.7 to 211.1)	0.2 (−7.1 to 10.3)
Uruguay	437.0 (106.3 to 1210.8)	74.1 (59.4 to 92.2)	−3.0 (−9.8 to 5.4)	1 059.8 (268.5 to 2712.3)	90.2 (76.5 to 108.1)	−1.1 (−7.6 to 6.3)
Western Europe	82,115.7 (19,923.2 to 224,410.0)	126.6 (114.1 to 144.8)	−1.4 (−4.9 to 3.7)	182,650.0 (47,390.6 to 451,716.7)	82.1 (71.4 to 97.8)	−3.8 (−7.5 to 2.0)
Andorra	12.8 (3.2 to 33.6)	273.6 (180.5 to 404.8)	−5.0 (−23.6 to 15.1)	23.6 (6.4 to 60.4)	355.3 (218.5 to 541.6)	−3.8 (−28.9 to 24.8)
Austria	1391.5 (332.7 to 3783.9)	113.0 (96.3 to 137.0)	−1.4 (−8.1 to 7.4)	3410.3 (867.8 to 8730.4)	71.8 (56.5 to 93.9)	−2.0 (−8.2 to 6.8)
Belgium	1950.6 (469.2 to 5385.4)	109.6 (90.7 to 132.6)	−4.0 (−11.5 to 4.7)	4664.0 (1196.1 to 11,653.6)	70.4 (54.1 to 91.4)	−6.5 (−13.7 to 2.6)
Cyprus	114.2 (27.0 to 330.4)	69.5 (46.9 to 96.1)	−12.6 (−22.5 to 0.4)	209.9 (51.1 to 570.8)	120.0 (84.8 to 164.6)	−9.6 (−19.8 to 3.2)
Denmark	850.3 (203.5 to 2331.7)	54.9 (38.1 to 72.9)	−0.5 (−11.2 to 11.0)	1852.5 (476.1 to 4761.0)	37.6 (20.2 to 57.1)	0.6 (−11.3 to 13.2)
Finland	947.1 (227.0 to 2553.2)	167.3 (145.3 to 196.9)	−5.8 (−12.4 to 3.1)	2333.1 (604.5 to 5811.3)	101.7 (80.8 to 130.3)	−6.0 (−15.0 to 3.8)
France	13,214.6 (3219.8 to 34,550.9)	145.1 (119.8 to 179.4)	2.9 (−5.3 to 15.0)	32,793.3 (8754.9 to 77,733.2)	102.0 (82.4 to 133.9)	3.5 (−5.7 to 18.3)
Germany	14,694.6 (3500.3 to 40,974.5)	97.5 (77.0 to 122.4)	−14.8 (−23.6 to −4.9)	34,861.9 (8873.8 to 88,539.8)	45.6 (26.6 to 65.5)	−17.5 (−28.1 to −7.4)
Greece	2694.1 (642.9 to 7496.8)	198.4 (167.4 to 248.5)	1.1 (−6.0 to 13.3)	4302.2 (1073.8 to 11,309.6)	166.8 (142.9 to 196.8)	−0.9 (−7.1 to 7.1)
Iceland	49.8 (12.2 to 130.5)	73.3 (58.2 to 91.0)	0.9 (−7.9 to 11.0)	100.8 (26.9 to 240.5)	70.2 (51.9 to 96.3)	−3.8 (−13.4 to 9.7)
Ireland	546.3 (127.8 to 1499.1)	98.2 (81.3 to 122.8)	0.1 (−7.3 to 10.9)	1110.7 (282.0 to 2781.9)	74.5 (58.6 to 97.6)	−0.3 (−7.3 to 10.2)
Israel	969.7 (236.1 to 2587.2)	74.2 (57.8 to 98.2)	−0.7 (−7.2 to 7.5)	1807.1 (474.3 to 4561.1)	123.6 (100.4 to 159.8)	−1.6 (−7.9 to 7.8)
Italy	15,854.3 (3907.5 to 43,081.1)	246.8 (223.5 to 279.0)	19.5 (10.2 to 32.1)	32,908.4 (8694.0 to 83,572.0)	206.1 (184.8 to 236.1)	16.4 (7.7 to 29.3)
Luxembourg	70.4 (17.1 to 191.1)	73.2 (51.1 to 103.0)	−3.6 (−14.2 to 11.6)	171.1 (45.1 to 426.7)	55.5 (35.1 to 86.6)	−5.7 (−16.4 to 9.9)
Malta	67.9 (16.1 to 185.3)	174.9 (150.1 to 211.7)	0.7 (−7.5 to 13.3)	151.8 (38.8 to 374.0)	191.7 (162.3 to 236.9)	−0.7 (−10.3 to 12.2)
Monaco	9.2 (2.2 to 24.4)	60.1 (36.7 to 91.9)	6.5 (−8.6 to 23.6)	19.1 (4.9 to 48.1)	29.9 (5.8 to 60.1)	5.8 (−15.4 to 28.8)
Netherlands	2826.1 (689.6 to 7493.6)	98.4 (85.4 to 116.1)	−4.1 (−10.3 to 4.4)	5888.5 (1523.4 to 14,301.4)	52.7 (42.4 to 68.8)	−7.3 (−13.0 to 0.6)
Norway	800.4 (193.2 to 2149.6)	29.4 (21.5 to 40.0)	−7.0 (−11.3 to −1.2)	1832.9 (474.6 to 4476.3)	21.2 (12.8 to 32.9)	−6.3 (−10.6 to 1.7)
Portugal	2003.9 (467.5 to 5470.8)	229.3 (196.0 to 275.1)	3.9 (−3.8 to 14.5)	4597.9 (1161.5 to 11,636.9)	200.8 (172.4 to 250.2)	1.9 (−5.6 to 14.2)
San Marino	6.4 (1.5 to 17.1)	138.2 (74.1 to 209.4)	−2.5 (−27.5 to 24.0)	13.2 (3.3 to 33.7)	114.1 (57.0 to 187.5)	−2.5 (−29.2 to 31.9)
Spain	8297.1 (2005.4 to 22,012.2)	100.6 (75.7 to 128.4)	−16.8 (−25.1 to −7.7)	20,910.6 (5456.7 to 50,985.5)	110.0 (88.9 to 140.6)	−14.6 (−20.6 to −5.4)
Sweden	1882.2 (451.4 to 5170.4)	48.3 (34.7 to 65.3)	−8.3 (−15.3 to 0.5)	3755.2 (953.2 to 9502.8)	19.6 (8.7 to 32.4)	−13.2 (−20.1 to −5.2)
Switzerland	1560.4 (386.5 to 4188.2)	98.4 (81.7 to 123.3)	3.2 (−3.7 to 13.2)	3577.9 (945.9 to 8761.0)	68.3 (53.6 to 89.2)	4.5 (−2.7 to 14.2)
UK	11,230.0 (2706.1 to 30 400.6)	103.2 (90.9 to 120.9)	3.6 (−0.3 to 9.7)	21,194.9 (5393.2 to 53,699.3)	33.9 (26.2 to 46.8)	−1.5 (−5.4 to 6.0)
Andean Latin America	4943.7 (1190.8 to 12,671.0)	98.4 (64.6 to 136.8)	2.9 (−14.1 to 22.6)	6231.1 (1516.5 to 15,850.1)	117.7 (84.7 to 164.0)	−0.4 (−14.8 to 20.5)
Bolivia	611.2 (137.5 to 1635.8)	96.3 (57.8 to 147.1)	5.3 (−14.9 to 31.7)	781.4 (180.3 to 2072.7)	75.9 (47.6 to 112.3)	7.5 (−9.9 to 30.2)
Ecuador	1169.1 (282.2 to 3327.5)	71.9 (41.8 to 105.9)	6.4 (−12.2 to 26.5)	1469.2 (350.9 to 4015.4)	104.5 (70.5 to 145.8)	−1.0 (−16.2 to 17.8)
Peru	3163.4 (754.4 to 8193.0)	114.8 (69.5 to 174.5)	1.3 (−20.2 to 31.2)	3980.6 (994.2 to 10,023.5)	137.5 (89.6 to 208.6)	−2.0 (−21.5 to 27.4)
Caribbean	4597.5 (1153.7 to 11,746.2)	87.0 (66.1 to 111.8)	1.1 (−9.7 to 13.2)	6396.5 (1655.1 to 15,821.9)	112.1 (89.1 to 143.4)	0.5 (−9.5 to 12.5)
Antigua and Barbuda	6.4 (1.5 to 17.3)	21.3 (6.0 to 38.2)	−0.5 (−12.1 to 12.6)	10.0 (2.4 to 27.2)	−20.4 (−29.5 to −11.9)	4.7 (−6.4 to 15.6)
The Bahamas	23.0 (5.4 to 63.5)	119.1 (85.7 to 160.3)	−0.3 (−13.1 to 15.9)	37.9 (9.6 to 101.5)	73.8 (50.3 to 104.2)	−0.9 (−14.3 to 15.2)
Barbados	39.6 (9.6 to 106.8)	65.9 (45.2 to 91.1)	3.0 (−8.8 to 16.6)	60.9 (14.6 to 159.9)	37.5 (20.9 to 57.0)	7.2 (−5.3 to 21.4)
Belize	22.5 (5.4 to 59.1)	34.2 (18.2 to 54.5)	6.5 (−6.0 to 22.2)	25.1 (6.3 to 64.1)	11.4 (−1.1 to 27.0)	0.3 (−10.8 to 14.3)
Bermuda	10.8 (2.6 to 28.9)	193.4 (160.1 to 237.9)	−2.6 (−12.8 to 12.0)	21.0 (5.4 to 53.0)	185.0 (146.5 to 246.3)	−0.8 (−13.6 to 20.5)
Cuba	1940.9 (478.3 to 5167.7)	123.3 (88.9 to 163.6)	1.5 (−13.1 to 18.5)	2562.3 (659.1 to 6742.3)	166.0 (125.6 to 216.7)	−0.9 (−15.8 to 16.0)
Dominica	7.2 (1.7 to 19.6)	50.9 (27.8 to 79.4)	−1.4 (−16.1 to 16.9)	12.1 (2.9 to 32.2)	35.4 (16.6 to 58.7)	0.5 (−12.5 to 17.2)
Dominican Republic	692.0 (161.6 to 1874.1)	85.7 (49.4 to 128.2)	−2.9 (−20.5 to 17.3)	937.1 (225.1 to 2476.2)	134.1 (94.9 to 185.2)	−1.3 (−17.4 to 19.8)
Grenada	4.7 (1.1 to 13.1)	−36.9 (−45.4 to −26.6)	5.2 (−4.0 to 16.8)	11.5 (2.8 to 30.4)	−15.9 (−23.7 to −6.9)	5.7 (−3.7 to 16.9)
Guyana	32.6 (7.6 to 88.7)	70.7 (40.1 to 108.7)	4.6 (−12.5 to 25.0)	46.6 (11.2 to 125.6)	81.4 (51.4 to 117.9)	1.1 (−14.9 to 19.7)
Haiti	370.5 (82.2 to 1 017.0)	52.6 (22.5 to 89.8)	3.9 (−14.4 to 25.8)	396.1 (87.6 to 1 151.9)	30.3 (−1.0 to 77.1)	17.0 (−11.6 to 58.1)
Jamaica	308.2 (76.1 to 781.0)	74.1 (47.6 to 107.3)	1.6 (−13.9 to 20.0)	471.9 (119.4 to 1 181.8)	83.1 (55.6 to 117.5)	−0.3 (−14.9 to 18.4)
Puerto Rico	768.9 (190.9 to 1968.7)	189.6 (140.4 to 257.5)	−2.4 (−18.2 to 19.6)	1257.2 (322.9 to 3170.4)	235.9 (180.6 to 313.0)	−2.1 (−17.6 to 19.4)
Saint Kitts and Nevis	2.7 (0.6 to 7.5)	−12.3 (−21.7 to 0.0)	−3.5 (−13.1 to 9.5)	5.5 (1.3 to 14.8)	10.7 (−4.0 to 33.1)	−3.7 (−14.3 to 12.7)
Saint Lucia	15.6 (3.8 to 42.2)	161.4 (128.2 to 202.6)	1.2 (−9.6 to 15.6)	25.3 (6.2 to 66.4)	139.3 (110.9 to 179.4)	4.0 (−7.3 to 19.7)
Saint Vincent and the Grenadines	9.8 (2.3 to 26.6)	122.1 (97.4 to 152.7)	−5.6 (−14.8 to 5.3)	12.1 (2.9 to 32.1)	53.9 (39.5 to 71.2)	−6.3 (−15.1 to 3.7)
Suriname	41.8 (9.9 to 109.9)	58.7 (36.8 to 84.3)	2.1 (−11.9 to 18.6)	67.3 (16.8 to 171.4)	49.2 (30.1 to 72.5)	3.3 (−10.2 to 19.5)
Trinidad and Tobago	133.9 (31.3 to 364.5)	163.0 (111.4 to 233.4)	−2.5 (−19.1 to 18.9)	201.2 (49.5 to 517.9)	163.7 (117.2 to 234.1)	−0.3 (−17.2 to 23.5)
Virgin Islands	10.7 (2.5 to 28.8)	171.7 (131.8 to 219.9)	−5.4 (−17.3 to 7.9)	18.7 (4.6 to 49.2)	176.1 (137.5 to 222.3)	−4.9 (−17.4 to 9.9)
Central Latin America	22,046.6 (5370.3 to 55 473.9)	142.1 (115.2 to 175.0)	3.2 (−7.6 to 16.1)	29,344.4 (7535.4 to 75 028.8)	169.9 (140.5 to 211.1)	0.2 (−9.6 to 14.6)
Colombia	5678.3 (1422.9 to 14,469.7)	227.2 (167.3 to 302.3)	0.8 (−17.0 to 24.2)	7994.1 (2047.8 to 19, 681.9)	263.4 (204.2 to 350.4)	−1.0 (−17.0 to 21.7)
Costa Rica	541.3 (134.3 to 1361.9)	125.9 (87.1 to 177.8)	3.5 (−14.1 to 25.9)	753.9 (198.2 to 1821.8)	155.6 (113.6 to 211.7)	1.8 (−14.1 to 24.0)
El Salvador	666.4 (161.4 to 1716.1)	133.8 (91.5 to 180.5)	6.2 (−13.2 to 27.2)	1028.7 (261.4 to 2522.0)	144.8 (99.2 to 199.6)	4.9 (−15.1 to 29.0)
Guatemala	801.8 (188.5 to 2236.1)	68.0 (34.9 to 109.0)	1.6 (−15.0 to 20.8)	1139.2 (272.9 to 3013.9)	149.7 (105.7 to 207.1)	−2.9 (−17.4 to 16.2)
Honduras	512.8 (120.9 to 1411.4)	52.9 (25.9 to 112.0)	14.4 (−5.4 to 59.2)	545.2 (126.9 to 1509.6)	50.3 (20.4 to 102.6)	16.2 (−6.1 to 55.2)
Mexico	10,665.9 (2510.6 to 29,581.7)	133.6 (100.6 to 173.2)	5.5 (−8.0 to 22.2)	12,991.0 (3178.0 to 35,284.5)	157.3 (123.2 to 203.1)	−1.2 (−13.4 to 14.4)
Nicaragua	283.4 (69.6 to 802.6)	85.6 (55.8 to 111.3)	−0.3 (−15.1 to 12.6)	422.8 (104.4 to 1103.7)	65.4 (40.9 to 86.6)	13.9 (−2.3 to 28.2)
Panama	449.8 (111.1 to 1145.0)	92.8 (57.6 to 133.2)	3.3 (−16.0 to 25.0)	604.0 (158.8 to 1467.4)	120.6 (84.6 to 168.4)	2.4 (−14.2 to 25.4)
Venezuela	2 447.1 (585.7 to 6349.0)	143.3 (100.4 to 194.7)	0.3 (−17.4 to 20.8)	3 865.7 (977.6 to 9437.2)	170.7 (124.2 to 238.8)	−1.9 (−18.2 to 20.9)
Tropical Latin America	21,334.4 (5340.7 to 55,586.3)	170.3 (153.0 to 199.4)	−1.0 (−5.8 to 5.9)	34,520.5 (8799.4 to 88,150.5)	181.8 (162.6 to 210.1)	−6.0 (−10.3 to 0.8)
Brazil	20 831.7 (5224.0 to 54,506.7)	174.1 (156.0 to 203.4)	−1.4 (−6.4 to 5.8)	33,762.4 (8603.2 to 86,309.3)	185.5 (165.7 to 214.7)	−6.4 (−10.8 to 0.3)
Paraguay	502.8 (121.9 to 1253.3)	65.8 (35.2 to 103.6)	4.9 (−14.8 to 28.3)	758.1 (189.9 to 1902.3)	75.7 (42.7 to 117.5)	4.0 (−15.6 to 28.0)
North Africa and Middle East	33,001.5 (8027.2 to 86,655.5)	73.4 (59.5 to 103.6)	−3.5 (−10.1 to 11.6)	37,481.3 (9380.5 to 98,165.8)	59.3 (45.8 to 101.1)	−0.6 (−9.0 to 24.5)
Afghanistan	875.2 (206.7 to 2374.4)	−56.8 (−64.7 to −48.9)	−3.4 (−19.2 to 11.9)	899.7 (214.1 to 2458.6)	−47.7 (−57.3 to −36.9)	−2.8 (−18.5 to 14.5)
Algeria	2658.5 (643.0 to 7417.4)	141.2 (91.4 to 205.3)	−7.3 (−23.3 to 12.9)	2550.8 (602.0 to 7338.0)	129.7 (82.6 to 218.0)	−4.6 (−19.2 to 20.8)
Bahrain	36.0 (8.7 to 100.1)	68.2 (35.6 to 114.0)	−6.7 (−20.9 to 12.3)	37.7 (9.1 to 102.0)	73.7 (43.8 to 110.4)	−1.3 (−16.4 to 17.0)
Egypt	4204.1 (944.3 to 11,324.0)	51.7 (23.9 to 89.4)	−2.4 (−18.8 to 20.6)	2713.9 (609.7 to 7663.6)	−11.3 (−29.4 to 28.4)	3.2 (−15.7 to 46.7)
Iran	6894.6 (1 707.3 to 18 560.6)	265.1 (220.5 to 357.9)	−4.4 (−11.7 to 20.0)	7015.5 (1755.2 to 18,668.1)	209.0 (175.5 to 355.0)	3.5 (−6.1 to 53.2)
Iraq	1372.4 (319.3 to 3659.0)	−3.1 (−19.6 to 15.1)	−1.6 (−16.4 to 15.5)	1886.2 (455.0 to 4862.0)	−0.2 (−17.1 to 28.1)	0.1 (−16.1 to 28.1)
Jordan	388.2 (95.3 to 1057.6)	56.0 (24.1 to 96.0)	−7.8 (−25.1 to 14.2)	378.8 (89.7 to 1023.6)	41.3 (14.5 to 93.9)	−6.3 (−23.3 to 29.8)
Kuwait	244.3 (60.8 to 635.1)	195.6 (147.0 to 250.7)	2.0 (−13.2 to 18.7)	180.8 (47.8 to 441.7)	51.6 (31.3 to 80.9)	−3.7 (−16.4 to 13.9)
Lebanon	487.8 (115.9 to 1331.5)	118.2 (76.4 to 228.6)	−1.6 (−19.5 to 48.7)	767.5 (184.9 to 2079.4)	117.4 (78.6 to 237.5)	−3.6 (−20.7 to 48.4)
Libya	500.0 (126.9 to 1328.2)	60.5 (28.3 to 105.1)	−4.8 (−23.7 to 22.3)	593.9 (149.9 to 1501.2)	68.6 (36.2 to 116.9)	−3.0 (−21.6 to 24.5)
Morocco	2388.6 (549.1 to 6468.5)	100.5 (60.0 to 142.7)	0.5 (−17.0 to 21.2)	2556.2 (585.6 to 6989.0)	71.6 (46.6 to 99.5)	0.6 (−13.7 to 15.3)
Oman	72.2 (16.6 to 200.0)	−0.3 (−20.1 to 31.4)	−4.6 (−20.9 to 30.8)	90.6 (20.9 to 255.9)	−4.4 (−24.1 to 50.9)	−2.0 (−20.7 to 57.6)
Palestine	118.2 (27.1 to 336.8)	−24.3 (−37.3 to −4.8)	−5.0 (−20.5 to 26.0)	207.7 (48.7 to 585.0)	−12.3 (−28.0 to 9.0)	−6.2 (−22.3 to 17.0)
Qatar	29.7 (6.9 to 84.7)	17.5 (−16.0 to 62.3)	4.5 (−17.6 to 31.9)	11.4 (2.7 to 33.5)	−49.0 (−62.7 to −30.1)	13.3 (−7.4 to 40.0)
Saudi Arabia	899.5 (215.6 to 2384.9)	−2.3 (−19.3 to 34.6)	−3.2 (−18.8 to 29.7)	808.0 (195.3 to 2181.4)	−11.1 (−31.5 to 37.8)	−0.4 (−22.7 to 55.3)
Sudan	1417.2 (322.1 to 3962.5)	22.0 (3.3 to 47.3)	−5.3 (−19.4 to 13.2)	1397.6 (330.7 to 3987.7)	8.9 (−4.3 to 29.5)	−4.5 (−16.1 to 13.0)
Syria	858.4 (201.3 to 2341.0)	74.5 (33.0 to 133.6)	2.6 (−18.8 to 39.1)	733.2 (165.1 to 2160.7)	38.1 (4.3 to 108.0)	6.9 (−15.3 to 65.1)
Tunisia	1066.1 (246.5 to 2895.3)	146.1 (88.0 to 240.6)	−4.0 (−24.0 to 27.8)	1480.7 (346.3 to 3912.1)	189.3 (122.7 to 340.2)	−2.8 (−24.2 to 45.2)
Turkey	7586.2 (1892.8 to 21,707.6)	114.5 (79.1 to 165.9)	−3.9 (−19.6 to 16.9)	12 135.1 (3 113.1 to 32,987.7)	112.5 (72.2 to 164.5)	−3.4 (−21.5 to 20.0)
United Arab Emirates	97.7 (22.0 to 276.9)	35.1 (0.2 to 92.9)	−4.4 (−20.5 to 32.8)	40.5 (9.5 to 116.9)	4.6 (−19.7 to 48.5)	−6.5 (−23.2 to 27.4)
Yemen	773.2 (178.3 to 2221.7)	96.1 (57.5 to 146.7)	1.0 (−14.8 to 24.3)	957.4 (223.5 to 2623.4)	16.7 (−1.8 to 44.6)	1.3 (−14.8 to 24.5)
South Asia	70,103.1 (16,090.3 to 205,489.8)	136.0 (101.8 to 182.8)	12.4 (−2.0 to 35.5)	88,010.7 (21,633.3 to 238,065.3)	192.8 (135.4 to 269.7)	14.9 (−7.0 to 45.3)
Bangladesh	8204.9 (1963.9 to 23,345.3)	134.8 (92.5 to 191.2)	7.3 (−11.2 to 31.0)	7409.4 (1752.7 to 19,527.4)	137.0 (86.7 to 215.1)	6.9 (−16.2 to 44.5)
Bhutan	37.5 (8.8 to 103.8)	380.5 (277.3 to 535.1)	23.4 (−0.7 to 66.4)	43.1 (10.4 to 111.5)	192.7 (132.9 to 301.7)	28.2 (1.3 to 78.9)
India	55,824.9 (12,615.8 to 163,454.2)	191.9 (140.8 to 260.9)	18.2 (0.3 to 45.4)	73,186.4 (17,794.0 to 199,775.5)	261.7 (185.6 to 368.7)	22.8 (−2.2 to 56.5)
Nepal	965.8 (220.9 to 2837.9)	145.0 (87.7 to 226.0)	21.3 (−6.9 to 62.8)	1 482.3 (345.6 to 4119.6)	148.5 (90.2 to 236.3)	35.0 (1.9 to 88.0)
Pakistan	5070.0 (1134.4 to 14,658.6)	−29.1 (−43.4 to −10.3)	9.2 (−11.3 to 39.4)	5889.6 (1380.9 to 16,629.7)	−10.7 (−28.6 to 17.0)	13.6 (−9.3 to 49.0)
East Asia	110,468.6 (25,700.8 to 320,046.5)	194.9 (140.4 to 270.3)	5.2 (−9.3 to 25.6)	224,209.2 (53,560.4 to 585,210.4)	182.3 (127.6 to 259.5)	−1.8 (−18.6 to 22.7)
China	106,005.4 (24,780.9 to 304,484.6)	192.9 (136.8 to 269.7)	6.2 (−9.4 to 27.7)	214,709.5 (51,099.6 to 559,556.0)	180.9 (125.0 to 259.7)	−2.0 (−19.5 to 23.4)
North Korea	1093.2 (247.1 to 3139.7)	106.2 (77.4 to 140.6)	−2.5 (−15.6 to 12.6)	4114.6 (1001.2 to 11,542.2)	129.5 (92.9 to 181.7)	−3.6 (−18.6 to 18.6)
Taiwan (province of China)	3369.9 (793.9 to 9005.2)	373.0 (271.1 to 505.4)	8.5 (−11.2 to 32.7)	5385.0 (1361.0 to 13,572.1)	352.1 (268.9 to 462.2)	6.3 (−11.9 to 30.5)
Oceania	280.8 (64.8 to 769.1)	22.1 (5.8 to 42.2)	−5.5 (−16.2 to 7.8)	391.9 (91.6 to 1 051.6)	26.6 (7.9 to 51.1)	−1.9 (−16.9 to 16.0)
American Samoa	2.6 (0.6 to 7.2)	131.3 (106.8 to 158.3)	−6.9 (−17.1 to 4.0)	4.7 (1.1 to 11.9)	154.7 (113.2 to 214.7)	−4.8 (−20.2 to 15.8)
Cook Islands	1.8 (0.4 to 4.7)	156.1 (125.3 to 197.6)	−14.5 (−24.8 to −1.8)	2.7 (0.7 to 7.0)	129.1 (85.3 to 184.1)	−14.9 (−31.4 to 5.0)
Fiji	24.0 (5.4 to 65.7)	48.2 (20.3 to 84.3)	−4.7 (−18.1 to 11.2)	49.3 (11.8 to 133.6)	82.6 (44.5 to 134.7)	−9.7 (−27.8 to 14.6)
Guam	13.3 (3.2 to 35.0)	288.1 (215.5 to 384.1)	−14.0 (−27.2 to 1.0)	25.0 (6.3 to 62.4)	331.5 (254.4 to 455.8)	−19.1 (−33.2 to −1.5)
Kiribati	2.2 (0.5 to 6.2)	11.1 (−7.2 to 32.9)	8.6 (−5.2 to 30.9)	5.6 (1.3 to 15.3)	29.6 (4.1 to 63.4)	17.1 (−3.9 to 47.1)
Marshall Islands	1.4 (0.3 to 4.1)	60.2 (30.7 to 96.2)	−2.9 (−18.4 to 16.2)	1.6 (0.4 to 4.3)	0.7 (−20.3 to 24.8)	−0.7 (−18.2 to 20.7)
Federated States of Micronesia	2.6 (0.6 to 7.5)	36.6 (8.0 to 68.3)	−2.0 (−18.6 to 16.5)	5.0 (1.2 to 14.0)	57.7 (20.9 to 110.6)	3.2 (−18.9 to 37.1)
Nauru	0.1 (0.0 to 0.3)	−29.1 (−42.3 to −12.5)	−6.2 (−19.9 to 10.8)	0.1 (0.0 to 0.4)	−30.4 (−44.4 to −12.2)	−8.9 (−24.3 to 8.3)
Niue	0.2 (0.0 to 0.4)	18.5 (1.3 to 36.4)	−12.6 (−25.1 to 0.9)	0.3 (0.1 to 0.9)	−12.7 (−29.8 to 10.2)	−11.7 (−28.3 to 11.0)
Northern Mariana Islands	2.2 (0.5 to 6.1)	290.1 (245.8 to 340.3)	−4.9 (−15.0 to 6.5)	3.1 (0.8 to 8.6)	379.6 (277.0 to 513.3)	−3.0 (−22.9 to 24.0)
Palau	0.7 (0.2 to 2.0)	50.4 (21.6 to 82.3)	−7.8 (−24.8 to 9.3)	1.2 (0.3 to 3.2)	62.4 (20.3 to 110.7)	−6.5 (−30.0 to 22.1)
Papua New Guinea	179.8 (41.2 to 487.9)	12.5 (−7.0 to 36.8)	−5.2 (−19.1 to 12.6)	222.0 (50.3 to 608.5)	14.9 (−6.6 to 43.2)	4.0 (−15.4 to 29.9)
Samoa	10.5 (2.5 to 29.3)	65.6 (43.8 to 93.1)	−6.5 (−17.7 to 7.5)	17.7 (4.3 to 47.5)	30.2 (4.7 to 66.0)	−3.8 (−22.5 to 21.7)
Solomon Islands	10.6 (2.5 to 30.6)	25.4 (3.0 to 56.2)	8.1 (−5.9 to 36.9)	11.5 (2.7 to 32.2)	64.1 (27.0 to 117.1)	18.9 (−6.6 to 55.7)
Tokelau	0.1 (0.0 to 0.3)	9.6 (−5.9 to 29.3)	−10.4 (−22.6 to 4.2)	0.2 (0.0 to 0.4)	5.3 (−16.6 to 34.9)	−10.9 (−28.7 to 12.1)
Tonga	5.5 (1.3 to 15.3)	69.0 (44.0 to 97.6)	−6.3 (−18.2 to 7.5)	12.6 (3.1 to 32.2)	112.8 (74.6 to 167.0)	−1.6 (−18.7 to 22.1)
Tuvalu	0.6 (0.1 to 1.8)	73.2 (39.0 to 124.0)	−7.4 (−24.1 to 14.0)	1.0 (0.2 to 2.8)	38.1 (3.4 to 85.0)	−3.9 (−27.7 to 28.5)
Vanuatu	9.3 (2.1 to 26.4)	40.8 (16.0 to 69.4)	−0.3 (−16.2 to 18.4)	9.9 (2.3 to 29.5)	59.8 (23.2 to 109.0)	2.6 (−20.1 to 35.2)
Southeast Asia	31,415.4 (7352.9 to 86,315.6)	96.4 (74.5 to 125.1)	5.7 (−5.0 to 20.5)	61,980.9 (14,844.5 to 157,635.4)	102.5 (80.2 to 147.4)	2.2 (−8.9 to 26.0)
Cambodia	585.9 (139.5 to 1600.8)	101.6 (64.6 to 137.7)	17.6 (−3.0 to 42.1)	1208.6 (292.1 to 3263.0)	148.6 (105.5 to 218.8)	20.9 (−0.1 to 59.8)
Indonesia	8724.0 (1937.5 to 24,089.4)	73.6 (42.3 to 111.9)	18.0 (−1.0 to 43.1)	15,657.2 (3663.3 to 44,009.1)	92.6 (55.2 to 139.4)	15.5 (−6.9 to 45.5)
Laos	194.9 (47.3 to 558.3)	68.1 (35.3 to 112.3)	9.1 (−9.4 to 42.1)	334.0 (82.2 to 933.4)	64.3 (30.9 to 119.4)	14.0 (−9.8 to 56.3)
Malaysia	1639.2 (386.7 to 4309.3)	64.1 (35.2 to 99.8)	1.6 (−16.0 to 23.9)	2028.2 (477.9 to 5 620.7)	63.7 (33.2 to 97.0)	0.7 (−16.9 to 20.3)
Maldives	23.4 (5.6 to 61.0)	108.5 (71.7 to 154.4)	7.2 (−9.4 to 30.0)	24.3 (6.0 to 62.0)	347.2 (252.8 to 488.0)	2.3 (−16.9 to 28.8)
Mauritius	102.2 (24.4 to 273.0)	207.2 (161.7 to 264.4)	−0.2 (−13.1 to 16.1)	220.7 (55.3 to 560.1)	174.9 (135.5 to 227.8)	−0.7 (−13.9 to 17.8)
Myanmar	2092.8 (490.9 to 5866.7)	111.3 (79.3 to 154.4)	15.3 (−0.2 to 39.3)	4691.3 (1134.6 to 12,593.6)	110.8 (78.1 to 157.1)	6.4 (−9.4 to 31.7)
Philippines	3871.2 (888.9 to 11, 236.2)	34.3 (8.2 to 66.8)	−12.8 (−27.6 to 5.2)	6601.9 (1499.8 to 18,757.1)	54.0 (23.0 to 92.4)	−18.5 (−32.5 to −0.4)
Seychelles	5.3 (1.3 to 15.1)	7.3 (−6.0 to 23.5)	−6.0 (−17.2 to 7.1)	13.0 (3.2 to 33.6)	11.7 (−0.8 to 25.1)	0.3 (−10.9 to 12.0)
Sri Lanka	1379.4 (320.3 to 3740.9)	82.3 (43.6 to 131.7)	−3.3 (−21.8 to 20.7)	2761.5 (654.3 to 7244.7)	160.9 (105.0 to 236.6)	−3.8 (−23.5 to 24.3)
Thailand	7452.5 (1768.8 to 19,825.0)	265.5 (183.0 to 370.5)	−6.2 (−25.3 to 19.9)	14,100.2 (3615.3 to 35,484.5)	223.8 (153.7 to 317.9)	−8.0 (−27.4 to 18.9)
Timor‐Leste	43.1 (9.8 to 120.3)	144.1 (90.3 to 200.9)	14.2 (−10.3 to 41.7)	58.8 (14.4 to 163.3)	96.9 (55.2 to 153.4)	12.6 (−10.9 to 45.0)
Vietnam	5260.5 (1216.7 to 14,837.6)	88.3 (57.3 to 161.9)	15.5 (−2.4 to 60.6)	14,199.9 (3465.7 to 36,017.6)	75.1 (46.5 to 157.6)	9.2 (−8.7 to 61.0)
Central sub‐Saharan Africa	2027.3 (478.0 to 5748.1)	11.9 (−4.0 to 32.0)	9.8 (−3.6 to 26.9)	4200.4 (1045.0 to 10,907.3)	72.1 (43.7 to 111.3)	12.1 (−5.7 to 35.5)
Angola	408.7 (96.9 to 1137.6)	17.0 (−3.5 to 58.9)	19.6 (0.1 to 70.2)	734.3 (184.3 to 1954.0)	43.5 (9.2 to 108.7)	29.5 (−2.6 to 92.4)
Central African Republic	46.6 (10.7 to 135.2)	−17.2 (−30.8 to −2.7)	−7.2 (−21.3 to 10.9)	113.5 (25.2 to 325.0)	−1.3 (−23.6 to 27.6)	5.1 (−17.2 to 33.6)
Congo (Brazzaville)	120.6 (28.1 to 334.6)	61.2 (38.2 to 93.8)	10.4 (−4.2 to 29.2)	187.9 (45.3 to 503.8)	53.8 (21.6 to 104.1)	15.0 (−7.9 to 50.4)
DR Congo	1379.5 (326.7 to 3964.5)	9.6 (−8.3 to 32.3)	7.7 (−7.3 to 26.0)	3004.8 (748.8 to 7823.7)	98.2 (58.5 to 148.9)	7.9 (−11.5 to 31.5)
Equatorial Guinea	21.9 (5.0 to 60.2)	−0.8 (−22.8 to 47.2)	23.4 (−3.1 to 92.3)	43.1 (10.1 to 118.9)	37.4 (−4.9 to 113.3)	40.1 (−5.0 to 121.1)
Gabon	50.0 (11.7 to 136.7)	29.9 (11.2 to 49.1)	4.6 (−8.4 to 18.0)	116.7 (28.0 to 310.4)	7.9 (−11.5 to 36.5)	4.1 (−14.5 to 32.8)
Eastern sub‐Saharan Africa	7893.1 (1855.5 to 22,255.7)	24.4 (10.8 to 43.9)	13.9 (3.3 to 32.2)	13,536.8 (3309.1 to 36,125.5)	46.2 (28.6 to 72.0)	13.9 (0.6 to 35.2)
Burundi	191.1 (44.5 to 545.7)	−3.0 (−20.6 to 23.2)	18.0 (−0.7 to 49.7)	288.0 (68.2 to 824.3)	−5.6 (−25.5 to 23.9)	20.1 (−4.3 to 56.4)
Comoros	30.9 (7.6 to 87.6)	85.9 (53.8 to 136.5)	14.4 (−2.9 to 42.1)	54.3 (13.4 to 147.2)	122.5 (83.1 to 185.4)	17.7 (−2.3 to 52.0)
Djibouti	23.7 (5.6 to 65.2)	161.9 (114.2 to 225.0)	17.1 (−1.0 to 43.5)	34.6 (8.5 to 95.3)	110.8 (71.8 to 163.3)	14.9 (−5.6 to 43.2)
Eritrea	62.6 (13.8 to 183.8)	109.5 (66.1 to 229.1)	39.9 (10.5 to 122.0)	185.4 (41.9 to 522.8)	126.2 (53.5 to 266.7)	57.3 (7.8 to 153.8)
Ethiopia	2964.0 (694.2 to 8560.0)	111.4 (62.3 to 191.5)	18.7 (−3.7 to 64.3)	3469.0 (844.7 to 9488.3)	101.3 (52.9 to 192.8)	24.8 (−6.9 to 87.6)
Kenya	881.6 (202.4 to 2491.7)	−4.0 (−17.8 to 12.3)	10.3 (−3.2 to 25.4)	1823.7 (444.7 to 4951.5)	43.1 (22.0 to 67.6)	6.7 (−8.9 to 25.2)
Madagascar	342.7 (77.3 to 942.4)	−34.6 (−46.0 to −20.5)	1.7 (−15.6 to 20.9)	515.9 (120.4 to 1427.4)	−12.5 (−31.3 to 11.1)	7.3 (−15.0 to 35.2)
Malawi	277.6 (65.8 to 762.9)	5.2 (−8.8 to 22.2)	12.4 (−1.4 to 31.2)	741.6 (187.3 to 1994.1)	66.6 (41.3 to 100.6)	7.2 (−9.7 to 28.9)
Mozambique	448.9 (106.1 to 1313.9)	−17.3 (−29.8 to 1.2)	18.4 (0.7 to 47.4)	1080.5 (256.1 to 2929.0)	22.2 (−2.6 to 60.6)	13.0 (−9.5 to 48.6)
Rwanda	229.7 (54.2 to 660.6)	36.6 (14.8 to 75.0)	20.5 (2.6 to 59.6)	542.5 (130.7 to 1480.7)	84.3 (51.0 to 144.0)	27.5 (3.4 to 73.7)
Somalia	155.1 (35.4 to 427.7)	−28.3 (−41.6 to −10.1)	9.6 (−8.0 to 37.0)	351.1 (77.8 to 1021.0)	5.6 (−18.1 to 42.9)	15.9 (−9.8 to 58.4)
South Sudan	208.2 (47.1 to 584.5)	22.3 (−1.0 to 50.9)	4.2 (−14.7 to 27.2)	248.0 (60.5 to 662.2)	−10.0 (−27.4 to 9.7)	3.6 (−15.5 to 25.2)
Uganda	545.2 (128.8 to 1 481.6)	−10.1 (−22.5 to 6.2)	16.7 (1.5 to 40.1)	1309.2 (322.6 to 3497.3)	29.7 (8.9 to 62.5)	12.6 (−5.6 to 40.0)
Tanzania	1235.7 (291.3 to 3569.3)	28.8 (5.8 to 53.7)	1.0 (−16.4 to 19.8)	2408.8 (580.0 to 6366.5)	48.3 (29.2 to 75.4)	3.4 (−10.0 to 20.8)
Zambia	289.9 (69.2 to 818.2)	−3.9 (−18.7 to 15.1)	9.9 (−5.9 to 33.3)	473.2 (113.9 to 1309.5)	35.0 (8.3 to 70.3)	6.9 (−14.0 to 36.2)
Southern sub‐Saharan Africa	2313.5 (543.9 to 6677.3)	24.6 (15.9 to 36.2)	4.3 (−2.8 to 12.9)	5665.6 (1350.0 to 15,688.1)	42.4 (29.8 to 65.2)	3.4 (−5.6 to 19.5)
Botswana	42.3 (9.8 to 121.9)	33.6 (8.9 to 61.8)	13.4 (−6.1 to 39.4)	110.5 (26.8 to 305.4)	58.5 (16.8 to 126.8)	7.5 (−20.3 to 55.2)
eSwatini	13.3 (3.1 to 38.4)	11.3 (−3.0 to 32.6)	−1.7 (−15.4 to 15.4)	41.3 (9.2 to 119.8)	28.6 (−12.8 to 72.0)	2.8 (−30.3 to 35.8)
Lesotho	31.3 (7.4 to 90.1)	−0.6 (−14.9 to 15.8)	0.6 (−12.8 to 15.0)	97.7 (22.9 to 277.8)	−7.1 (−33.6 to 19.5)	5.4 (−23.2 to 32.2)
Namibia	67.5 (15.9 to 196.5)	51.1 (28.7 to 77.5)	13.9 (0.4 to 31.8)	160.6 (38.9 to 453.7)	85.5 (50.3 to 140.3)	14.3 (−7.1 to 48.0)
South Africa	1906.8 (451.5 to 5444.1)	23.5 (13.0 to 36.3)	4.4 (−3.5 to 14.4)	4696.3 (1111.1 to 13,008.1)	46.0 (33.0 to 70.7)	3.5 (−5.7 to 20.8)
Zimbabwe	252.3 (59.1 to 739.9)	22.9 (6.4 to 40.2)	2.2 (−10.3 to 16.7)	559.3 (135.0 to 1560.9)	21.0 (−4.4 to 50.9)	1.2 (−19.9 to 26.5)
Western sub‐Saharan Africa	10,650.8 (2584.4 to 30,235.5)	16.8 (0.5 to 38.2)	13.8 (−1.0 to 33.6)	14,867.9 (3536.1 to 39,148.4)	−1.2 (−14.0 to 18.3)	12.2 (−2.2 to 35.9)
Benin	247.8 (58.4 to 701.8)	−29.8 (−41.0 to −17.6)	5.0 (−10.1 to 22.7)	447.6 (111.1 to 1198.1)	−10.5 (−22.8 to 4.6)	3.1 (−11.5 to 21.4)
Burkina Faso	449.3 (107.4 to 1263.7)	−12.0 (−24.1 to 3.6)	−0.7 (−13.9 to 17.3)	795.4 (194.5 to 2094.2)	10.4 (−6.5 to 33.0)	−4.5 (−18.9 to 15.3)
Cape Verde	32.4 (7.8 to 89.5)	22.9 (1.7 to 44.9)	24.9 (4.2 to 46.3)	67.0 (16.4 to 176.6)	49.5 (26.1 to 91.3)	16.3 (−1.1 to 47.0)
Cameroon	569.3 (130.3 to 1636.7)	−11.1 (−26.5 to 6.6)	5.9 (−10.2 to 25.3)	935.2 (226.4 to 2606.7)	1.4 (−16.6 to 26.2)	−0.4 (−17.4 to 21.8)
Chad	330.5 (76.9 to 953.0)	−22.2 (−35.7 to −5.3)	10.3 (−8.2 to 34.4)	384.6 (92.3 to 1 043.5)	−38.2 (−47.0 to −25.7)	6.5 (−9.5 to 28.4)
Côte d'Ivoire	491.0 (117.3 to 1387.5)	50.6 (28.0 to 77.5)	5.2 (−9.2 to 22.5)	693.1 (167.5 to 908.3)	68.6 (45.5 to 97.5)	−0.9 (−14.1 to 15.2)
The Gambia	57.3 (13.9 to 157.8)	87.3 (52.3 to 131.0)	11.6 (−7.1 to 33.6)	96.9 (23.5 to 273.9)	74.7 (41.7 to 112.6)	6.6 (−13.4 to 28.8)
Ghana	737.5 (172.4 to 2040.3)	42.8 (20.2 to 73.3)	14.8 (−1.3 to 38.1)	1204.2 (293.0 to 3283.3)	54.0 (29.6 to 85.4)	0.1 (−15.4 to 19.2)
Guinea	371.9 (90.0 to 1048.8)	−7.8 (−24.1 to 11.2)	10.6 (−7.6 to 34.4)	558.7 (136.2 to 1474.4)	1.9 (−17.3 to 26.8)	5.3 (−14.3 to 32.2)
Guinea‐Bissau	27.6 (6.4 to 77.4)	−12.9 (−28.3 to 8.4)	8.1 (−8.2 to 35.0)	51.1 (12.2 to 141.1)	15.0 (−11.7 to 53.9)	6.9 (−17.5 to 45.5)
Liberia	130.4 (31.3 to 375.0)	−21.5 (−36.4 to −1.3)	3.8 (−12.5 to 28.4)	153.9 (37.3 to 409.0)	−10.6 (−25.4 to 10.2)	2.2 (−14.2 to 25.7)
Mali	562.3 (131.0 to 1607.5)	20.9 (1.1 to 45.9)	7.8 (−8.2 to 29.3)	663.9 (157.8 to 1793.0)	−5.8 (−20.2 to 14.0)	4.8 (−12.1 to 28.1)
Mauritania	162.3 (38.3 to 449.8)	65.5 (40.4 to 99.8)	4.1 (−8.8 to 23.2)	184.9 (45.0 to 493.4)	10.4 (−6.6 to 35.4)	4.7 (−11.2 to 29.6)
Niger	370.1 (86.9 to 1 028.3)	28.3 (5.4 to 68.7)	12.5 (−5.8 to 46.7)	483.4 (113.4 to 1355.0)	12.7 (−7.0 to 47.2)	12.3 (−7.3 to 48.8)
Nigeria	5260.8 (1262.9 to 14,752.3)	29.5 (−1.9 to 77.8)	20.1 (−6.6 to 61.7)	6852.6 (1552.8 to 17,791.8)	−13.8 (−32.6 to 17.5)	21.6 (−4.1 to 65.1)
São Tomé and PrÍncipe	5.5 (1.3 to 15.1)	22.9 (3.3 to 49.3)	6.3 (−7.7 to 25.3)	8.7 (2.0 to 24.1)	2.3 (−14.8 to 38.6)	9.3 (−8.9 to 48.6)
Senegal	526.7 (127.1 to 1483.8)	32.9 (13.9 to 60.8)	9.7 (−4.9 to 31.0)	719.3 (176.6 to 1 880.9)	53.3 (32.5 to 84.0)	8.5 (−6.3 to 30.1)
Sierra Leone	197.1 (47.3 to 567.3)	−23.9 (−36.9 to −8.3)	9.3 (−6.6 to 30.3)	287.9 (69.7 to 769.5)	−12.7 (−27.3 to 7.8)	7.4 (−10.1 to 32.5)
Togo	120.9 (28.2 to 348.5)	5.3 (−11.3 to 26.0)	8.1 (−7.4 to 27.5)	279.1 (68.2 to 738.9)	42.4 (22.8 to 69.1)	−2.2 (−15.1 to 16.0)

Abbreviations: SDI, Socio‐demographic Index; UI, uncertainty interval.

The total number of deaths from dementia increased with SDI quintile, from 62.1 thousand (15.1–171.3) deaths in the low SDI quintile to 563.2 thousand (148.2–1394.4) deaths in the high SDI quintile. However, the age‐standardized rates were not significantly different between SDI quintiles, indicating that differences in the numbers stem from differences in population size and population age structure. The age‐standardized rates by SDI quintile ranged from a high of 23.2 per 100,000 (5.69–60.43) in the middle SDI quintile to a low of 21.61 per 100,000 (5.22–56.36) in the low‐middle SDI quintile.

**FIGURE 4:**
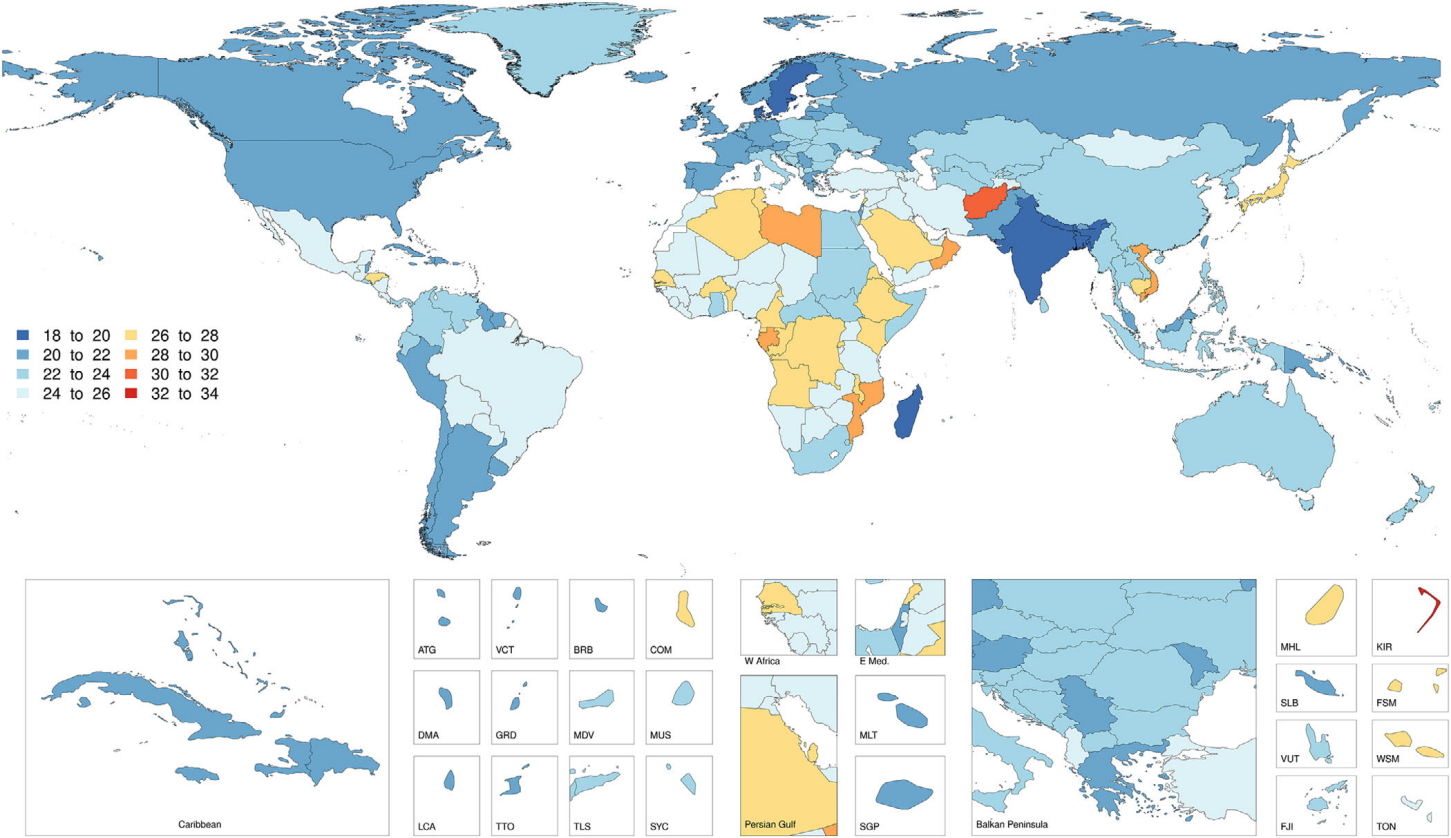
Both‐sex age‐standardized mortality rate per 100,000 due to dementia in 2019 by location. Values are expressed in rates per 100,000 population

There was a 1.7‐fold variation in age‐standardized mortality rates between countries. The countries (with population over 1 million) with the highest age‐standardized mortality rates due to dementia were Afghanistan (30.79 deaths per 100,000 [7.46‐82.34]), Mozambique (29.74 [7.29–80.08]), and Vietnam (29.21 [7.24–74.60]). The countries with the lowest age‐standardized mortality rates due to dementia were Bangladesh (18.23 deaths per 100,000 person‐years [4.50–49.29]), India (19.12 [4.57–52.01]), and Nepal (19.51 [4.53–53.31]; Figure 4).

In data from vital registration systems, huge variations in the patterns of deaths due to dementia were observed both over time and between countries. In Brazil, for example, the number of deaths due to dementia reported in vital registration data increased more than 300‐fold between 1980 and 2017. Across countries with vital registration data in 2015, the proportion of deaths attributed to dementia ranged from 0% (Qatar) to 17% (Finland). In contrast, our estimates were more stable both over time, and between countries, similar to trends observed in prevalence estimates. In the majority of locations and years, our estimated mortality due to dementia exceeded the mortality reported on death certificates in vital registration systems. Returning to the example of Brazil, our estimates were 2.12 (0.54–5.52) times the reported 21,653 deaths in vital registration data. However, in some high‐income countries in recent years our estimates were lower than those reported via vital registration systems. In the United States in 2015, our estimates were 0.54 (0.14–1.32) times the 250,863 deaths recorded in the vital registration system.

Comparing dementia to all other diseases estimated in the GBD study (at Level 3 of the GBD cause hierarchy), in 1990 dementia was the 20th leading cause of death globally comparing all‐age mortality rates and the sixth leading cause of death among individuals 70 and older. However, by 2019, deaths due to dementia were estimated to rank seventh globally across all ages and fourth among individuals 70 and older.

## 4 DISCUSSION

Globally, 1.62 million (0.41–4.21) people were estimated to have died of end‐stage dementia in 2019. Age‐standardized rates were stable over time but there were large increases in all‐age rates as a result of population aging. With this trend expected to increase in the future, an increase in dementia deaths can also be anticipated.

We found that conditions such as decubitus ulcer, bronchitis, dysphagia, hip fracture, pneumonia, and bedridden status appear in those who die with dementia substantially more than those who die without dementia. This finding is consistent with previous evidence from both autopsies and vital registration data that identifies conditions such as bronchopneumonia, aspiration pneumonia, and sepsis as common among those who die with dementia.^5^, ^61^ This convergent evidence also supports our assumption that these end‐stage conditions are indicative of individuals who died of dementia as an underlying cause of death, in line with ICD‐10 principles.^62^

Our new method of estimating dementia mortality updated the methods used in previous iterations of the GBD study. In prior iterations, we used an excess mortality regression strategy that was sensitive to the choice of which countries to include and assumed that the level of coding deaths to dementia was correct in countries that had the highest rates of coding per prevalent dementia case (i.e., there was no over‐coding).^18^, ^19^ Through the use of estimates of attributable risk from cohort studies and data on the proportion of deaths with end‐stage disease, we avoided any reliance on vital registration data and cause of death coding practices. As we have found extreme changes in mortality rates from vital registration data both over time and between countries, use of these data risks incorporating a large source of measurement error. While removing this reliance could be seen as an improvement, as we do not have a gold‐standard measure to which we can compare our estimates of dementia mortality, we do not have the ability to assess their accuracy.

As a result of this change in strategy we no longer assume that the highest rates of coding dementia as a cause of death per prevalent case is correct and we have estimated fewer deaths due to dementia. Where in previous iterations of the GBD study our estimates were always the same or higher than dementia mortality reported in vital registration data, now our estimates are in some cases lower than what is seen in vital registration data, particularly in recent years for high‐income countries, including the United States, Finland, Sweden, and the United Kingdom. This suggests that in these countries in the most recent years there may be over‐coding to dementia as an underlying cause of death. However, our estimates suggest much higher levels of dementia mortality than reported in vital registration systems in many countries, including Brazil and Germany. Improvements in the awareness of the mortality associated with dementia and clearer guidelines for death certification may help improve the quality of data from vital registration systems.

Our estimates are lower compared to prior studies that have estimated dementia‐related mortality in the United States without using vital registration data (114,838 95% uncertainty interval [UI; 30,031–281,180] deaths in adults 75 and older in 2010 vs. 503,400 deaths in James et al.;^47^ 119,916 [31,261–296,271] deaths in adults 65 and older in 2010 vs. 600,000 deaths in Weuve et al.^14^). However, both of these studies used Cox proportional hazard models to estimate the risk of mortality among individuals with dementia, and did not control for common comorbidities such as vascular diseases. While James et al. did report that adjustment for vascular comorbidities did not change results, this could be due to measurement error in the vascular risk index or the specific characteristics of the sample used.^47^ Without adjusting for common risk factor profiles, comorbid conditions, or sub‐setting to deaths with end‐stage conditions, these estimates are representative of the total number of excess deaths due to dementia and not just those attributable to dementia as an underlying cause of death.

A number of limitations still remain. First, the data on the risk of all‐cause mortality due to dementia are heterogeneous, resulting in uncertain estimates of absolute and relative mortality risk. This large source of uncertainty contributes greatly to the large uncertainty intervals for our final estimates. Second, we remain reliant on our estimates of prevalence to calculate excess deaths from data on attributable risk, and these data are also heterogeneous and sparse, particularly in low‐income settings. Third, we are assuming that the risk of dying from end‐stage dementia across locations is the same. While there are no available therapies that can slow or prevent death, differences in the composition of comorbidities and the quality of care could influence mortality.^63^, ^64^, ^65^ Fourth, we assume that administrative data can be used to accurately identify markers of end‐stage disease. Administrative data was used to classify dementia in the analysis of end‐stage disease, and prior studies have shown administrative records can have poor sensitivity, particularly when considering mild disease.^66^ However, it is more likely that individuals with severe, end‐stage dementia would be correctly classified, particularly when considering the long time span of our linked data (2003 to 2017). Fifth, due to a lack of available data sources with death record linkages, we are using data from Italy for the measurement of end‐stage conditions, and are generalizing these findings to the rest of the world. With no disease‐modifying treatments currently available, the biological drivers of disease progression are likely to be similar across locations, leading to similarities in the profiles of end‐stage conditions.^63^, ^64^ However, this assumption may be violated to the extent that health‐care practices and other cultural factors influence the proportion of individuals with dementia who have end‐stage disease. The integration of data sources from other locations should these become available would strengthen the analysis. Finally, the process of scaling all cause‐specific mortality to add up to the total estimated all‐cause mortality can be sensitive to estimates of all‐cause mortality, which are challenging at the oldest ages when dementia mortality is highest, and can affect geographic variation.^60^

In the context of population aging and growth, the importance of dementia as a public health concern will rise.^67^ Our estimates can be used by health‐ and social‐care authorities involved in end‐of‐life and palliative care to improve planning for services addressing the needs of people who die from dementia. By helping funders and policymakers better understand the global distribution of dementia burden and mortality across locations and time, and make fair comparisons between dementia and other diseases, these estimates will help guide evidence‐driven resource allocation and health system planning.

## FUNDING INFORMATION

This work was funded by the Bill and Melinda Gates Foundation, Seattle, WA, and by Gates Ventures, Seattle, WA.

## DECLARATIONS

G. J. Hankey reports personal fees from AC Immune, Lausanne, Switzerland for serving on Data Safety Monitoring Committees of ACI‐24‐701, ACI‐24‐1801, ACI‐35‐1201, and ACI‐35‐1802 trials of immune therapies (vaccines targeted to beta amyloid and tau) for Alzheimer's disease. M. Kivimäki reports grants from the UK Medical Research Council (MRC S011676), the US National Institutes on Aging (NIA R01AG056477) and NordForsk, outside the submitted work. S. Lorkowski reports personal fees from Akcea Therapeutics, Amedes, AMGEN, Berlin‐Chemie, Boehringer Ingelheim Pharma, Daiichi Sankyo, Lilly, MSD Sharp & Dohme, Novo Nordisk, Sanofi‐Aventis, Synlab, Unilever, and Upfield, and non‐financial support from Preventicus, all outside the submitted work. M. Saylan reports being an employee of Bayer AG. J. A. Singh reports personal fees from Crealta/Horizon, Medisys, Fidia, UBM LLC, Trio Health, Medscape, WebMD, Clinical Care Options, Clearview Healthcare Partners, Putnam Associates, Focus Forward, Navigant Consulting, Spherix, Practice Point Communications, the National Institutes of Health and the American College of Rheumatology, and Simply Speaking; owning stock options in Amarin, Viking, Moderna, and Vaxart pharmaceuticals; non‐financial support from FDA Arthritis Advisory Committee, Veterans Affairs Rheumatology Field Advisory Committee and from the Steering committee of OMERACT, an International organization that develops measures for clinical trials and receives arm's length funding from 12 pharmaceutical companies; and being the Editor and the Director of the UAB Cochrane Musculoskeletal Group Satellite Center on Network Meta‐analysis, all outside the submitted work. C. E. I. Szoeke reports grants from NHMRC 1062133, Alzheimer's Association, and NIA320312, during the conduct of the study. A. Wimo reports personal fees from WHO and non‐financial support from ADI, during the conduct of the study; grants from MSD and personal fees from Biogen, outside the submitted work. C. Wu reports grants from Ministry of Science and Technology in China, Suzhou Municipal Science and Technology Bureau, and the Kunshan Government, and personal fees from HealthKeepers, all outside the submitted work.

## Supporting information

Supporting information.

Supporting information.

## APPENDIX A AUTHORS AND AFFILIATIONS

Emma Nichols,^1^ Foad Abd‐Allah,^2^ Amir Abdoli,^3^ Akine Eshete Abosetugn,^4^ Woldu Aberhe Abrha,^5^ Ahmed Abualhasan,^2^ Eman Abu‐Gharbieh,^6^ Rufus Olusola Akinyemi,^7,8^ Fares Alahdab,^9^ Fahad Mashhour Alanezi,^10^ Vahid Alipour,^11 12^ Iman Ansari,^13^ Jalal Arabloo,^11^ Amir Ashraf‐Ganjouei,^14^ Abolfazl Avan,^15^ Getinet Ayano,^16^ Zaheer‐Ud‐Din Babar,^17^ Atif Amin Baig,^18^ Maciej Banach,^19 20^ Miguel A. Barboza,^21 22^ Suzanne Lyn Barker‐Collo,^23^ Bernhard T. Baune,^24 25^ Akshaya Srikanth Bhagavathula,^26 27^ Krittika Bhattacharyya,^28 29^ Ali Bijani,^30^ Antonio Biondi,^31^ Tsegaye Adane Birhan,^32^ Atanu Biswas,^33^ Srinivasa Rao Bolla,^34^ Archith Boloor,^35^ Carol Brayne,^36^ Hermann Brenner,^37^ Katrin Burkart,^1 38^ Richard A. Burns,^39^ Sharath Burugina Nagaraja,^40^ Felix Carvalho,^41^ Luis F. S. Castro‐de‐Araujo,^42^ Ferrán Catalá‐López,^43 44^ Ester Cerin,^45 46^ Achille Cernigliaro,^244^ Nicolas Cherbuin,^39^ Jee‐Young Jasmine Choi,^47^ Dinh‐Toi Chu,^48^ Baye Dagnew,^49^ Xiaochen Dai,^1^ Lalit Dandona,^50 1 51^ Rakhi Dandona,^50 1 38^ Daniel Diaz,^52 53^ Zahra Sadat Dibaji Forooshani,^54^ Abdel Douiri,^55^ Bruce B. Duncan,^56^ David Edvardsson,^57 58^ Shaimaa I. El‐Jaafary,^2^ Khalil Eskandari,^59 60^ Sharareh Eskandarieh,^61^ Valery L. Feigin,^62 1 63^ Seyed‐Mohammad Fereshtehnejad,^64 65^ Eduarda Fernandes,^66^ Pietro Ferrara,^67^ Irina Filip,^68 69^ Florian Fischer,^70^ Shilpa Gaidhane,^71^ Kidane Zereabruk Gebregzabiher,^5^ Ahmad Ghashghaee,^11 72^ Asadollah Gholamian,^73 74^ Elena V. Gnedovskaya,^75^ Mahaveer Golechha,^76^ Rajeev Gupta,^77 78^ Vladimir Hachinski,^79 80^ Samer Hamidi,^81^ Graeme J. Hankey,^82 83^ Josep Maria Haro,^84 85^ Amr Hassan,^2^ Simon I. Hay,^1 38^ Golnaz Heidari,^86^ Reza Heidari‐Soureshjani,^87^ Mowafa Househ,^88^ Rabia Hussain,^89^ Bing‐Fang Hwang,^90^ Irena M. Ilic,^91^ Milena D. Ilic,^92^ Seyed Sina Naghibi Irvani,^93^ Hiroyasu Iso,^94^ Masao Iwagami,^95 96^ Ravi Prakash Jha,^97 98^ Rizwan Kalani,^99^ Himal Kandel,^100 101^ André Karch,^102^ Ayele Semachew Kasa,^103^ Andre Pascal Kengne,^104 105^ Young‐Eun Kim,^106^ Yun Jin Kim,^107^ Sezer Kisa,^108^ Adnan Kisa,^109 110^ Mika Kivimäki,^111 112^ Hamidreza Komaki,^113 114^ Ai Koyanagi,^115 116^ Walter A Kukull,^117^ G. Anil Kumar,^50^ Manasi Kumar,^118 119^ Iván Landires,^120 121^ Matilde Leonardi,^122^ Stephen S. Lim,^1 38^ Xuefeng Liu,^123^ Giancarlo Logroscino,^124 125^ Alan D. Lopez,^126 1 38^ Stefan Lorkowski,^127 128^ Clement T. Loy^129 130^, Hawraz Ibrahim M. Amin,^131 132^ Navid Manafi,^133 134^ Narayana Manjunatha,^135^ Man Mohan Mehndiratta,^136 137^ Ritesh G Menezes,^138^ Atte Meretoja,^139 140^ Alexander Merkin,^62^ Workua Mekonnen Metekiya,^141^ Awoke Temesgen Misganaw,^38 142^ Bahram Mohajer,^143^ Norlinah Mohamed Ibrahim,^144^ Yousef Mohammad,^145^ Archisman Mohapatra,^146^ Farnam Mohebi,^143 147^ Ali H. Mokdad,^1 38^ Stefania Mondello,^148^ Tilahun Belete Mossie,^149^ Anwar Mulugeta,^150 151^ Gabriele Nagel,^152^ Muhammad Naveed,^153^ Vinod C. Nayak,^154^ Sandhya Neupane Kandel,^155^ Son Hoang Nguyen,^156^ Huong Lan Thi Nguyen,^157^ Virginia Nuñez‐Samudio,^158 121^ Felix Akpojene Ogbo,^159^ Andrew T. Olagunju,^160 161^ Hans Orru,^162 163^ Sergej M. Ostojic,^164^ Samuel M. Ostroff,^1 165^ Nikita Otstavnov,^166^ Stanislav S Otstavnov,^166 167^ Mayowa O. Owolabi,^168 169^ Mona Pathak,^170^ Hamidreza Pazoki Toroudi,^171 172^ Carrie B. Peterson,^173^ Hai Quang Pham,^157^ Michael R. Phillips,^174 175^ Michael A. Piradov,^63^ Faheem Hyder Pottoo,^176^ Sergio I. Prada,^177 178^ Dimas Ria Angga Pribadi,^179^ Amir Radfar,^180^ Alberto Raggi,^122^ Fakher Rahim,^181 182^ Pradhum Ram,^183^ Juwel Rana,^184 185^ Vahid Rashedi,^186^ Salman Rawaf,^187 188^ David Laith Rawaf,^189 190^ Nickolas Reinig,^1^ Nima Rezaei,^191 192^ Stephen R Robinson,^193^ Michele Romoli,^194 195^ Perminder S. Sachdev,^196 197^ Ramesh Sahathevan,^198 140^ Amirhossein Sahebkar,^199 200^ Mohammad Ali Sahraian,^61^ Davide Sattin,^122^ Mete Saylan,^201^ Mehdi Sayyah,^202^ Silvia Schiavolin,^122^ Maria Inês Schmidt,^56^ Izza Shahid,^203^ Masood Ali Shaikh,^204^ Mika Shigematsu,^205^ Jae Il Shin,^206^ Rahman Shiri,^207^ Tariq Jamal Siddiqi,^208^ João Pedro Silva,^41^ Jasvinder A. Singh,^209 210^, Amin Soheili^211^, Emma Elizabeth Spurlock^1^, Cassandra E I Szoeke^212 213^, Rafael Tabarés‐Seisdedos^214 215^, Biruk Wogayehu Taddele,^216^ Bhaskar Thakur,^217 218^ Akhil Soman ThekkePurakkal,^219^ Marcos Roberto Tovani‐Palone,^220 221^ Bach Xuan Tran,^222^ Ravensara S. Travillian,^1^ Manjari Tripathi,^223^ Gebiyaw Wudie Tsegaye,^224^ Muhammad Shariq Usman,^225^ Marco Vacante,^31^ Diana Zuleika Velazquez,^53^ Narayanaswamy Venketasubramanian,^226 227^ Simone Vidale,^228 229^ Vasily Vlassov,^230^ Yuan‐Pang Wang,^231^ Jingkai Wei,^232^ Jordan Weiss,^233^ Abrha Hailay Weldemariam,^5^ Anders Wimo,^234^ Chenkai Wu,^235 236^ Ali Yadollahpour,^237^ Kazumasa Yamagishi,^238 239^ Yordanos Gizachew Yeshitila,^240^ Naohiro Yonemoto,^241 242^ Siddhesh Zadey,^236^ Zhi‐Jiang Zhang,^243^ Christopher J. L. Murray,^1 38^ and Theo Vos.^1 38^

**Affiliations**

1. Institute for Health Metrics and Evaluation, University of Washington, Seattle, WA, USA.
2. Department of Neurology, Cairo University, Cairo, Egypt.
3. Department of Parasitology and Mycology, Jahrom University of Medical Sciences, Jahrom, Iran.
4. Department of Public Health, Debre Berhan University, Debre Berhan, Ethiopia.
5. Department of Adult Health Nursing, Aksum University, Aksum, Ethiopia.
6. Department of Clinical Sciences, University of Sharjah, Sharjah, United Arab Emirates.
7. Institute for Advanced Medical Research and Training, University of Ibadan, Ibadan, Nigeria.
8. Institute of Neuroscience, Newcastle University, Newcastle upon Tyne, UK.
9. Mayo Evidence‐based Practice Center, Mayo Clinic Foundation for Medical Education and Research, Rochester, MN, USA.
10. Imam Abdulrahman Bin Faisal University, Dammam, Saudi Arabia.
11. Health Management and Economics Research Center, Iran University of Medical Sciences, Tehran, Iran.
12. Health Economics Department, Iran University of Medical Sciences, Tehran, Iran.
13. Medical Students Research Committee, Shahed University, Tehran, Iran.
14. Students’ Scientific Research Center, Tehran University of Medical Sciences, Tehran, Iran.
15. Department of Public Health, Mashhad University of Medical Sciences, Mashhad, Iran.
16. School of Public Health, Curtin University, Perth, WA, Australia.
17. Department of Pharmacy, University of Huddersfield, Huddersfield, UK.
18. Unit of Biochemistry, Sultan Zainal Abidin University (Universiti Sultan Zainal Abidin), Kuala Terengganu, Malaysia.
19. Department of Hypertension, Medical University of Lodz, Lodz, Poland.
20. Polish Mothers’ Memorial Hospital Research Institute, Lodz, Poland.
21. Department of Neurosciences, Costa Rican Department of Social Security, San Jose, Costa Rica.
22. School of Medicine, University of Costa Rica, San Pedro, Costa Rica.
23. School of Psychology, University of Auckland, Auckland, New Zealand.
24. Department of Psychiatry, University of Münster, Münster, Germany.
25. Department of Psychiatry, Melbourne Medical School, Melbourne, Australia.
26. Department of Social and Clinical Pharmacy, Charles University, Hradec Kralova, Czech Republic.
27. Institute of Public Health, United Arab Emirates University, Al Ain, United Arab Emirates.
28. Department of Statistical and Computational Genomics, National Institute of Biomedical Genomics, Kalyani, India.
29. Department of Statistics, University of Calcutta, Kolkata, India.
30. Social Determinants of Health Research Center, Babol University of Medical Sciences, Babol, Iran.
31. Department of General Surgery and Medical‐Surgical Specialties, University of Catania, Catania, Italy.
32. Department of Environmental and Occupational Health and Safety, University of Gondar, Gondar, Ethiopia.
33. Department of Neurology, Institute of Post‐Graduate Medical Education and Research and Seth Sukhlal Karnani Memorial Hospital, Kolkata, India.
34. Department of Biomedical Sciences, Nazarbayev University, Nur‐Sultan City, Kazakhstan.
35. Department of Internal Medicine, Manipal Academy of Higher Education, Mangalore, India.
36. Department of Public Health and Primary Care, University of Cambridge, Cambridge, UK.
37. Division of Clinical Epidemiology and Aging Research, German Cancer Research Center, Heidelberg, Germany.
38. Department of Health Metrics Sciences, School of Medicine, University of Washington, Seattle, WA, USA.
39. Research School of Population Health, Australian National University, Canberra, ACT, Australia.
40. Department of Community Medicine, Employee State Insurance Post Graduate Institute of Medical Sciences and Research, Bangalore, India.
41. Research Unit on Applied Molecular Biosciences (UCIBIO), University of Porto, Porto, Portugal.
42. Department of Psychiatry, University of Melbourne, Melbourne, VIC, Australia.
43. National School of Public Health, Institute of Health Carlos III, Madrid, Spain.
44. Clinical Epidemiology Program, Ottawa Hospital Research Institute, Ottawa, ON, Canada.
45. Mary MacKillop Institute for Health Research, Australian Catholic University, Melbourne, VIC, Australia.
46. School of Public Health, University of Hong Kong, Hong Kong, China.
47. Biomedical Informatics, Seoul National University Hospital, Seoul, South Korea.
48. Faculty of Biology, Hanoi National University of Education, Hanoi, Vietnam.
49. Department of Human Physiology, University of Gondar, Gondar, Ethiopia.
50. Public Health Foundation of India, Gurugram, India.
51. Indian Council of Medical Research, New Delhi, India.
52. Center of Complexity Sciences, National Autonomous University of Mexico, Mexico City, Mexico.
53. Faculty of Veterinary Medicine and Zootechnics, Autonomous University of Sinaloa, Culiacán Rosales, Mexico.
54. Tehran University of Medical Sciences, Tehran, Iran.
55. School of Population Health and Environmental Sciences, King's College London, London, UK.
56. Postgraduate Program in Epidemiology, Federal University of Rio Grande do Sul, Porto Alegre, Brazil.
57. School of Nursing and Midwifery, La Trobe University, Melbourne, VIC, Australia.
58. Department of Nursing, Umeå University, Umea, Sweden.
59. Department of Medicinal Chemistry, Kerman University of Medical Sciences, Kerman, Iran.
60. Pharmaceutics Research Center, Kerman University of Medical Sciences, Kerman, Iran.
61. Multiple Sclerosis Research Center, Tehran University of Medical Sciences, Tehran, Iran.
62. National Institute for Stroke and Applied Neurosciences, Auckland University of Technology, Auckland, New Zealand.
63. Research Center of Neurology, Moscow, Russia.
64. Department of Neurobiology, Karolinska Institute, Stockholm, Sweden.
65. Division of Neurology, University of Ottawa, Ottawa, ON, Canada.
66. Associated Laboratory for Green Chemistry (LAQV), University of Porto, Porto, Portugal.
67. Research Center on Public Health, University of Milan Bicocca, Monza, Italy.
68. Psychiatry Department, Kaiser Permanente, Fontana, CA, USA.
69. School of Health Sciences, A.T. Still University, Mesa, AZ, USA.
70. Institute of Gerontological Health Services and Nursing Research, Ravensburg‐Weingarten University of Applied Sciences, Weingarten, Germany.
71. Department of Medicine, Datta Meghe Institute of Medical Science, Wardha, India.
72. Student Research Committee, Iran University of Medical Sciences, Tehran, Iran.
73. Young Researchers and Elite Club, Islamic Azad University, Rasht, Iran.
74. Department of Biology, Islamic Azad University, Tehran, Iran.
75. Third Department of Neurology, Research Center of Neurology, Moscow, Russia.
76. Health Systems and Policy Research, Indian Institute of Public Health Gandhinagar, Gandhinagar, India.
77. Department of Preventive Cardiology, Eternal Heart Care Centre & Research Institute, Jaipur, India.
78. Department of Medicine, Mahatma Gandhi University Medical Sciences, Jaipur, India.
79. Clinical Neurological Sciences, The University of Western Ontario, London, ON, Canada.
80. Lawson Health Research Institute, London, ON, Canada.
81. School of Health and Environmental Studies, Hamdan Bin Mohammed Smart University, Dubai, United Arab Emirates.
82. Medical School, University of Western Australia, Perth, WA, Australia.
83. Department of Neurology, Sir Charles Gairdner Hospital, Perth, WA, Australia.
84. Research Unit, University of Barcelona, Sant Boi de Llobregat, Barcelona, Spain.
85. Biomedical Research Networking Center for Mental Health Network (CiberSAM), Barcelona, Spain.
86. Independent Consultant, Santa Clara, CA, USA.
87. School of Nursing and Midwifery, Tehran University of Medical Sciences, Tehran, Iran.
88. College of Science and Engineering, Hamad Bin Khalifa University, Doha, Qatar.
89. School of Pharmaceutical Sciences, University of Science Malaysia, Penang, Malaysia.
90. Department of Occupational Safety and Health, China Medical University, Taichung, Taiwan.
91. Faculty of Medicine, University of Belgrade, Belgrade, Serbia.
92. Department of Epidemiology, University of Kragujevac, Kragujevac, Serbia.
93. Research Institute for Endocrine Sciences, Shahid Beheshti University of Medical Sciences, Tehran, Iran.
94. Public Health Department of Social Medicine, Osaka University, Suita, Japan.
95. Department of Health Services Research, University of Tsukuba, Tsukuba, Japan.
96. Department of Non‐Communicable Disease Epidemiology, London School of Hygiene & Tropical Medicine, London, UK.
97. Department of Community Medicine, Baba Saheb Ambedkar Medical College & Hospital, Delhi, India.
98. Department of Community Medicine, Banaras Hindu University, Varanasi, India.
99. Department of Neurology, University of Washington, Seattle, WA, USA.
100. Ophthalmology Department, University of Sydney, Sydney, NSW, Australia.
101. Ophthalmology Department, Sydney Local Health District, Sydney, NSW, Australia.
102. Institute for Epidemiology and Social Medicine, University of Münster, Münster, Germany.
103. Department of Adult Health Nursing, Bahir Dar University, Bahir Dar, Ethiopia.
104. Non‐Communicable Diseases Research Unit, Medical Research Council South Africa, Cape Town, South Africa.
105. Department of Medicine, University of Cape Town, Cape Town, South Africa.
106. Big Data Department, National Health Insurance Service, Wonju, South Korea.
107. School of Traditional Chinese Medicine, Xiamen University Malaysia, Sepang, Malaysia.
108. Department of Nursing and Health Promotion, Oslo Metropolitan University, Oslo, Norway.
109. School of Health Sciences, Kristiania University College, Oslo, Norway.
110. Global Community Health and Behavioral Sciences, Tulane University, New Orleans, LA, USA.
111. Department of Epidemiology and Public Health, University College London, London, UK.
112. Department of Public Health, University of Helsinki, Helsinki, Finland.
113. Neurophysiology Research Center, Hamadan University of Medical Sciences, Hamadan, Iran.
114. Brain Engineering Research Center, Institute for Research in Fundamental Sciences, Tehran, Iran.
115. CIBERSAM, San Juan de Dios Sanitary Park, Sant Boi de Llobregat, Spain.
116. Catalan Institution for Research and Advanced Studies (ICREA), Barcelona, Spain.
117. Department of Epidemiology, University of Washington, Seattle, WA, USA.
118. Department of Psychiatry, University of Nairobi, Nairobi, Kenya.
119. Division of Psychology and Language Sciences, University College London, London, UK.
120. Unit of Genetics and Public Health, Institute of Medical Sciences, Las Tablas, Panama.
121. Ministry of Health, Herrera, Panama.
122. Neurology, Public Health and Disability Unit, Carlo Besta Neurological Institute IRCCS, Milan, Italy.
123. Department of Systems, Populations, and Leadership, University of Michigan, Ann Arbor, MI, USA.
124. Department of Basic Medical Sciences, Neuroscience and Sense Organs, University of Bari Aldo Moro, Bari, Italy.
125. Department of Clinical Research in Neurology, Fondazione Cardinale Giovanni Panico Hospital, Tricase, Italy.
126. Melbourne School of Population and Global Health, University of Melbourne, Melbourne, VIC, Australia.
127. Institute of Nutritional Sciences, Friedrich Schiller University Jena, Jena, Germany.
128. Competence Cluster for Nutrition and Cardiovascular Health (nutriCARD), Jena, Germany.
129. Brain and Mind Centre, University of Sydney, Sydney, NSW, Australia.
130. The Garvan Institute of Medical Research, Sydney, NSW, Australia.
131. Department of Pharmaceutical Science, University of Eastern Piedmont, Novara, Italy.
132. Chemistry Department, Salahaddin University‐Erbil, Erbil, Iraq.
133. School of Medicine, Iran University of Medical Sciences, Tehran, Iran.
134. School of Medicine, University of Manitoba, Winnipeg, MB, Canada.
135. Department of Psychiatry, National Institute of Mental Health and Neurosciences, Bengalore, India.
136. Neurology Department, Janakpuri Super Specialty Hospital Society, New Delhi, India.
137. Department of Neurology, Govind Ballabh Institute of Medical Education and Research, New Delhi, India.
138. Forensic Medicine Division, Imam Abdulrahman Bin Faisal University, Dammam, Saudi Arabia.
139. Neurology Unit, Helsinki University Hospital, Helsinki, Finland.
140. School of Health Sciences, University of Melbourne, Melbourne, VIC, Australia.
141. Department of Psychiatry, Mekelle University, Mekelle, Ethiopia.
142. National Data Management Center, Ethiopian Public Health Institute, Addis Ababa, Ethiopia.
143. Non‐communicable Diseases Research Center, Tehran University of Medical Sciences, Tehran, Iran.
144. Department of Medicine, National University of Malaysia Medical Center, Bandar Tun Razak, Malaysia.
145. Internal Medicine Department, King Saud University, Riyadh, Saudi Arabia.
146. Epidemiology Department, GRID Council, Bhubaneswar, India.
147. National Institute of Health Research (NIHR), Tehran University of Medical Sciences, Tehran, Iran.
148. Department of Biomedical and Dental Sciences and Morphofunctional Imaging, Messina University, Messina, Italy.
149. Department of Psychiatry, Bahir Dar University, Bahir Dar, Ethiopia.
150. Australian Centre for Precision Health, University of South Australia, Adelaide, SA, Australia.
151. Department of Pharmacology and Clinical Pharmacy, Addis Ababa University, Addis Ababa, Ethiopia.
152. Institute of Epidemiology and Medical Biometry, Ulm University, Ulm, Germany.
153. Department of Biotechnology, University of Central Punjab, Lahore, Pakistan.
154. Department of Forensic Medicine and Toxicology, Manipal Academy of Higher Education, Manipal, India.
155. Bupa Clemton Park, Bupa, Sydney, NSW, Australia.
156. Center of Excellence in Behavioral Medicine, Nguyen Tat Thanh University, Ho Chi Minh City, Vietnam.
157. Institute for Global Health Innovations, Duy Tan University, Hanoi, Vietnam.
158. Unit of Microbiology and Public Health, Institute of Medical Sciences, Las Tablas, Panama.
159. Translational Health Research Institute, Western Sydney University, Sydney, NSW, Australia.
160. Department of Psychiatry and Behavioural Neurosciences, McMaster University, Hamilton, ON, Canada.
161. Department of Psychiatry, University of Lagos, Lagos, Nigeria.
162. Institute of Family Medicine and Public Health, University of Tartu, Tartu, Estonia.
163. Section of Sustainable Health, Umeå University, Umea, Sweden.
164. Department of Biomedical Sciences, University of Novi Sad, Novi Sad, Serbia.
165. Henry M Jackson School of International Studies, University of Washington, Seattle, WA, USA.
166. Laboratory of Public Health Indicators Analysis and Health Digitalization, Moscow Institute of Physics and Technology, Dolgoprudny, Russia.
167. Department of Project Management, National Research University Higher School of Economics, Moscow, Russia.
168. Department of Medicine, University of Ibadan, Ibadan, Nigeria.
169. Department of Medicine, University College Hospital Ibadan, Ibadan, Nigeria.
170. Research & Development Department, Kalinga Institute of Medical Sciences, Bhubaneswar, India.
171. Department of Physiology, Iran University of Medical Sciences, Tehran, Iran.
172. Physiology Research Center, Iran University of Medical Sciences, Tehran, Iran.
173. Gerontology, Dementia and Digital Health, Independent Consultant, Copenhagen, Denmark.
174. Shanghai Mental Health Center, Shanghai Jiao Tong University, Shanghai, China.
175. Department of Psychiatry, Columbia University, New York City, NY, USA.
176. Department of Pharmacology, Imam Abdulrahman Bin Faisal University, Dammam, Saudi Arabia.
177. Clinical Research Center, Valle del Lili Foundation (Centro de Investigaciones Clinicas, Fundación Valle del Lili), Cali, Colombia.
178. PROESA, ICESI University, (Centro PROESA, Universidad ICESI), Cali, Colombia.
179. Health Sciences Department, Muhammadiyah University of Surakarta, Sukoharjo, Indonesia.
180. College of Medicine, University of Central Florida, Orlando, FL, USA.
181. Thalassemia and Hemoglobinopathy Research Center, Ahvaz Jundishapur University of Medical Sciences, Ahvaz, Iran.
182. Metabolomics and Genomics Research Center, Tehran University of Medical Sciences, Tehran, Iran.
183. Department of Cardiology, Emory University, Atlanta, GA, USA.
184. Department of Public Health, North South University, Dhaka, Bangladesh.
185. Department of Biostatistics and Epidemiology, University of Massachusetts Amherst, Amherst, MA, USA.
186. Tehran Institute of Psychiatry, Iran University of Medical Sciences, Tehran, Iran.
187. Department of Primary Care and Public Health, Imperial College London, London, UK.
188. Academic Public Health England, Public Health England, London, UK.
189. WHO Collaborating Centre for Public Health Education and Training, Imperial College London, London, UK.
190. University College London Hospitals, London, UK.
191. Research Center for Immunodeficiencies, Tehran University of Medical Sciences, Tehran, Iran.
192. Network of Immunity in Infection, Malignancy and Autoimmunity (NIIMA), Universal Scientific Education and Research Network (USERN), Tehran, Iran.
193. Department of Psychology, Royal Melbourne Institute of Technology University, Bundoora, VIC, Australia.
194. Department of Neuroscience, University of Perugia, Perugia, Italy.
195. Department of Neurology, Rimini ‘Infermi’ Hospital AUSL Romagna, Rimini, Italy.
196. School of Psychiatry, University of New South Wales, Kensington, NSW, Australia.
197. Neuropsychiatric Institute, Prince of Wales Hospital, Randwick, NSW, Australia.
198. Internal Medicine Servcies, Ballarat Health Service, Ballarat, VIC, Australia.
199. Halal Research Center of IRI, Food and Drug Administration of the Islamic Republic of Iran, Tehran, Iran.
200. Neurogenic Inflammation Research Center, Mashhad University of Medical Sciences, Mashhad, Iran.
201. Market Access, Bayer, Istanbul, Turkey.
202. Education Development Center, Ahvaz Jundishapur University of Medical Sciences, Ahvaz, Iran.
203. Department of Internal Medicine, Ziauddin University, Karachi, Pakistan.
204. Independent Consultant, Karachi, Pakistan.
205. National Institute of Infectious Diseases, Tokyo, Japan.
206. College of Medicine, Yonsei University, Seoul, South Korea.
207. Finnish Institute of Occupational Health, Helsinki, Finland.
208. Department of Medicine, Dow University of Health Sciences, Karachi, Pakistan.
209. School of Medicine, University of Alabama at Birmingham, Birmingham, AL, USA.
210. Medicine Service, US Department of Veterans Affairs (VA), Birmingham, AL, USA.
211. Nursing Care Research Center, Semnan University of Medical Sciences, Semnan, Iran.
212. Faculty of Medicine, Dentistry and Health Sciences, University of Melbourne, Melbourne, VIC, Australia.
213. The Brain Institute, Australian Healthy Ageing Organisation, Melbourne, VIC, Australia.
214. Department of Medicine, University of Valencia, Valencia, Spain.
215. Carlos III Health Institute, Biomedical Research Networking Center for Mental Health Network (CiberSAM), Madrid, Spain.
216. Department of Pharmacy, Arbaminch College of Health Sciences, Arba Minch, Ethiopia.
217. Division of Biostatistics and Epidemiology, Texas Tech University, El Paso, TX, USA.
218. Massachusetts Veterans Epidemiology Research and Information Center (MAVERIC) & CSP Coordinating Center, Harvard University, Boston, MA, USA.
219. Disease Burden Department, Public Health Foundation of India, Gurugram, India.
220. Department of Pathology and Legal Medicine, University of São Paulo, Ribeirão Preto, Brazil.
221. Modestum LTD, London, UK.
222. Department of Health Economics, Hanoi Medical University, Hanoi, Vietnam.
223. Department of Neurology, All India Institute of Medical Sciences, Delhi, India.
224. College of Medicine and Health Sciences, Bahir Dar University, Bahir Dar, Ethiopia.
225. Department of Internal Medicine, Dow University of Health Sciences, Karachi, Pakistan.
226. Raffles Neuroscience Centre, Raffles Hospital, Singapore, Singapore.
227. Yong Loo Lin School of Medicine, National University of Singapore, Singapore, Singapore.
228. Department of Neurology, Infermi Hospital, Rimini, Italy.
229. Department of Neurology & Stroke Unit, Sant'Anna Hospital, Como, Italy.
230. Department of Health Care Administration and Economics, National Research University Higher School of Economics, Moscow, Russia.
231. Department of Psychiatry, University of São Paulo, São Paulo, Brazil.
232. Department of Epidemiology and Biostatistics, George Washington University, Washington, DC, USA.
233. Department of Demography, University of California Berkeley, Berkley, CA, USA.
234. Department of Neurobiology, Care Sciences and Society, Karolinska Institute, Solna, Sweden.
235. Global Health Research Center, Duke Kunshan University, Kunshan, China.
236. Duke Global Health Institute, Duke University, Durham, NC, USA.
237. Psychology Department, University of Sheffield, Sheffield, UK.
238. Research and Development Center for Health Services, University of Tsukuba, Tsukuba, Japan.
239. Graduate School of Medicine, Osaka University, Suita, Japan.
240. Department of Nursing, Arba Minch University, Arba Minch, Ethiopia.
241. Department of Neuropsychopharmacology, National Center of Neurology and Psychiatry, Kodaira, Japan.
242. Department of Public Health, Juntendo University, Tokyo, Japan.
243. School of Medicine, Wuhan University, Wuhan, China.
244. Regional Epidemiological Observatory Department, Sicilian Regional Health Authority, Palermo, Italy.

### AUTHOR CONTRIBUTIONS

Writing the first draft of the manuscript

Emma Nichols

Extracting, cleaning, or cataloging data; designing or coding figures and tables

Emma Nichols and Nickolas Reinig

Managing the estimation and publication process

Theo Vos

Providing data or critical feedback on data sources

Amir Abdoli, Akine Eshete Abosetugn, Woldu Aberhe Abrha, Ahmed Abualhasan, Fares Alahdab, Fahad Mashhour Alanezi, Jalal Arabloo, Getinet Ayano, Maciej Banach, Miguel A Barboza, Akshaya Srikanth Bhagavathula, Tsegaye Adane Birhan, Archith Boloor, Sharath Burugina Nagaraja, Ferrán Catalá‐López, Dinh‐Toi Chu, Xiaochen Dai, Rakhi Dandona, Lalit Dandona, Zahra Sadat Dibaji Forooshani, David Edvardsson, Khalil Eskandari, Seyed‐Mohammad Fereshtehnejad, Irina Filip, Shilpa Gaidhane, Ahmad Ghashghaee, Asadollah Gholamian, Yordanos Gizachew Yeshitila, Elena V Gnedovskaya, Mahaveer Golechha, Josep Maria Haro, Reza Heidari‐Soureshjani, Mowafa Househ, Seyed Sina Naghibi Irvani, Rizwan Kalani, André Karch, Ayele Semachew Kasa, Young‐Eun Kim, Yun Jin Kim, Sezer Kisa, Adnan Kisa, Walter A Kukull, G Anil Kumar, Stephen S Lim, Xuefeng Liu, Alan D Lopez, Stefan Lorkowski, Navid Manafi, Narayana Manjunatha, Man Mohan Mehndiratta, Ritesh G Menezes, Atte Meretoja, Workua Mekonnen Metekiya, Awoke Temesgen Misganaw, Norlinah Mohamed Ibrahim, Ali H Mokdad, Tilahun Belete Mossie, Gabriele Nagel, Son Hoang Nguyen, Huong Lan Thi Nguyen, Andrew T Olagunju, Hans Orru, Sergej M Ostojic, Carrie B Peterson, Hai Quang Pham, Michael A Piradov, Sergio I Prada, Amir Radfar, Fakher Rahim, Salman Rawaf, David Laith Rawaf, Nima Rezaei, Mete Saylan, Masood Ali Shaikh, Jae Il Shin, Jasvinder A Singh, Amin Soheili, Cassandra E I Szoeke, Rafael Tabarés‐Seisdedos, Akhil Soman ThekkePurakkal, Marcos Roberto Tovani‐Palone, Bach Xuan Tran, Ravensara S Travillian, Gebiyaw Wudie Tsegaye, Narayanaswamy Venketasubramanian, Vasily Vlassov, Jordan Weiss, Ali Yadollahpour, Naohiro Yonemoto, Zhi‐Jiang Zhang, Christopher J L Murray and Theo Vos.

Providing critical feedback on methods or results

Emma Nichols, Foad Abd‐Allah, Akine Eshete Abosetugn, Woldu Aberhe Abrha, Ahmed Abualhasan, Eman Abu‐Gharbieh, Rufus Olusola Akinyemi, Fares Alahdab, Fahad Mashhour Alanezi, Vahid Alipour, Iman Ansari, Jalal Arabloo, Amir Ashraf‐Ganjouei, Abolfazl Avan, Getinet Ayano, Zaheer‐Ud‐Din Babar, Atif Amin Baig, Maciej Banach, Miguel A Barboza, Suzanne Lyn Barker‐Collo, Bernhard T Baune, Akshaya Srikanth Bhagavathula, Krittika Bhattacharyya, Ali Bijani, Antonio Biondi, Tsegaye Adane Birhan, Atanu Biswas, Srinivasa Rao Bolla, Archith Boloor, Carol Brayne, Hermann Brenner, Katrin Burkart, Richard A Burns, Sharath Burugina Nagaraja, Luis F S Castro‐de‐Araujo, Ferrán Catalá‐López, Ester Cerin, Nicolas Cherbuin, Dinh‐Toi Chu, Baye Dagnew, Xiaochen Dai, Rakhi Dandona, Lalit Dandona, Daniel Diaz, Zahra Sadat Dibaji Forooshani, Abdel Douiri, David Edvardsson, Khalil Eskandari, Sharareh Eskandarieh, Valery L Feigin, Seyed‐Mohammad Fereshtehnejad, Irina Filip, Florian Fischer, Shilpa Gaidhane, Ahmad Ghashghaee, Yordanos Gizachew Yeshitila, Mahaveer Golechha, Rajeev Gupta, Vladimir Hachinski, Abrha Hailay Weldemariam, Simon I Hay, Golnaz Heidari, Reza Heidari‐Soureshjani, Mowafa Househ, Rabia Hussain, Bing‐Fang Hwang, Milena D Ilic, Irena M Ilic, Seyed Sina Naghibi Irvani, Masao Iwagami, Ravi Prakash Jha, Rizwan Kalani, Himal Kandel, André Karch, Ayele Semachew Kasa, Young‐Eun Kim, Yun Jin Kim, Sezer Kisa, Adnan Kisa, Mika Kivimäki, Hamidreza Komaki, Ai Koyanagi, Walter A Kukull, G Anil Kumar, Manasi Kumar, Stephen S Lim, Xuefeng Liu, Giancarlo Logroscino, Alan D Lopez, Stefan Lorkowski, Clement T Loy, Hawraz Ibrahim M. Amin, Navid Manafi, Narayana Manjunatha, Man Mohan Mehndiratta, Ritesh G Menezes, Atte Meretoja, Workua Mekonnen Metekiya, Bahram Mohajer, Yousef Mohammad, Archisman Mohapatra, Farnam Mohebi, Ali H Mokdad, Stefania Mondello, Tilahun Belete Mossie, Anwar Mulugeta, Gabriele Nagel, Son Hoang Nguyen, Huong Lan Thi Nguyen, Dr Muhammad Naveed, Sandhya Neupane Kandel, Felix Akpojene Ogbo, Andrew T Olagunju, Sergej M Ostojic, Samuel M Ostroff, Nikita Otstavnov, Stanislav S Otstavnov, Mayowa O Owolabi, Mona Pathak, Carrie B Peterson, Hai Quang Pham, Michael R Phillips, Faheem Hyder Pottoo, Sergio I Prada, Dimas Ria Angga Pribadi, Amir Radfar, Fakher Rahim, Pradhum Ram, Juwel Rana, Vahid Rashedi, Salman Rawaf, David Laith Rawaf, Nima Rezaei, Stephen R Robinson, Michele Romoli, Perminder S Sachdev, Ramesh Sahathevan, Mohammad Ali Sahraian, Mete Saylan, Izza Shahid, Masood Ali Shaikh, Mika Shigematsu, Jae Il Shin, Rahman Shiri, Tariq Jamal Siddiqi, Jasvinder A Singh, Amin Soheili, Emma Elizabeth Spurlock, Cassandra E I Szoeke, Rafael Tabarés‐Seisdedos, Bhaskar Thakur, Akhil Soman ThekkePurakkal, Marcos Roberto Tovani‐Palone, Bach Xuan Tran, Ravensara S Travillian, Manjari Tripathi, Gebiyaw Wudie Tsegaye, Muhammad Shariq Usman, Marco Vacante, Diana Zuleika Velazquez, Narayanaswamy Venketasubramanian, Yuan‐Pang Wang, Jingkai Wei, Jordan Weiss, Anders Wimo, Biruk Wogayehu Taddele, Chenkai Wu, Ali Yadollahpour, Kazumasa Yamagishi, Naohiro Yonemoto, Siddhesh Zadey, Kidane Zereabruk Gebregzabiher, Zhi‐Jiang Zhang, Christopher J L Murray, and Theo Vos.

Development of methods or computational machinery

Emma Nichols, Akshaya Srikanth Bhagavathula, Khalil Eskandari, Mowafa Househ, Young‐Eun Kim, Man Mohan Mehndiratta, Ali H Mokdad, Christopher J L Murray, and Theo Vos.

Drafting the manuscript or revising it critically for important intellectual content,

Emma Nichols, Foad Abd‐Allah, Ahmed Abualhasan, Eman Abu‐Gharbieh, Rufus Olusola Akinyemi, Fares Alahdab, Jalal Arabloo, Amir Ashraf‐Ganjouei, Atif Amin Baig, Maciej Banach, Suzanne Lyn Barker‐Collo, Bernhard T Baune, Akshaya Srikanth Bhagavathula, Krittika Bhattacharyya, Antonio Biondi, Hermann Brenner, Sharath Burugina Nagaraja, Felix Carvalho, Luis F S Castro‐de‐Araujo, Ferrán Catalá‐López, Ester Cerin, Nicolas Cherbuin, Baye Dagnew, Daniel Diaz, Zahra Sadat Dibaji Forooshani, Abdel Douiri, Bruce B Duncan, David Edvardsson, Shaimaa I El‐Jaafary, Khalil Eskandari, Sharareh Eskandarieh, Seyed‐Mohammad Fereshtehnejad, Eduarda Fernandes, Pietro Ferrara, Irina Filip, Florian Fischer, Shilpa Gaidhane, Ahmad Ghashghaee, Yordanos Gizachew Yeshitila, Elena V Gnedovskaya, Rajeev Gupta, Samer Hamidi, Graeme J Hankey, Josep Maria Haro, Amr Hassan, Mowafa Househ, Milena D Ilic, Irena M Ilic, Seyed Sina Naghibi Irvani, Hiroyasu Iso, Ravi Prakash Jha, Himal Kandel, André Karch, Andre Pascal Kengne, Adnan Kisa, Mika Kivimäki, Hamidreza Komaki, Ai Koyanagi, Manasi Kumar, Iván Landires, Matilde Leonardi, Giancarlo Logroscino, Alan D Lopez, Stefan Lorkowski, Clement T Loy, Navid Manafi, Man Mohan Mehndiratta, Ritesh G Menezes, Atte Meretoja, Awoke Temesgen Misganaw, Bahram Mohajer, Yousef Mohammad, Farnam Mohebi, Ali H Mokdad, Stefania Mondello, Gabriele Nagel, Vinod C Nayak, Sandhya Neupane Kandel, Son Hoang Nguyen, Huong Lan Thi Nguyen, Virginia Nunez‐Samudio, Felix Akpojene Ogbo, Andrew T Olagunju, Sergej M Ostojic, Nikita Otstavnov, Stanislav S Otstavnov, Mayowa O Owolabi, Mona Pathak, Hamidreza Pazoki Toroudi, Carrie B Peterson, Hai Quang Pham, Michael R Phillips, Michael A Piradov, Sergio I Prada, Amir Radfar, Alberto Raggi, Fakher Rahim, Pradhum Ram, Juwel Rana, Vahid Rashedi, Salman Rawaf, David Laith Rawaf, Nima Rezaei, Stephen R Robinson, Michele Romoli, Perminder S Sachdev, Ramesh Sahathevan, Amirhossein Sahebkar, Mohammad Ali Sahraian, Davide Sattin, Silvia Schiavolin, Maria Inês Schmidt, Izza Shahid, Masood Ali Shaikh, Mika Shigematsu, Jae Il Shin, Tariq Jamal Siddiqi, João Pedro Silva, Jasvinder A Singh, Amin Soheili, Cassandra E I Szoeke, Bhaskar Thakur, Marcos Roberto Tovani‐Palone, Bach Xuan Tran, Ravensara S Travillian, Muhammad Shariq Usman, Marco Vacante, Diana Zuleika Velazquez, Narayanaswamy Venketasubramanian, Simone Vidale, Vasily Vlassov, Yuan‐Pang Wang, Anders Wimo, Ali Yadollahpour, Kazumasa Yamagishi, Zhi‐Jiang Zhang, Christopher J L Murray, and Theo Vos.

Management of the overall research enterprise (for example, through membership in the Scientific Council)

Lalit Dandona, Alan D Lopez, Ali H Mokdad, Christopher J L Murray, and Theo Vos
