# Supplementary material for: Global mortality from dementia: Application of a new method and results from the Global Burden of Disease Study 2019
Source: Alzheimers Dement (N Y). 2021 Jul 27;7(1):e12200. doi: 10.1002/trc2.12200 (PMC8315276; doi:10.1002/trc2.12200)

Appendix:

Table of contents

GATHER checklist, with description of compliance and location of information 2

Systematic Review Criteria 3

PRISMA Systematic Review Diagram 3

Studies on relative risk of all-cause mortality 4

References (mortality data) 18

Dementia prevalence estimation 21

Flowchart 21

Case definition 21

Input data 21

Modelling strategy 22

End-stage disease code lists 25

Bayesian meta-regression model specification 26

Bayesian meta-regression methods 27

Mixed-effects model 27

Constraints and priors 27

Trimming outliers 27

Final estimator 28

Nonlinear dose-response curves with constrained splines 29

B-splines and bases 29

Shape constraints 29

Posterior variance estimation 30

References 31

Global Burden of Disease World Regions 32

GATHER checklist, with description of compliance and location of information

#	GATHER checklist item	Description of compliance	Reference
**Objectives and funding**			
1	Define the indicators, populations, and time periods for which estimates were made.	Narrative provided in paper and appendix describing indicators, definitions, and populations	Main text (Methods) and appendix
2	List the funding sources for the work.	Funding sources listed in paper	Summary (Funding)
**Data Inputs**			
For all data inputs from multiple sources that are synthesised as part of the study:			
3	Describe how the data were identified and how the data were accessed.	Narrative description of data seeking methods provided	Main text (Methods) and appendix
4	Specify the inclusion and exclusion criteria. Identify all ad-hoc exclusions.	Narrative about inclusion and exclusion criteria by data type provided; ad hoc exclusions in cause-specific write-ups	Main text (Methods) and appendix
5	Provide information on all included data sources and their main characteristics. For each data source used, report reference information or contact name/institution, population represented, data collection method, year(s) of data collection, sex and age range, diagnostic criteria or measurement method, and sample size, as relevant.	An interactive, online data source tool that provides metadata for data sources by component, geography, cause, risk, or impairment has been developed	Online data citation tools: http://ghdx.healthdata.org/gbd-2019/data-input-sources
6	Identify and describe any categories of input data that have potentially important biases (e.g., based on characteristics listed in item 5).	Summary of known biases by cause included in appendix	Appendix
For data inputs that contribute to the analysis but were not synthesised as part of the study:			
7	Describe and give sources for any other data inputs.	Included in online data source tool	http://ghdx.healthdata.org/gbd-2019/data-input-sources
For all data inputs:			
8	Provide all data inputs in a file format from which data can be efficiently extracted (e.g., a spreadsheet as opposed to a PDF), including all relevant meta-data listed in item 5. For any data inputs that cannot be shared due to ethical or legal reasons, such as third-party ownership, provide a contact name or the name of the institution that retains the right to the data.	Downloads of input data available through online data tools (visualization/ data query, GHDx); input data not in tools will be made available upon request	Online data visualisation tools, data query tools, and the Global Health Data Exchange
**Data analysis**			
9	Provide a conceptual overview of the data analysis method. A diagram may be helpful.	Flow diagrams of the overall methods and cause-specific modelling processes have been provided	Main text (Methods) and appendix
10	Provide a detailed description of all steps of the analysis, including mathematical formulae. This description should cover, as relevant, data cleaning, data pre-processing, data adjustments and weighting of data sources, and mathematical or statistical model(s).	Flow diagrams and methods write-ups for each cause, databases, and modelling processes have been provided	Main text (Methods) and appendix
11	Describe how candidate models were evaluated and how the final model(s) were selected.	Provided in methodological write-ups	Appendix
12	Provide the results of an evaluation of model performance, if done, as well as the results of any relevant sensitivity analysis.	Provided in methodological write-ups	Appendix
13	Describe methods for calculating uncertainty of the estimates. State which sources of uncertainty were, and were not, accounted for in the uncertainty analysis.	Appendix	Appendix
14	State how analytic or statistical source code used to generate estimates can be accessed.	Appendix	http://ghdx.healthdata.org/gbd-2019/code (Link will be live upon GBD 2019 publication)
**Results and Discussion**			
15	Provide published estimates in a file format from which data can be efficiently extracted.	GBD 2019 results available through online data tools, the Global Health Data Exchange, and online data query tool	Main text, appendix, online data tools (visualization/ data query tools, GHDx)
16	Report a quantitative measure of the uncertainty of the estimates (e.g. uncertainty intervals).	Uncertainty provided with all results	Main text, appendix, online data tools (visualization/ data query tools, GHDx)

Systematic Review Criteria

The PubMed search was conducted on 08/14/2018 and the search terms used were: ((Excess mortality[Title/Abstract] OR Standardized mortality[Title/Abstract] OR survival[Title/Abstract] OR (relative risk[Title/Abstract] AND (mortality[Title/Abstract] OR death[Title/Abstract]))) AND (dementia[Title/Abstract] OR alzheimer's disease[Title/Abstract] OR "dementia"[MeSH Terms] OR "alzheimer disease"[MeSH Terms]) AND ("1980/01/01"[PDAT] : "2018/08/14"[PDAT]) NOT (animals[MeSH] NOT humans[MeSH])). All studies of less than 10 individuals or studies that focused on the basic biology of Alzheimer's disease or dementia were excluded during Title/Abstract screening. Only studies that reported on the relative risk, hazard ratio or odds ratio of all-cause mortality given an exposure of dementia (or similar condition) and included data on the mean age of the sample (or information that allowed for the calculation of mean age) were accepted. Studies on clinical cohorts or population representative samples were accepted and studies conducted solely in nursing homes were excluded.

PRISMA Systematic Review Diagram

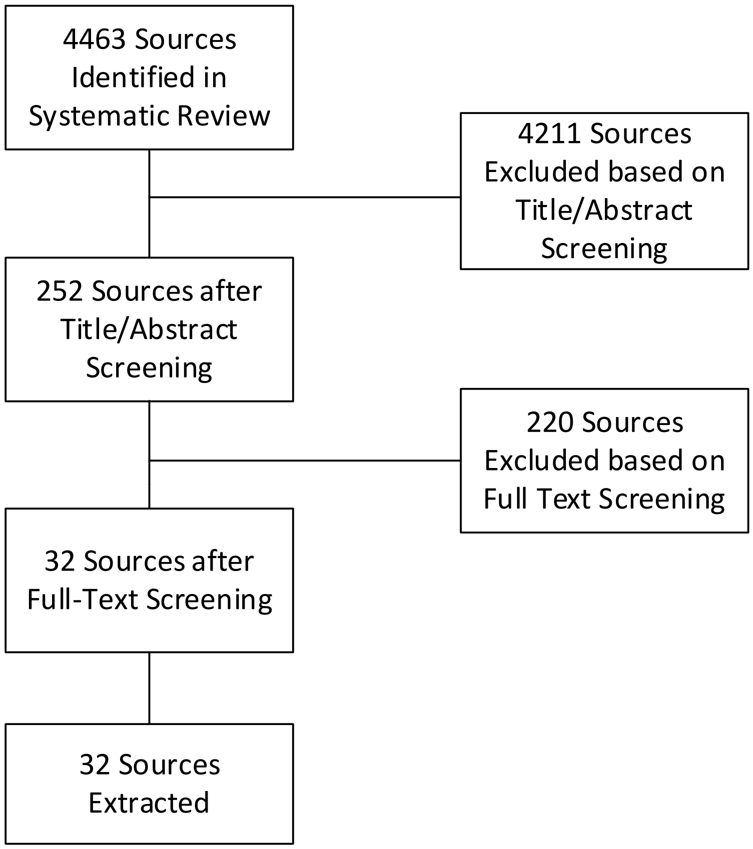

Studies on relative risk of all-cause mortality

Study	Location	Cohort	Years	Exposure(s)	Exposure ascertainment	Mortality ascertainment	Method	Control variables
**Doblhammer & Barth (2018)**[1]	Germany	AOK Public Health Insurance Population	2004 to 2013	Dementia	ICD codes identified in AOK insurance records with validation procedure to avoid overestimation.	Date of death in AOK insurance records	Cox proportional hazard model	Neurodegenerative diseases, major risk factors including diabetes, hypertension and kidney disease, cerebrovascular disease, cancer, extremity injuries, acute diseases, including pneumonia, and pulmonary embolism
**Luck et al. (2017)**[2]	Germany	German Study on Ageing, Cognition and Dementia in Primary Care Patients (AgeCoDe)	2003 to 2014	Cognitive Impairment	Individuals were assessed as cognitively impaired if the results of the Structured Interview for Diagnosis of Dementia of Alzheimer type, Multi-infarct Dementia and Dementia of other Aetiology (SIDAM) fell below age- and education-specific norms by more than 1 standard deviation.	Dates of death were obtained from relatives, GP, or from the local registry offices	Cox proportional hazard model	Age, gender, education, smoking, alcohol consumption, APOE e4 status, IADL impairment, comorbidities including hypertension, diabetes, stroke, hypercholesterolemia, hyperlipidemia, and carotid artery stenosis

Study	Location	Cohort	Years	Exposure(s)	Exposure ascertainment	Mortality ascertainment	Method	Control variables
**Georgakis et al. (2016)[3]**	Greece	Velestino Study	2005 to 2013	Mild to Moderate and Moderate to Severe Cognitive Impairment	Patients scoring between 18 and 23 were classified with mild to moderate cognitive impairment. Individuals scoring below 18 on the MMSE were classified with moderate to severe cognitive impairment.	Data were derived from death certificates in collaboration with the Local Registry Office	Cox proportional hazard model	Age, gender, educational level, social activity, family support, BMI, alcohol intake, hypertension, diabetes mellitus type 2, hypercholesterolemia, cardiovascular disease, cancer, depressive symptoms
**Naseer et al. (2016)[4]**	Sweden	Swedish National Study of Ageing and Care-Blekinge (SNAC-B)	2001 to 2003	Dementia	A single-item self-administered questionnaire was used. For subjects with dementia proxy measures were utilised.	Mortality data were collected from the population mortality register	Cox proportional hazard model	Age, physical activity, housing, cardiovascular disease, diabetes, ADL dependency
**Wu et al. (2015)[5]**	Taiwan	National Health Insurance Database	2000 to 2010	Dementia	ICD codes identified in the NHI Database, cases needed at least three outpatient clinic visits or one admission for dementia.	Deaths were identified as a withdrawal from insurance	Incidence rate ratio	None
**Paddick et al. (2015)[6]**	Tanzania	Hai Demographic Surveillance Site	2010 to 2014	Mild Cognitive Impairment and Dementia	The community screening instrument for dementia was administered and 100% of those with poor performance, 50% of those with moderate performance and 5% with good performance were more formally assessed for dementia. Dementia diagnosis was based on DSM-IV criteria and MCI diagnosis was based on	Data on mortality were collected at each follow-up visit	Cox proportional hazard model	Age, gender and education

Study	Location	Cohort	Years	Exposure(s)	Exposure ascertainment	Mortality ascertainment	Method	Control variables
					international consensus (Winblad, 2004).			
**Bahat et al. (2015)[7]**	Turkey	Geriatrics Outpatient Clinic at a University Hospital	1999 to 2010	Mild Cognitive Impairment	MMSE scores of 24 or below were considered impaired.	Mortality was assessed through the official website of the registration office	Cox proportional hazard model	Age, sex, ADL impairment, diabetes, hyperlipidemia, total number of diseases
**Meng & D'Arcy (2012)[8]**	Canada	Canadian Study of Health and Ageing	1991 to 2001	Cognitive Impairment Not Dementia and Dementia	Cognitive status was evaluated at consensus diagnosis by a neuropsychologist, specialist physician, and nurse following DSM-III-R criteria.	Not specified	Cox proportional hazard model	Age, sex, marital status, race, education, comorbidity (yes/no)
**Villarego et al. (2011)[9]**	Spain	NEDICES Cohort Study	1994 to 2007	Dementia	The screening phase included the MMSE and the Pfeffer Functional Activities Questionnaire. Individuals who screened positive were examined by trained neurologists and were diagnosed according to DSM-IV criteria.	The date of death was obtained from the National Population Register of Spain	Cox proportional hazard model	Age, sex, education, comorbidity index

Study	Location	Cohort	Years	Exposure(s)	Exposure ascertainment	Mortality ascertainment	Method	Control variables
**Beeri & Goldbourt (2011)[10]**	Israel	Israel Ischemic Heart Disease Project	1999 to 2005	Dementia	The Modified Telephone Interview for Cognitive Status was used as a screening test, and subjects with a TICS-m score of 27 or lower were assessed at their residencies by a physician and were diagnosed using DISM-IV criteria.	Mortality information was collected by matching the national ID number with the Israel National Population Registry	Cox proportional hazard model	Age, socioeconomic status, blood pressure, total cholesterol, smoking status,
**Steenland et al. (2010)[11]**	Georgia, USA	Neurology Department at Emory Wesley Woods health Center	1993 to 2006	Mild Cognitive Impairment, Lewy body dementia, Probable Alzheimer's disease, Frontotemporal dementia	Diagnoses are the clinical diagnoses at the time of last visit.	Mortality follow-up was conducted through matching patient IDs with the National Death Index	Cox proportional hazard model	Age, sex, race, education
**Lavretsky et al. (2010)[12]**	California, USA	Ischemic Vascular Dementia Program Project	1996 to 2008	Mild Cognitive Impairment and Dementia	Cognition was evaluated using a neuropsychological battery and diagnoses were made at a multidisciplinary case conference according to DSM-IV criteria.	Not specified	Cox proportional hazard model	None

Study	Location	Cohort	Years	Exposure(s)	Exposure ascertainment	Mortality ascertainment	Method	Control variables
**Wilson et al. (2009)[13]**	Illinois, USA	Chicago Health and Ageing Project	1998 to 2008	Mild Cognitive Impairment, Alzheimer's disease, other dementia	Each participant had a structured, uniform clinical evaluation and complete neurological examination and cognitive function testing. An experienced physician classified individuals with dementia and AD using the NINCDS-AD criteria. MCI was based on education-specific cutoff scores for each domain for those without a dementia diagnosis.	Death information was compiled through attempted follow-ups, obituaries in local newspapers. All deaths were also verified using death records from the National Death Index	Cox proportional hazard model	Age, sex, education, race
**Sund-Levander (2007)[14]**	Sweden	Sample of special housing for the elderly	2000 to 2003	Dementia	Diagnoses were noted on clinical medical records.	Mortality information was abstracted from death certificates	Cox proportional hazard model	Age, ADL status, BMI, Smoking, Chronic Diseases
**Ganguli (2005)[15]**	Pennsylvania, USA	The Monongahela Valley Independent Elders Survey	1987 to 2002	Alzheimer's disease	Participants who were classified as cognitively impaired at screening underwent the assessment protocol of the Consortium to Establish a Registry for Alzheimer's Disease and the University of Pittsburgh Alzheimer Disease Research Center protocol. Consensus diagnosis was used to classify patients using the NINCDS-AD criteria.	Mortality information was derived from death certificates	Cox proportional hazard model	Age, sex

Study	Location	Cohort	Years	Exposure(s)	Exposure ascertainment	Mortality ascertainment	Method	Control variables
**Nitrini (2005)[16]**	Brazil	Catanduva, Sao Paulo	1997 to 2000	Dementia	All subjects who screened positive for diagnostic evaluation completed a neuropsychological battery and the CDR scale. All data were then analysed by three neurologists who made diagnoses according to the DSM-IV criteria.	Mortality information was obtained from relatives or through the town obituary records	Cox proportional hazard model	Age, visual impairment, cardiovascular disease history
**Tschanz et al. (2004)[17]**	Utah, USA	Cache County Study on Memory and Ageing	1995 to 2001	Dementia	Screened subjects with the 3MS and IQCODE questionnaires; those flagged by screen and everyone over 90 were evaluated further. Findings were reviewed by a board-certified geriatric psychiatrist and neuropsychologist who assigned diagnoses according to DSM-III-R criteria.	Mortality information was obtained by reviewing local obituaries and from quarterly reports from the Utah Department of Vital Statistics	Cox proportional hazard model	Age, education, APOE4, asthma, cerebrovascular disease, coronary heart disease, hypercholesterolemia, hypertension, pneumonia, peptic ulcer disease, pulmonary disease, head injury, Parkinson's disease, diabetes
**Yamada et al. (2004)[18]**	Japan	Adult Health Study of Hiroshima	1992 to 1999	Alzheimer's disease and Vascular dementia	All subjects were administered cognitive function tests, neurological examinations, and informant questionnaires and DSM-III-R criteria were applied.	Deaths were confirmed thought he Japanese family registration system and information was collected from death certificates	Poisson regression analysis	Age, sex, heart disease, stroke and cancer history

Study	Location	Cohort	Years	Exposure(s)	Exposure ascertainment	Mortality ascertainment	Method	Control variables
**Noale et al. (2003)[19]**	Italy	Italian Longitudinal Study on Ageing	1992 to 1996	Dementia	Participants who scored less than 24 on the MMSE or for whom proxy respondents reported previous diagnoses were examined further. In the second phase, medical records were reviewed and participants were examined by a specialist for the diagnosis of dementia according to DSM-III-R criteria.	For individuals who had died before the follow-up assessment, death certificates were obtained from the national registry	Cox proportional hazard model	Age, sex
**Qiu et al. (2001)[20]**	Sweden	Kungsholmen Project	1987 to 1998	Dementia and Alzheimer's disease	Participants underwent neurological and physical examination as well as cognitive testing. DSM-III-R criteria were used to diagnose dementia and all diagnoses were reviewed by a specialised clinician. Diagnosis of Alzheimer's disease required gradual onset, progressive deterioration and lack of any other specific causes of dementia. For deceased subjects, clinical records, discharge diagnoses and death certificates were reviewed	Mortality was ascertained through death certificates	Cox proportional hazard model	Age, sex, MMSE baseline score, comorbidity, socioeconomic status

Study	Location	Cohort	Years	Exposure(s)	Exposure ascertainment	Mortality ascertainment	Method	Control variables
**Helmer et al. (2001)[21]**	France	Personnes Agees Quid (PAQUID)	1988 to 1998	Dementia and Alzheimer's disease	Participants who met the criteria for memory impairment, impairment of at least one other cognitive function and interference with social or professional life based on a series of psychometric tests were seen by a senior neurologist to diagnosed dementia based on DSM-II-R criteria and Alzheimer's disease based on NINCDS-ADRDA criteria.	Mortality was assessed from death certificates from the national registry of mortality statistics	Cox proportional hazard model	Age, sex, education, comorbidity index, baseline ADL dependency
**Witthaus et al. (1999)[22]**	Netherlands	The Rotterdam Study	19990 to 1992	Dementia	The MMSE and Geriatric Mental State Schedule were used to screen participants and those who screened positive underwent further testing based on the Cambridge Examination for Mental Disorders of the Elderly. Diagnoses were based on all available information and were assigned by an expert panel and based on DSM-III-R criteria.	Not specified	Mortality Rate Ratios	None

Study	Location	Cohort	Years	Exposure(s)	Exposure ascertainment	Mortality ascertainment	Method	Control variables
**Katzman et al. (1994)[23]**	China	Population Survey in Shanghai	1987 to 1992	Alzheimer's disease, Vascular dementia and other dementia	Participants were screened with the Chinese version of the MMSE and those who screened positive based on education specific cutoffs underwent an intensive evaluation. Dementia was diagnosed based on DSM-III criteria and Alzheimer's disease was diagnosed based on NINCDS-ADRDA criteria by consensus diagnosis	Mortality data for all initial participants was obtained from the Shanghai registry of vital data	Cox proportional hazard model	Age, sex, education, chronic conditions
**Beydoun et al. (2013)[24]**	Maryland, USA	Baltimore Longitudinal Study of Ageing	1958 to 2009	Mild Cognitive Impairment, dementia, Alzheimer's disease	All participants are reviewed annually if they screen positive on the Blessed Information Memory Concentration Test or the Dementia Questionnaire, or if their Clinical Dementia Rating score is 0.5 or higher using subject or informant report. Diagnoses for dementia are determined based on DSM-III-R criteria and Alzheimer's disease by NINCDS-ADRDA criteria. MCI was diagnosed when cognitive impairment was evident without any significant functional loss	All participants were followed for vital status and a consensus of three physicians determined cause and date of death using death certificates, hospital and physician records and autopsy data as available	Cox proportional hazard model	Age, sex, race, education, smoking status, BMI, APOE4

Study	Location	Cohort	Years	Exposure(s)	Exposure ascertainment	Mortality ascertainment	Method	Control variables
**James et al. (2014)[25]**	United States	Religious Orders Study and Rush Memory and Ageing Project	1994 to 2013	Dementia and Alzheimer's disease	Participants were evaluated annually, including a medical history, neurologic examination and cognitive testing. Diagnoses were the result of a three-stage process with computer scoring of cognitive tests, followed by clinical judgement by neuropsychologist and finally diagnostic classification by an experienced clinician. Diagnoses were made given the NINCDS-ADRDA criteria	Dates of death are largely known due to the high rates of autopsy. Deaths are occasionally detected during quarterly contacts with an informant, and in a small number of cases through the Social Security Death Index	Cox proportional hazard model	Age, sex, race, parent study
**Fitzpatrick et al. (2005)[26]**	United States	Cardiovascular Health Study	1992 to 2002	Dementia, Alzheimer's disease, vascular dementia	Those failing the screening with 3MSE and other parameters were invited back for detailed neuropsychological testing. A committee of neurologists and psychiatrists from all four centres evaluated data to classify dementia. The clinical definition required progressive or static deficit impairing activities of daily living, with impairments in two cognitive domains. Type of dementia was	Deaths were identified during surveillance calls, during scheduling calls or through local daily newspaper obituaries	Cox proportional hazard model	Age, sex, race

Study	Location	Cohort	Years	Exposure(s)	Exposure ascertainment	Mortality ascertainment	Method	Control variables
					classified with NINCDS-ADRDA criteria.			
**Koller et al. (2012)[27(p20)]**	Germany	Gmünder ErsatzKasse (GEK) health insurance company	2005 to 2011	Dementia	Dementia patients were identified by those who had at least one ICD-10 code for dementia in ambulatory care in at least three of four consecutive quarters.	Not specified	Cox proportional hazard model	Age, sex, level of care dependency, Elixhauser index for comorbidities
**Rait et al. (2010)[28]**	United Kingdom	The Health Improvement Network (THIN)	1990 to 2007	Dementia	All adults 60 years or over with a first ever code for dementia.	Mortality data was derived from primary care records	Conditional Poisson model	Age, sex, deprivation index, smoking, alcohol, chronic disease

Study	Location	Cohort	Years	Exposure(s)	Exposure ascertainment	Mortality ascertainment	Method	Control variables
**Guhne et al. (2006)**[29]	Germany	Leipzig Longitudinal Study of the Aged (LEILA75+)	1997 to 1999	Dementia	The Structured Interview for Diagnosis of Dementia of Alzheimer-type, Multi-infarct Dementia and Dementia of Other Etiology (SIDAM) was administered and the diagnostic algorithm was used to derive diagnoses of dementia, Alzheimer's disease and vascular dementia. Consensus conferences were held on each case and clinical diagnosis was made according to DSM-IV criteria.	Mortality data were obtained from structured proxy interviews or from the official registry office	Cox proportional hazard model	Age, sex, education, institutionalisation, and comorbidity
**Aevarsson, Savnborg & Skoog (1998)**[30]	Sweden	Longitudinal Gerontological and Geriatric Population Studies	1985 to 1993	Dementia and Alzheimer's disease	Diagnosis of dementia was based on clinical and neuropsychiatric examination and informant interview using DSM-III-R criteria. Alzheimer's disease was diagnosed based on NINCDS-ADRDA criteria	Mortality data was available from the census register	Cox proportional hazard model	Age, sex, lung disease, cancer, hypertension, myocardial infarction, cerebrovascular disease, congestive heart failure

Study	Location	Cohort	Years	Exposure(s)	Exposure ascertainment	Mortality ascertainment	Method	Control variables
**Cruz-Oliver et al. (2012)[31]**	Missouri, USA	Geriatric Research, Education and Clinical Center Veterans Affairs Hospital	2003 to 2011	Mild Cognitive Impairment	Participants were considered impaired if they scored 24 or lower on the MMSE.	Vital status was determined by the presence of a date of death listed in the electronic medical record. Death was confirmed through the review of death certificates	Cox proportional hazard model	Age, number of anticholinergic medications, and comorbidities
**Baldereschi et al. (1999)[32]**	Italy	Italian Longitudinal Study on Ageing	1992 to 1995	Dementia	Those who score less than 24 on the MMSE were invited for further examination. Participants were then diagnosed according the DSM-III-R criteria and doubtful cases were extensively reviewed by a panel of senior clinicians.	Participants were followed up by telephone interview to determine vital status and death certificates were collected for each individual who died	Cox proportional hazard model	Age, sex, institutionalisation, education, chronic conditions
**Tsuji et al. (1995)[33]**	Japan	Sendai Longitudinal Study of Ageing	1988 to 1991	Dementia	Those who screened positive were invited for a physical and psychiatric examination by the trained public health nurses. Based on information collected, diagnostic evaluation was made according to DSM-III-R criteria by a committee of psychiatrists.	Deaths were identified through the residents registration card and verified by death certificates	Cox proportional hazard model	Age, sex

Study	Location	Cohort	Years	Exposure(s)	Exposure ascertainment	Mortality ascertainment	Method	Control variables
**Matsui et al. (2009)[34]**	Japan	Hisayama Study	1985 to 2002	Dementia	Subjects who tested below the cutoff scores of either the Hasegawa dementia scale, Hasegawa revised dementia scale or the MMSE, patients were invited for comprehensive investigations, including interviews of the families or attending physicians, physical and neurological exams and a review of clinical records. Diagnosis of dementia was based on DSM-III-R criteria.	Not specified	Cox proportional hazard model	Age, sex

References (mortality data)

1. Doblhammer G, Barth A. Prevalence of Morbidity at Extreme Old Age in Germany: An Observational Study Using Health Claims Data. *Journal of the American Geriatrics Society*. 2018;66(7):1262-1268. doi:10.1111/jgs.15460

2. Luck T, Riedel-Heller SG, Roehr S, et al. Mortality in Incident Cognitive Impairment: Results of the Prospective AgeCoDe Study. *Journal of the American Geriatrics Society*. 2017;65(4):738-746. doi:10.1111/jgs.14666

3. Georgakis MK, Protogerou AD, Kalogirou EI, et al. Blood Pressure and All-Cause Mortality by Level of Cognitive Function in the Elderly: Results From a Population-Based Study in Rural Greece. *The Journal of Clinical Hypertension*. 2017;19(2):161-169. doi:10.1111/jch.12880

4. Naseer M, Forssell H, Fagerström C. Malnutrition, functional ability and mortality among older people aged ⩾60 years: a 7-year longitudinal study. *European Journal of Clinical Nutrition*. 2016;70(3):399-404. doi:10.1038/ejcn.2015.196

5. Wu C-Y, Hu H-Y, Chow L-H, et al. The Effects of Anti-Dementia and Nootropic Treatments on the Mortality of Patients with Dementia: A Population-Based Cohort Study in Taiwan. *PLoS One*. 2015;10(6). doi:10.1371/journal.pone.0130993

6. Paddick S-M, Kisoli A, Dotchin CL, et al. Mortality rates in community-dwelling Tanzanians with dementia and mild cognitive impairment: a 4-year follow-up study. *Age Ageing*. 2015;44(4):636-641. doi:10.1093/ageing/afv048

7. Bahat G, Tufan F, Bahat Z, et al. Observational cohort study on correlates of mortality in older community-dwelling outpatients: The value of functional assessment. *Geriatrics & Gerontology International*. 2015;15(11):1219-1226. doi:10.1111/ggi.12422

8. Meng X, D’Arcy C. Mortality and morbidity hazards associated with cognitive status in seniors: A Canadian population prospective cohort study. *Asia-Pacific Psychiatry*. 2013;5(3):175-182. doi:10.1111/j.1758-5872.2012.00222.x

9. Villarejo A, Benito-León J, Trincado R, et al. Dementia-Associated Mortality at Thirteen Years in the NEDICES Cohort Study. *Journal of Alzheimer’s Disease*. 2011;26(3):543-551. doi:10.3233/JAD-2011-110443

10. Beeri MS, Goldbourt U. Late-Life Dementia Predicts Mortality Beyond Established Midlife Risk Factors. *The American Journal of Geriatric Psychiatry*. 2011;19(1):79-87. doi:10.1097/JGP.0b013e3181e043d0

11. Steenland K, MacNeil J, Seals R, Levey A. Factors Affecting Survival of Patients with Neurodegenerative Disease. *NED*. 2010;35(1):28-35. doi:10.1159/000306055

12. Lavretsky H, Zheng L, Weiner MW, et al. Association of Depressed Mood and Mortality in Older Adults With and Without Cognitive Impairment in a Prospective Naturalistic Study. *AJP*. 2010;167(5):589-597. doi:10.1176/appi.ajp.2009.09020280

13. Wilson RS, Aggarwal NT, Barnes LL, Bienias JL, Leon CFM de, Evans DA. Biracial Population Study of Mortality in Mild Cognitive Impairment and Alzheimer Disease. *Arch Neurol*. 2009;66(6):767-772. doi:10.1001/archneurol.2009.80

14. Sund-Levander M, Grodzinsky E, Wahren LK. Gender differences in predictors of survival in elderly nursing-home residents: a 3-year follow up. *Scandinavian Journal of Caring Sciences*. 2007;21(1):18-24. doi:10.1111/j.1471-6712.2007.00431.x

15. Ganguli M, Dodge HH, Shen C, Pandav RS, DeKosky ST. Alzheimer Disease and Mortality: A 15-Year Epidemiological Study. *Arch Neurol*. 2005;62(5):779-784. doi:10.1001/archneur.62.5.779

16. Nitrini R, Caramelli P, Herrera E, et al. Mortality from dementia in a community-dwelling Brazilian population. *International Journal of Geriatric Psychiatry*. 2005;20(3):247-253. doi:10.1002/gps.1274

17. Tschanz JT, Corcoran C, Skoog I, et al. Dementia: the leading predictor of death in a defined elderly population: the Cache County Study. *Neurology*. 2004;62(7):1156-1162. doi:10.1212/01.wnl.0000118210.12660.c2

18. Yamada M, Kasagi F, Sasaki H, Mimori Y, Suzuki G. Effects of Dementia on Mortality in the Radiation Effects Research Foundation Adult Health Study. *GER*. 2004;50(2):110-112. doi:10.1159/000075562

19. Noale M, Maggi S, Minicuci N, et al. Dementia and Disability: Impact on Mortality. *DEM*. 2003;16(1):7-14. doi:10.1159/000069987

20. Qiu C, Bäckman L, Winblad B, Agüero-Torres H, Fratiglioni L. The Influence of Education on Clinically Diagnosed Dementia Incidence and Mortality Data From the Kungsholmen Project. *Arch Neurol*. 2001;58(12):2034-2039. doi:10.1001/archneur.58.12.2034

21. Helmer C, Joly P, Letenneur L, Commenges D, Dartigues J-F. Mortality with Dementia: Results from a French Prospective Community-based Cohort. *Am J Epidemiol*. 2001;154(7):642-648. doi:10.1093/aje/154.7.642

22. Witthaus E, Ott A, Barendregt JJ, Breteler M, Bonneux L. Burden of mortality and morbidity from dementia. *Alzheimer Dis Assoc Disord*. 1999;13(3):176-181. doi:10.1097/00002093-199907000-00011

23. Katzman R, Hill LR, Yu ESH, et al. The Malignancy of Dementia: Predictors of Mortality in Clinically Diagnosed Dementia in a Population Survey of Shanghai, China. *Arch Neurol*. 1994;51(12):1220-1225. doi:10.1001/archneur.1994.00540240064017

24. Beydoun MA, Beydoun HA, Kaufman JS, et al. Apolipoprotein E ε4 Allele Interacts with Sex and Cognitive Status to Influence All-Cause and Cause-Specific Mortality in U.S. Older Adults. *Journal of the American Geriatrics Society*. 2013;61(4):525-534. doi:10.1111/jgs.12156

25. James BD, Leurgans SE, Hebert LE, Scherr PA, Yaffe K, Bennett DA. Contribution of Alzheimer disease to mortality in the United States. *Neurology*. 2014;82(12):1045-1050. doi:10.1212/WNL.0000000000000240

26. Fitzpatrick AL, Kuller LH, Lopez OL, Kawas CH, Jagust W. Survival following dementia onset: Alzheimer's disease and vascular dementia. *Journal of the Neurological Sciences*. 2005;229-230:43-49. doi:10.1016/j.jns.2004.11.022

27. Koller D, Kaduszkiewicz H, Bussche H van den, et al. Survival in patients with incident dementia compared with a control group: a five-year follow-up. *International Psychogeriatrics*. 2012;24(9):1522-1530. doi:10.1017/S1041610212000361

28. Rait G, Walters K, Bottomley C, Petersen I, Iliffe S, Nazareth I. Survival of people with clinical diagnosis of dementia in primary care: cohort study. *BMJ*. 2010;341. doi:10.1136/bmj.c3584

29. Gühne U, Matschinger H, Angermeyer MC, Riedel-Heller SG. Incident Dementia Cases and Mortality. *DEM*. 2006;22(3):185-193. doi:10.1159/000094786

30. Aevarsson Ó, Svanborg A, Skoog I. Seven-Year Survival Rate After Age 85 Years: Relation to Alzheimer Disease and Vascular Dementia. *Arch Neurol*. 1998;55(9):1226-1232. doi:10-1001/pubs.Arch Neurol.-ISSN-0003-9942-55-9-noc7310

31. Cruz-Oliver DM, Malmstrom TK, Allen CM, Tumosa N, Morley JE. The veterans affairs Saint Louis University mental status exam (slums exam) and the mini-mental status exam as predictors of mortality and institutionalization. *J Nutr Health Aging*. 2012;16(7):636-641. doi:10.1007/s12603-012-0098-9

32. Baldereschi M, Di Carlo A, Maggi S, et al. Dementia is a major predictor of death among the Italian elderly. ILSA Working Group. Italian Longitudinal Study on Aging. *Neurology*. 1999;52(4):709-713. doi:10.1212/wnl.52.4.709

33. Tsuji I, Minami Y, Li J-H, et al. Dementia and Physical Disability as Competing Risks for Mortality in a Community-Based Sample of the Elderly Japanese. *The Tohoku Journal of Experimental Medicine*. 1995;176(2):99-107. doi:10.1620/tjem.176.99

34. Matsui Y, Tanizaki Y, Arima H, et al. Incidence and survival of dementia in a general population of Japanese elderly: the Hisayama study. *Journal of Neurology, Neurosurgery & Psychiatry*. 2009;80(4):366-370. doi:10.1136/jnnp.2008.155481

Dementia prevalence estimation

(Reprinted from Global burden of 369 diseases, injuries, and impairments, 1990–2019: a systematic analysis for the Global Burden of Disease Study 2019" by GBD 2019 Diseases, Injuries, and Impairments Collaborators, in press)

Flowchart

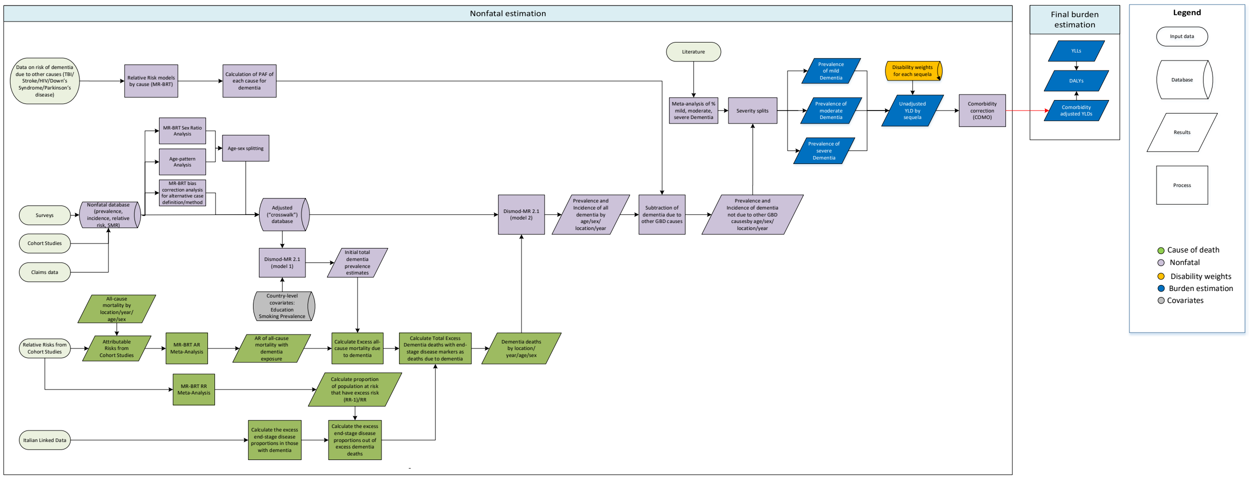

Case definition

Dementia is a progressive, degenerative, and chronic neurological disorder typified by memory impairment and other neurological dysfunctions. For the purposes of GBD 2019, we use the Diagnostic and Statistical Manual of Mental Disorders III, IV or V, or ICD case definitions as the reference. The DSM-IV definition is:

- Multiple cognitive deficits manifested by both memory impairment and one of the following: aphasia, apraxia, agnosia, disturbance in executive functioning
- Must cause significant impairment in occupational functioning and represent a significant decline
- Course is characterised by gradual onset and continuing cognitive decline
- Cognitive deficits are not due to other psychiatric conditions
- Deficits do not occur exclusively during the course of a delirium

A wide array of diagnostic and screening instruments exists, including Clinical Dementia Rating scale (CDR), Mini Mental State Examination (MMSE), and the Geriatric Mental State (GMS). For severity rating purposes we use the CDR as the reference. The relevant ICD-10 codes for dementia are F00, F01, F02, F03, G30, and G31. The ICD-9 codes are 290, 291.2, 291.8, 294, and 331.

Unlike most causes in the Global Burden of Disease project, dementia mortality and morbidity estimates are modelled jointly. This is because of marked discrepancies between prevalence data and cause of death data. Specifically, prevalence data suggest little to no variation over time (eg, 1990–2019), whereas age-standardised mortality rates in vital registrations in high-income countries have increased multiple times over this same period. Additionally, prevalence variation between countries is much smaller than the variation in death rates assigned to dementia in vital registration. We attribute these discrepancies to changing coding practices rather than epidemiological change.

Because of this joint procedure, descriptions of the mortality estimation process are included where relevant.

Input data

Model inputs

To inform our estimates of burden due to dementia, we use mortality data from vital registration systems, as well as prevalence data from surveys and administrative data such as claims sources. All data sources utilized in this

analysis (including data on excess mortality, prevalence, incidence etc.) are available from: http://ghdx.healthdata.org/gbd-2019/data-input-sources.

Item response theory for prevalence prediction

The prevalence models for dementia are data sparse, and there aren't many surveys done in low-income settings. However, there is a larger body of surveys that collect data on cognitive tests and functional limitations which are the two main components of a DSM or ICD diagnosis. Predictions of dementia prevalence using information from these questions would allow for expanded data coverage and additional information in locations where there are currently no data guiding estimates.

Generating these predictions requires calibrating a model to samples that have information about both functional limitations, cognition, and adjudicated dementia diagnoses. However, making comparisons across surveys can be difficult, as each survey asks a different set of questions about cognition and limitations, although there is some overlap. This overlap allows for the use of item response theory methods for the harmonisation of these scales. Once the scales are harmonised the subsamples can be utilised to create a model for the prediction of prevalence.

In GBD 2019, data from the ADAMS and HRS surveys were extracted and used for item response theory modelling to estimate prevalence. HRS is a nationally representative survey in the US, which has data on cognition and functional limitations. ADAMS is a subsample of HRS that includes much more detailed neuropsychological testing and adjudicated dementia diagnoses. ADAMS includes almost all questions in HRS plus additional questions as well.

Excluding incidence

Since 2016, we have made the decision to exclude incidence data, because in locations with high quality cohort data on prevalence and incidence, the two are not compatible (incidence data implies a higher prevalence than what is reported). Because dementia has a slow, insidious onset and prevalence is easier to measure, we trust prevalence data more and rely on this, excluding incidence data from DisMod.

Modelling strategy

First, prevalence data was sex split, crosswalked, and age split. Studies with age and sex detail separately were split into age- and sex-specific datapoints. Data specified as "both" sex data were split into male- and female-specific datapoints using MR-BRT to get a model ratio of female/male prevalence and then using the following equations:

Male prevalence:

$$prev_{male} = prev_{both} * \frac{pop_{both}}{(pop_{male} + ratio * pop_{female})}$$

Female prevalence:

$$prev_{female} = ratio * prev_{male}$$

We also split datapoints where the age range was greater than 25 years using the global age pattern.

Dementia studies are heterogeneous. Even with a smaller number of definitions (DSM/ICD), there are a large number of different ways to diagnose dementia. For example, out of 272 sources used in GBD 2017, there were 263 different methods of diagnosing dementia (overlap was among those who used 10/66 protocol or AGECAT algorithm). Most use a two-step procedure, where you screen using a cognitive test and then only fully evaluate those that fall below a certain pre-defined threshold. We controlled for methods differences by crosswalking alternative case definitions to reference. Study covariates are based on broad categories determined after going through the diagnostic heterogeneity and there are some added for specific criteria that we know are biased. The same study-level covariates were used in 2019 as in 2017 with the addition of item response theory HRS predictions.

Crosswalking was carried out using a logit difference network meta-regression analysis. U.S. Marketscan were separately crosswalked to standardise the claims data relative to existing literature data.

MR-BRT crosswalk adjustment factors for dementia (network analysis)

Data input	Reference or alternative case definition	Gamma	Beta Coefficient, Logit (95% CI)	Adjustment factor*
DSM or ICD case definition	Ref	0.34	---	---
Clinical records diagnosis criteria	Alt		-0.05 (-0.72 – 0.61)	0.51
Algorithm diagnosis criteria (AGECAT)	Alt		0.08 (-0.59 – 0.74)	0.50
U.S. Marketscan	Alt		-0.95 (-1.61 – -0.28)	0.50
NIA-AA diagnosis criteria	Alt		0.51 (-0.16 – 1.17)	0.53
10/66 algorithm diagnosis criteria	Alt		0.97 (0.30 – 1.64)	0.50
GP records used for diagnosis	Alt		-1.21 (-1.88 – -0.54)	

A separate analysis was conducted to crosswalk Marketscan claims data (excluding Marketscan year 2000) to non-claims data using a spline on age. The plot below shows the model fit over different ages (gamma = 0.07).

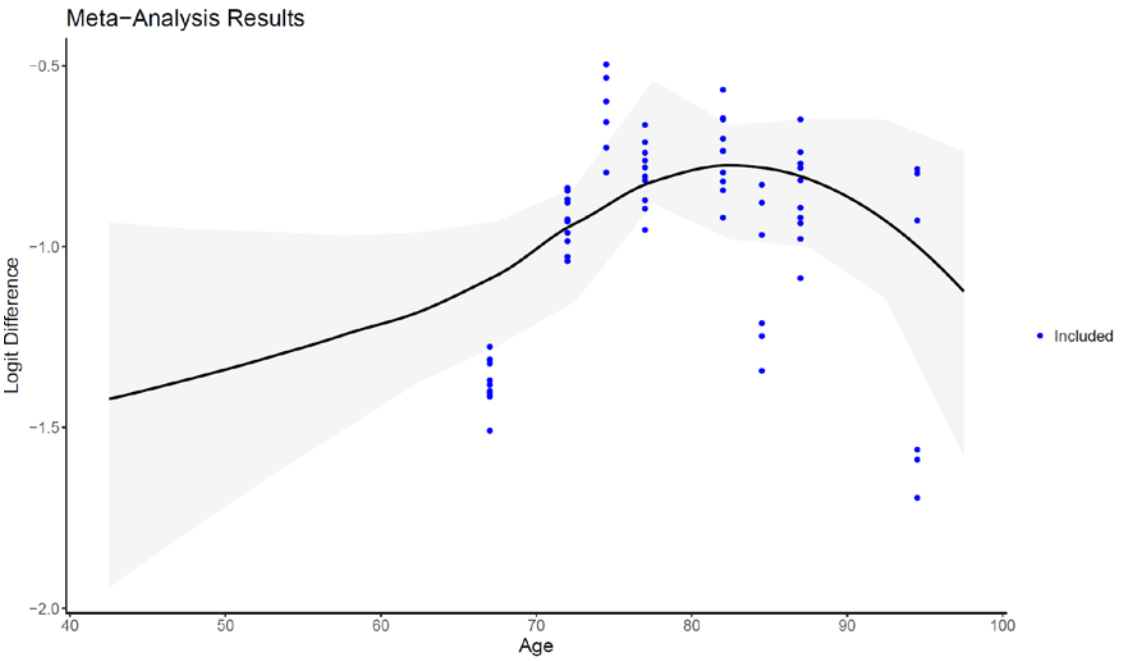

Two country-level covariates were included in the initial Dismod model. Age-standardised education was used as a proxy for general brain health/use that may be protective of dementia – specifically Alzheimer's disease. Smoking prevalence (age-standardised, both sexes) was also used as a covariate to guide estimates, as the literature has shown a positive relationship between smoking and dementia.

Note that two Dismod models were run with prevalence inputs – the first uses adjusted prevalence data (Dismod Model 1 in flowchart), which accounts for dementia caused by other diseases. The second uses unadjusted dementia

(Dismod Model 2 in flowchart) which accounts for all dementia regardless of cause (this is the dementia impairment envelope). The tables below summarise country-level covariates used in each of these Dismod model.

Covariates. Summary of covariates used in the Dementia DisMod-MR meta-regression model (adjusted prevalence, Model 1)

Covariate	Type	Exponentiated beta (95% Uncertainty Interval)
Smoking prevalence (age-standardised)	Prevalence	2.71 (1.03 — 7.36)
Educational attainment (age-standardised)	Prevalence	0.92 (0.92 — 0.92)

Covariates. Summary of covariates used in the Dementia DisMod-MR meta-regression model (unadjusted prevalence, Model 2)

Covariate	Type	Exponentiated beta (95% Uncertainty Interval)
Smoking prevalence (age-standardised)	Prevalence	1.00 (1.00 — 1.01)
Educational attainment (age-standardised)	Prevalence	0.92 (0.92 — 0.92)

Exclusion of dementia due to other GBD causes

While the DSM definition excludes dementia cases, where the syndrome is caused by other psychiatric disorders, it does not exclude dementia cases caused by other diseases, not included in DSM. This includes, stroke, Parkinson's disease, Down's syndrome and traumatic brain injury (TBI), which are found elsewhere in the GBD cause list. To prevent double counting of prevalent cases, both under dementia and each of these other causes, we adjusted our dementia prevalence to exclude cases caused by these other conditions. To do so, in GBD 2019 we used data from the Aging, Demographics and Memory study (ADAMS), to estimate the relative risk of getting dementia for each condition included in the ADAMS dataset (stroke, Parkinson's disease, TBI). We then conducted more extensive systematic reviews on all five of these conditions to model each separately. Relative risk models were run using MR-BRT, and population attributable fractions (PAF) for each condition were calculated with the following equation, where exposure is defined as the prevalence of condition:

$$PAF = \frac{exposure * (RR - 1)}{[exposure * (RR - 1)] + 1}$$

As mentioned previously, the estimation of morbidity due to dementia occurs in conjunction with the mortality estimation. Additional details on this process can be found in the COD capstone appendix.

End-stage disease code lists

Decubitus:

ICD-9: 707.0/2/8/9, ICD-10: L89 (all)

Malnutrition:

ICD-9: 262 (all), 263 (all), 261 (all), ICD-10: E41 (all), E43 (all), E44 (all), E46 (all)

Pneumonia:

ICD-9: 507.0, 514 (all), 482 (all), 483 (all), 486 (all), 485, ICD-10: J69 (all), J18 (all), J15 (all)

Sepsis:

ICD-9: 038 (all), ICD-10: A40 (all), A41 (all)

Fall from Bed:

ICD-9: E884.4, ICD-10: W06 (all)

UTI:

ICD-9: 599.0, 590.1 (all), 595, 595.0 ICD-10: N10 (all), N30, N30.0, N39.0

Senility:

ICD-9: 797 (all), ICD-10: R41.81, R54 (all)

Dehydration:

ICD-9: 276.5 (all), ICD-10: E86 (all)

Sodium Imbalance:

ICD-9: 276.0/1/8/9, ICD-10: E87.0/1/6/8

Muscular Wasting:

ICD-9: 728.2, ICD-10: M62.5 (all)

Bronchitis:

ICD-9: 466 (all), ICD-10: J20 (all)

Dysphagia:

ICD-9: 787.2 (all), ICD-10: R13 (all)

Hip Fracture:

ICD-9: 820 (all), ICD-10: S72.0 (all), S72.1 (all), S72.2 (all)

Bedridden:

ICD-9: V4984, ICD-10: Z74.0 (all)

Bayesian meta-regression model specification

Meta-regression on Attributable Risk

We included covariates on the exposure category of the study (all dementia, Alzheimer's disease, cognitive impairment), whether study was conducted in a clinical setting, five indicator variables for what each study controlled for (educational attainment, basic cardiovascular disease comorbidities such as stroke and heart disease, more extensive cardiovascular disease factors such as blood pressure and cholesterol, and smoking and alcohol consumption, 'over-control' category). These study characteristics were included as covariates on the mean effect and the variance to account for the effects of these study traits on the mean and variance of reported estimates. We tested a covariate on sex, but as this was not statistically significant, we did not retain sex in the final model. We included a spline on age with four knots, placed at the quintiles of data density. We included Gaussian priors with a mean of 0 on the covariates included in the model to ensure that covariate effect estimates were not over-influenced by sparse data.

Meta-regression on Relative Risk

The same settings were used in the model for relative risk, with the exception of an addition of Gaussian priors of mean 0 on the slope of the tail segments of the spline to reduce the flexibility of the spline in segments with more sparse data.

Bayesian meta-regression methods

(Reprinted from Global burden of 369 diseases, injuries, and impairments, 1990–2019: a systematic analysis for the Global Burden of Disease Study 2019" by GBD 2019 Diseases, Injuries, and Impairments Collaborators, in press)

This section details the statistical models underlying MR-BRT, and the fitting procedure used to obtain estimates. Further details on models and algorithms can be found in the technical report.1

The MR-BRT program is a set of wrappers customised for global health problems that use the open source mixed effects package LimeTr (https://github.com/zhengp0/limetr).1 We describe the basic functionality in the sections below.

Mixed-effects model

We consider the following nonlinear mixed-effects model:

$$\boldsymbol{y}_i = \mathbf{F}_i(\beta) + \mathbf{Z}_i \boldsymbol{u}_i + \boldsymbol{\epsilon}_i$$
$$\boldsymbol{u}_i \sim N(\mathbf{0}, \boldsymbol{\Gamma}),\ \boldsymbol{\Gamma} = \mathrm{diag}(\gamma), \qquad \boldsymbol{\epsilon}_i \sim N(\mathbf{0}, \boldsymbol{\Lambda}), \tag{1}$$

where $y_i \in \mathbb{R}^{n_i}$ is the vector of observations from the ith study, $\varepsilon_i \in \mathbb{R}^{n_i}$ are measurement errors with given covariance $\boldsymbol{\Lambda}, \boldsymbol{u}_i\ y_i \in \mathbb{R}^{k_y}$ are independent random effects, and $\boldsymbol{Z}_i \in \mathbb{R}^{n_i + k_y}$ is a linear map, and β are regression coefficients. The models F_i may be nonlinear.

To fit (β, γ) we solve the marginal likelihood problem:

$$\min_{\beta,\gamma} f(\beta,\gamma) := \sum_{i=1}^{m} \frac{1}{2}(y_i - \mathbf{F}_i(\beta))^{\top} \left(\mathbf{Z}_i \boldsymbol{\Gamma} \mathbf{Z}_i^{\top} + \boldsymbol{\Lambda}_i\right)^{-1} (\boldsymbol{y}_i - \mathbf{F}_i(\beta)) + \frac{1}{2} ln\left|\mathbf{Z}_i \boldsymbol{\Gamma} \mathbf{Z}_i^{\top} + \boldsymbol{\Lambda}_i\right|. \tag{2}$$

When the model is linear, we can write:

$$\mathbf{F}_i(\beta) = \boldsymbol{X}\beta. \tag{3}$$

Constraints and priors

The ML estimate can be extended to incorporate nonlinear inequality constraints

$$\mathbf{C}(\boldsymbol{\theta}) \leq c,$$

where $\boldsymbol{\theta} = (\beta, \gamma)$. Constraints play a key role for polynomial splines.

It is also essential to allow priors on parameters of interest. We assume that priors are given by a functional form

$$\boldsymbol{\theta} \sim \exp(-\rho(\boldsymbol{\theta}))$$

The likelihood problem is then augmented by adding the term $(\boldsymbol{\theta})$ to the ML objective. The function ρ may be nonlinear and nonconvex, but we assume it is smooth.

Trimming outliers

Least trimmed squares (LTS) is a robust estimator2,3 for the standard regression problem. Given the problem

$$\min_{\beta} \sum_{i=1}^{n} \frac{1}{2} (y_i - \langle \boldsymbol{X}_i, \beta \rangle)^2, \tag{4}$$

the LTS estimator minimises the sum of *smallest h* residuals rather than all residuals. These estimators were initially introduced to develop linear regression estimators that have a high breakdown point (in this case 50%) and good statistical efficiency (in this case $n^{-1/2}$). Breakdown refers to the percentage of outlying points which can be added to a dataset before the resulting M-estimator can change in an unbounded way. Here, outliers can affect both the outcomes and training data (features).

LTS estimators are robust against outliers, and arbitrarily large deviations that are trimmed do not affect the final β.

Rather than writing the objective in terms of order statistics, it is far simpler to extend the likelihood using an auxiliary variable $\boldsymbol{W}$:

$$\min_{\beta,\mathbf{W}} \sum_{i=1}^{n} w_i \left(\frac{1}{2}\left(y_i - \langle \mathbf{X}_i, \beta\rangle\right)\right)^2 \quad \text{s.t.} \quad \mathbf{1}^\top \boldsymbol{W} = h, \quad \mathbf{0} \le \boldsymbol{W} \le \mathbf{1}. \tag{5}$$

The set

$$\Delta_h := \{\mathbf{W} : \mathbf{1}^\top \mathbf{W} = h, \quad \mathbf{0} \le \mathbf{W} \le \mathbf{1}\} \tag{6}$$

is known as the *capped simplex*, since it is the intersection of the h-simplex with the unit box.2 For a fixed $\beta\beta$, the optimal solution of (5) with respect to $\mathbf{W}$ assigns weight 1 to each of the smallest h residuals, and 0 to the rest. Problem (5) is solved *jointly* in (β, $\mathbf{W}$), simultaneously finding the regression estimate and classifying the observations into inliers and outliers. This joint strategy makes LTS different from post-hoc analysis, where a model is fit first with all data, and then outliers are detected using that estimate.

To explain how trimming enters the marginal likelihood problem, we focus on a single group term from the ML likelihood (2):

$$\left(\frac{1}{2}\left(\boldsymbol{y}_i - \mathbf{F}_{i(\beta)}\right)^\top \left(\mathbf{Z}_i \boldsymbol{\Gamma}^{-1} \mathbf{Z}_i^\top + \boldsymbol{\Lambda}_i\right)^{-1} \left(\boldsymbol{y}_i - \mathbf{F}_i(\beta)\right) + \frac{1}{2} \ln |\mathbf{Z}_i \boldsymbol{\Gamma}^{-1} \mathbf{Z}_i^\top + \boldsymbol{\Lambda}_i|\right)$$

We introduce auxiliary variables $\boldsymbol{W}_i \in \mathbb{R}^{n_i}$, and define

$$\boldsymbol{r}_i := y_i - \mathbf{F}_i(\beta), \quad \mathbf{W}_i := \operatorname{diag}(\mathbf{W}_i), \quad \sqrt{\mathbf{W}_i} := \operatorname{diag}\left(\sqrt{\mathbf{W}_i}\right).$$

We now form the objective

$$\frac{1}{2} \boldsymbol{r}_I^\top \sqrt{\mathbf{W}_i} \left(\sqrt{\mathbf{W}_i} \mathbf{Z}_i \boldsymbol{\Gamma}^{-1} \mathbf{Z}_i^\top \sqrt{\mathbf{W}_i} + \boldsymbol{\Lambda}_i^{\odot \mathbf{W}_i}\right)^{-1} \sqrt{\mathbf{W}_i}\, \boldsymbol{r}_i + \frac{1}{2} \ln \left| \sqrt{\mathbf{W}_i}\, \mathbf{Z}_i \boldsymbol{\Gamma}^{-1} \mathbf{Z}_i^\top \sqrt{\mathbf{W}_i} + \boldsymbol{\Lambda}_i^{\odot \mathbf{W}_i} \right|, \tag{7}$$

where $\odot$ denotes the elementwise power operation:

$$\boldsymbol{\Lambda}_i^{\odot \mathbf{W}_i} := \begin{bmatrix} \left(\lambda_{1j}\right)^{w_{i1}} & 0 & \cdots & 0 \\ 0 & \ddots & \ddots & \vdots \\ 0 & \cdots & 0 & \left(\lambda_{in_i}\right)^{w_{in_i}} \end{bmatrix} \tag{8}$$

When wij = 1, we recover the contribution of the $i$jth observation to the original likelihood. As wwij ↓ 0, the $i$jth contribution to the residual is correctly eliminated by $\sqrt{w_{ij}} \downarrow 0$. The jth row and column of $\sqrt{\boldsymbol{W}_i} \boldsymbol{Z}_i \boldsymbol{\Gamma}^{-1} \boldsymbol{Z}_i^\top \sqrt{\boldsymbol{W}_i}$ both go to 0, while the jth entry of $\boldsymbol{\Lambda} i \odot$ goes to 1, which effectively removes all impact of the jth point on the covariance matrix.

For full details and analysis, please see the technical report.1

Final estimator

Putting together the trimmed ML with priors and constraints, we arrive at the following estimator.

$$\begin{aligned} \min_{\beta,\gamma,\mathbf{W}} f(\beta,\gamma,\mathbf{W}) := \sum_{i=1}^{m} \frac{1}{2} r_i^\top \sqrt{\mathbf{W}_i} \left(\sqrt{\mathbf{W}_i}\, \mathbf{Z}_i \boldsymbol{\Gamma}^{-1} \mathbf{Z}_i^\top \sqrt{\mathbf{W}_i} + \boldsymbol{\Lambda}_i^{\odot \mathbf{W}_i}\right)^{-1} \\ \sqrt{\mathbf{W}_i}\, r_i + \frac{1}{2} \ln |\sqrt{\mathbf{W}_i}\, \mathbf{Z}_i \boldsymbol{\Gamma}^{-1} \mathbf{Z}_i^\top \sqrt{\mathbf{W}_i} + \boldsymbol{\Lambda}_i^{\odot \mathbf{W}_i}| + \rho(\beta,\gamma,\boldsymbol{\Lambda}) \\ \text{s.t.} \quad \boldsymbol{r}_i = \boldsymbol{y}_i - \mathbf{F}_i(\beta), \quad \mathbf{1}^\top \mathbf{W} = h, \quad 0 \le \mathbf{W} \le 1, \quad \boldsymbol{C}\begin{pmatrix}\beta \\ \gamma\end{pmatrix} \le c\,. \end{aligned} \tag{9}$$

The fit is obtained using iterative optimisation techniques. Problem (9) is nonlinear and non-smooth, and the optimisation is implemented in the LimeTR package4 (https://github.com/zhengp0), and relies on the IPopt interior point method.5

Nonlinear dose-response curves with constrained splines

In this section we discuss spline models for dose-response relationships. General background on splines and spline regression are available elsewhere.6,7

B-splines and bases

A spline basis is a set of piecewise polynomial functions with designated degree and domain. If we denote polynomial order by p, and the number of knots by k, we need p+k basis elements sjp, which can be generated recursively as illustrated in Figure A.

Figure A. Recursive generation of b-spline basis elements (orders 0, 1, 2)

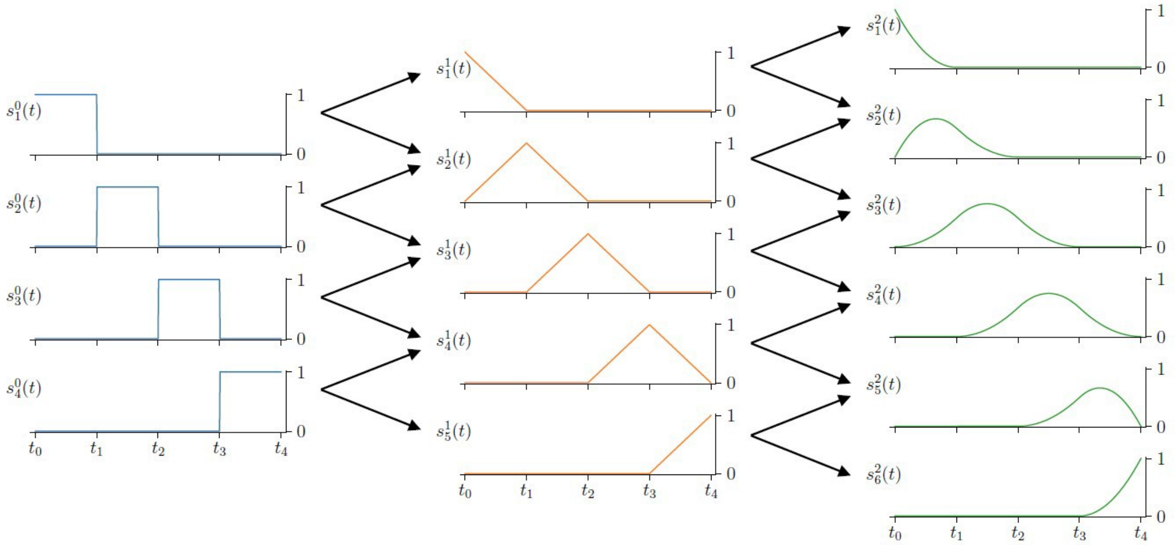

Given such a basis, we can represent any dose-response relationship as the linear combination of the spline basis elements, with coefficients $\beta \in \mathbb{R}^{p+k}$:

$$f(t) = \sum_{j=1}^{p+k} \beta_j^p s_j^p(t). \tag{10}$$

These coefficients are then inferred as part of the general estimator (9) as discussed in the previous section. An explicit representation of (11) is obtained by building a design matrix $\boldsymbol{X}$. Given a set of t values at which we have data, the jth column of $\mathbf{X}$ is given by the expression

$$\mathbf{X}_{\cdot,j} = \begin{bmatrix} s_j^p(t_0) \\ \vdots \\ s_j^p(t_k) \end{bmatrix}. \tag{11}$$

The model for direct observations data coming from (11) can now be written compactly as

$$\boldsymbol{y} = \mathbf{X}\beta + \mathbf{Z}i\boldsymbol{u}i + \boldsymbol{\epsilon}i ,$$

which is a special case of the main problem class (1).

Shape constraints

We can impose shape constraints such as monotonicity, concavity, and convexity on splines. Constraints on splines have been developed in the past through reformulation techniques.8 The development in this section uses explicit constraints instead.

Monotonicity. Spline monotonicity across the domain of interest follows from monotonicity of the spline coefficients.6 Given coefficients

$$\beta = \begin{bmatrix} \beta_1 \\ \vdots \\ \beta_n \end{bmatrix},$$

the curve $f(t)$ is monotonically non-decreasing when

$$\alpha 1 \leq \alpha 2 \leq \cdots \leq \alpha n$$

and monotonically non-increasing if

$$\alpha 1 \geq \alpha 2 \geq \cdots \geq \alpha n.$$

The relationship $\alpha 1 \leq \alpha 2$ can be written as $\alpha 1 - \alpha 2 \leq 0$. Stacking these inequality constraints for each pair $(\alpha i, \alpha i{+}1)$ we can write all constraints simultaneously as

$$\underbrace{\begin{bmatrix} 1 & -1 & 0 & \cdots & 0 \\ 0 & 1 & -1 & \cdots & 0 \\ \ddots & \ddots & \ddots & \ddots & \vdots \\ 0 & \cdots & \cdots & 1 & -1 \end{bmatrix}}_{\mathbf{C}} \begin{bmatrix} \alpha_1 \\ \alpha_2 \\ \alpha_3 \\ \vdots \\ \alpha_n \end{bmatrix} \leq \begin{bmatrix} 0 \\ 0 \\ \vdots \\ 0 \end{bmatrix}.$$

These linear constraints are a special case of the general estimator (9) that allows $(\beta) \leq c_\beta$.

Convexity and concavity. For any twice continuously differentiable function: $f\colon \mathbb{R} \rightarrow \mathbb{R}$, convexity and concavity are captured by the signs of the second derivative. Specifically, f is convex if $f''(t) \geq 0$ is everywhere, and concave if $f''(t) \leq 0$ everywhere. We can compute $f''(t)$ for each interval, and impose linear inequality constraints on these expressions.

Enforcing linear tails. For large consumption with little data, we need the capability to ensure that the last segment of the spline is linear, with slopes that match the adjacent segment at the knot. The estimated spline is then fit to the data, subject to this specification. Priors on the tails can also be provided.

Figure B. Spline extrapolation. Left: linear extrapolation. Right: nonlinear extrapolation.

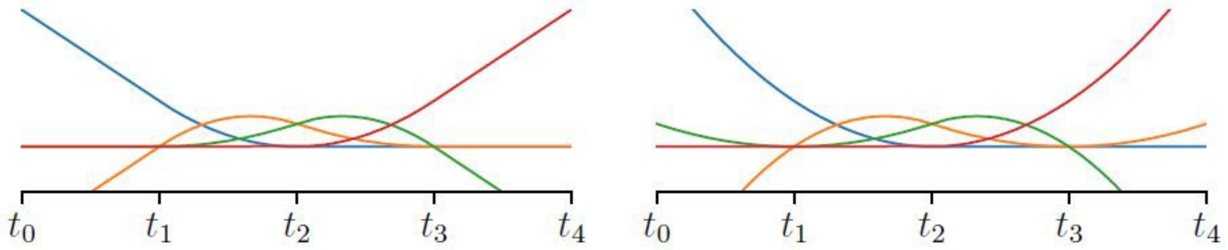

In general, using linear head and/or tail pieces to extrapolate outside the original domain or interpolate in the data sparse region is far more stable that using higher order polynomials, see Figure B. The figure shows symmetric linear tail modifications, but for the analyses in the paper we only impose a right linear tail shape constraint.

Posterior variance estimation

To obtain posterior uncertainty, we use a parametric bootstrap.9 Once we solve (9) to obtain estimates β and γ, we have a model distribution of the errors (1):

$$\boldsymbol{y}_i = \mathbf{F}_i\left(\hat{\beta}\right) + \mathbf{Z}_i \boldsymbol{u}_i + \boldsymbol{\epsilon}_i$$

We sample datasets from this distribution to generate full data sets $\{\boldsymbol{Y}\}^{j}$, for j=1,…,. For each dataset **Y**j, we then re-solve the fitting problem (9) to obtain estimates βj and γj, and the set $\{\beta j, \gamma j\}$ over all j allows us to estimate any posterior statistic we need.

In particular, the posterior set of dose-response curves is given by

$$\left\{f(t)_j + u_j^0\right\}$$

where $f(t)j$ is the curve obtained by using the re-fit value βj, and u_j^0 is a sample from $N\left(0, \gamma_j^0\right)$, the associated unexplained heterogeneity parameter.

References

1. Zheng P, Aravkin AY, Barber R, Sorensen RJD, Murray CJL. Trimmed Constrained Mixed Effects Models: Formulations and Algorithms. *arXiv:190910700 [math, stat]* 2019; published online Sept 23. http://arxiv.org/abs/1909.10700 (accessed Nov 15, 2019).
2. Aravkin A, Davis D. Trimmed Statistical Estimation via Variance Reduction. *Mathematics of OR* 2019; published online July 5. DOI:10.1287/moor.2019.0992.
3. Rousseeuw P. Multivariate estimation with high breakdown point. 1985. DOI:10.1007/978-94-009-5438-0_20.
4. Forouzanfar M, Afshin A, Alexander LT, Anderson H, Bhutta Z, Murray CJL. Global, regional, and national comparative risk assessment of 79 behavioural, environmental and occupational, and metabolic risks or clusters of risks, 1990-2015: a systematic analysis for the Global Burden of Disease Study 2015. *Lancet* 2016; **388**: 1659–724.
5. Wächter A, Biegler LT. On the implementation of an interior-point filter line-search algorithm for large-scale nonlinear programming. *Math Program* 2006; **106**: 25–57.
6. Boor C de. A Practical Guide to Splines. New York: Springer-Verlag, 1978 https://www.springer.com/gp/book/9780387953663 (accessed Nov 15, 2019).
7. Friedman JH. Multivariate Adaptive Regression Splines. *Ann Statist* 1991; **19**: 1–67.
8. Pya N, Wood SN. Shape constrained additive models. *Stat Comput* 2015; **25**: 543–59.
9. Efron B, Tibshirani RJ. An Introduction to the Bootstrap, 1 edition. New York: Chapman and Hall/CRC, 1993.

Global Burden of Disease World Regions

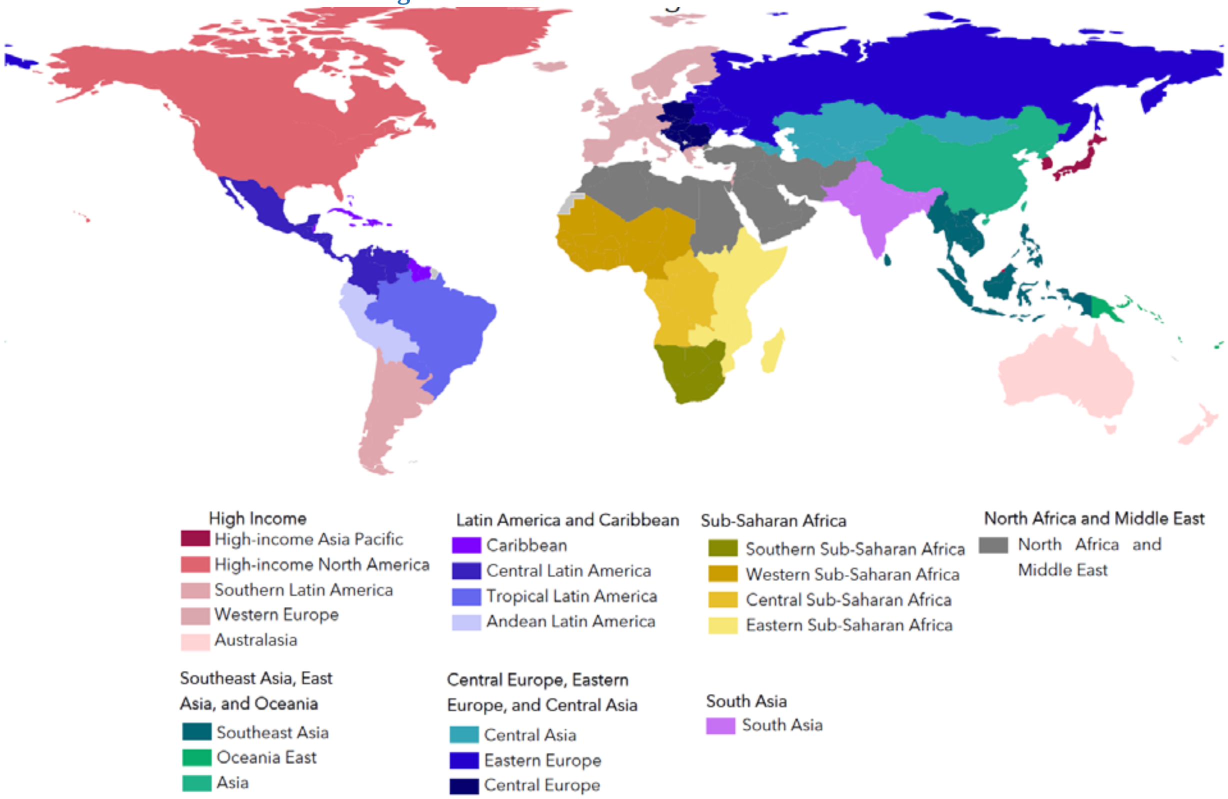

Supplement: Supplementary file 1 — Supporting information. [file TRC2-7-e12200-s001.pdf]
